# Concurrent oxygen evolution reaction pathways revealed by high-speed compressive Raman imaging

**DOI:** 10.1038/s41467-024-52536-7

**Published:** 2024-09-27

**Authors:** Raj Pandya, Florian Dorchies, Davide Romanin, Jean-François Lemineur, Frédéric Kanoufi, Sylvain Gigan, Alex W. Chin, Hilton B. de Aguiar, Alexis Grimaud

**Affiliations:** 1 Laboratoire Kastler Brossel, ENS-Université PSL, CNRS, Sorbonne Université, Collège de France, 24 rue Lhomond, Paris, France; 2 Department of Physics, Cavendish Laboratory, University of Cambridge, JJ Thomson Avenue, Cambridge, UK; 3 Department of Chemistry, University of Warwick, Coventry, UK; 4 Chimie du Solide et de l’Energie, UMR 8260, Collège de France, Paris, France; 5 Réseau sur le stockage Electrochimique de l’Energie (RS2E), Amiens, France; 6 Sorbonne Université, CNRS, Institut des Nanosciences de Paris, UMR7588, Paris, France; 7 Université Paris-Saclay, CNRS, Centre de Nanosciences et de Nanotechnologies, Palaiseau, France; 8 Université de Paris, ITODYS, CNRS-UMR 7086, 15 rue Jean-Antoine de Baïf, Paris, France; 9 Department of Chemistry, Boston College, Merkert Chemistry Center, Chestnut Hill, MA USA

**Keywords:** Electrocatalysis, Electrocatalysis, Raman spectroscopy, Energy, Electron transfer

## Abstract

Transition metal oxides are state-of-the-art materials for catalysing the oxygen evolution reaction (OER), whose slow kinetics currently limit the efficiency of water electrolysis. However, microscale physicochemical heterogeneity between particles, dynamic reactions both in the bulk and at the surface, and an interplay between particle reactivity and electrolyte makes probing the OER challenging. Here, we overcome these limitations by applying state-of-the-art compressive Raman imaging to uncover concurrent bias-dependent pathways for the OER in a dense, crystalline electrocatalyst, α-Li_2_IrO_3_. By spatially and temporally tracking changes in stretching modes we follow catalytic activation and charge accumulation following ion exchange under various electrolytes and cycling conditions, comparing our observations with other crystalline catalysts (IrO_2_, LiCoO_2_). We demonstrate that at low overpotentials the reaction between water and the oxidized catalyst surface is compensated by bulk ion exchange, as usually only found for amorphous, electrolyte permeable, catalysts. At high overpotentials the charge is compensated by surface redox active sites, as in other crystalline catalysts such as IrO_2_. Hence, our work reveals charge compensation can extend beyond the surface in crystalline catalysts. More generally, the results highlight the power of compressive Raman imaging for chemically specific tracking of microscale reaction dynamics in catalysts, battery materials, or memristors.

## Abstract (web summary)

Advancing the oxygen evolution reaction understanding requires probing catalysts’ microscale dynamics in operando but is highly challenging. Here, authors use compressive Raman imaging to unravel the charge compensation pathways in a crystalline oxygen evolution catalyst, α-Li_2_IrO_3_.

## Introduction

The oxygen evolution reaction (OER) is the anodic half-reaction generating protons and electrons necessary for the electrochemical synthesis, in aqueous conditions, of chemical fuels such as hydrogen or CO_2_-reduction products (CO, formic acid, ethylene etc). Boosting OER kinetics–a reaction involving the exchange of four electrons and four protons which can be more sluggish than its cathodic counterparts–is critical to ensure good performance for water or CO_2_ electrolysers^1,2^.

The OER mechanism can be strongly related to the structure of the catalyst^3–9^. For example, in amorphous and electrolyte penetrable OER catalysts, including, Co-Pi and derivatives, transition metal (oxy)hydroxides and several iridium-based catalysts, operando measurements have demonstrated that all metallic sites throughout the catalyst are redox active and exchange ions (protons) with the electrolyte^10–12^. In this case, charges accumulate during OER as a function of the applied potential and the catalytic current^13–15^. On the other hand, for non-porous, crystalline (primary particles) catalysts, only the outermost surface sites of the catalyst are subject to changes in oxidation state and participate in the charge balance^7^. These catalysts are more challenging to study in operando because only a small fraction of the material is undergoing changes during the OER^12^.

Recently, evidence has emerged to suggest that in some crystalline (non-porous/non-electrolyte permeable) catalysts (e.g., α-Li_2_IrO_3_ and LiCoO_2_) reaction of water and surface active sites can lead to bulk cation exchange in the catalyst, i.e., bulk metallic redox active sites may be involved in the OER^16–18^. However, beyond this, very little is known regarding the charge compensation dynamics in these promising OER catalysts. For example, it is unclear how ions actually propagate through these crystalline catalysts as a function of applied potential and/or current density. If the intercalating cations propagate much slower than the rate of O_2_ evolution, the charge compensation itself will mostly still be limited to surface sites in contact with the electrolyte. In other words, the reaction between surface sites and water will mainly serve to quench the accumulated charges upon rest and stabilize the surface. On the contrary, if the velocity of cations is high compared to the rate of O_2_ evolution, intercalation may proceed through a non-negligible portion of the particle, actually extending the charge compensation to the bulk.

The major challenge of deciphering the charge compensation pathways in these dense, crystalline catalysts is the need for in-situ characterisation with high time-, space- and chemical-resolution^19,20^ such that the extent to which cations exchange upon OER can be followed^21^. Indeed, generally for catalysts storing charges, a release of potential in the presence of electrolyte will quench the charge, meaning ex-situ methods cannot be used to capture the OER dynamics or even the real active state of the catalyst.

Spontaneous Raman spectroscopy and surface enhanced^22,23^ or time-gated variants^24^ are widely used for tracking electrode dynamics with chemical bond-selectivity^25^. Indeed, a number of previous works have used Raman spectroscopy specifically for operando investigation of the OER mechanism^26–29^. However, the low signals in spontaneous Raman have hampered obtaining the necessary spatial and temporal resolution for real-time Raman imaging of operating electrodes. Furthermore, many electrochemical systems demand probing in the 300 to 1200 cm^−1^ spectral region, which is challenging for more sensitive coherent Raman variants^30^. The data loads and relative expense in terms of equipment^31^, as compared to other optical methods, e.g., reflection microscopy^32,33^, have additionally limited the use of Raman as a laboratory tool for imaging inside electrochemical systems. Here, we alleviate these limitations by using the compressive Raman imaging scheme^34–36^. This approach has been used previously for non-time resolved Raman imaging of static media (pharmaceutical powders^37^, micro-plastics^38^, vesicles^39^) but, to the best of our knowledge, is yet to be explored for imaging dynamic processes, particularly in the context of electrochemistry.

In this work, we push (in terms of time-resolution and operando conditions) compressive Raman imaging to elucidate the charge compensation pathways in charge-accumulating OER catalysts. Tracking changes in the in- and out-of-plane vibrational stretches in a layered α-Li_2_IrO_3_ catalyst (as well as other crystalline catalysts with cationic exchange properties, such as LiCoO_2_), we follow the potential-dependent dynamics of cation intercalation upon cycling in a range of electrolytes. Importantly, by measuring the rate of gas evolution from individual catalyst particles (extracted using optical imaging) and the propagation of different phase fronts inside the catalyst (under various electrochemical conditions), we are able to provide a more definitive answer regarding the predominant charge compensation pathways during OER, at different potentials/current densities, in this novel class of catalysts.

## Results

**Fig. 1:**
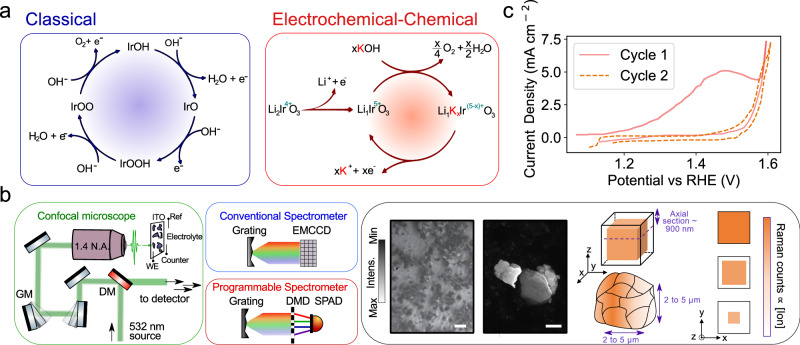
Spontaneous Raman imaging setup and agglomerate particles imaged. **a** Classical adsorbate mechanism for water oxidation on the surface of iridium oxide catalysts (blue box) and proposed mechanism for water oxidation with α-Li2IrO3 (red box). **b ** (Green box) Cartoon schematic of confocal Raman microscope. The 532 nm laser is scanned across a sample using galvanometric mirrors (GM) with the excitation and Raman light separated by a dichroic mirror (DM). Either a conventional spectrometer (equipped with an EMCCD (conventional detector; blue box)) or a programmable spectrometer (equipped with a DMD (digital micromirror device) and avalanche photodiode (SPAD; red box)) are used to measure the Raman signal. Light is focussed onto the sample through the back side of an ITO or Ti-coated coverslip, which acts as the working electrode (WE). (Black box) Brightfield image of α-Li_2_IrO_3_ agglomerate particles (dark features) dispersed with Nafion onto ITO slide (left). The scale bar is 10 μm. (Centre) Scanning electron microscopy image of a ~ 1 (left) and ~ 2.5 μm (right) size α-Li_2_IrO_3_ agglomerate particle (without Nafion). The scale bar is 1 μm. (Right) Cartoon schematic of agglomerate particle simplified to a cubic shape. The 900 ± 200 nm axial resolution of our setup means we probe a 3D section of the particle bulk. When viewed in 2D projection (far right), an isotropic 3D deintercalation process gives rise to distinct patterns. The white to orange colour bar depicts the degree of a given ion intercalation phase. **c** Cyclic voltammogram of α-Li_2_IrO_3_ showing the first two cycles. The scan rate is 10 mV/s. The current is normalised to the geometric surface area that the electrolyte covers (~ 75 mm^2^; see “Methods”). The voltage is not iR-corrected.

Figure 1a shows a schematic of the spontaneous Raman imaging setup. Excitation of the samples is performed with a 532 nm laser which is focussed onto the sample using a 1.4 numerical aperture objective (spatial resolution ~ 300 nm). As the Raman signal scales as 1/λ^4^, this wavelength provides a good balance between the signal and any potential sample damage from the Raman laser pump. The laser beam is rapidly raster scanned across the sample using galvanometric mirrors with the inelastic Raman backscatter light guided to spectrometers. For measurements which are only spectrally resolved, i.e., no imaging, we use a ‘conventional’ spectrometer equipped with an electron-multiplying charge-coupled (EMCCD) camera to acquire the whole vibrational spectrum in a single shot. For Raman imaging requiring higher readout and reduced data loads, we exploit the compressive Raman imaging method. Here, a programmable spectrometer equipped with a single-pixel detector–in our case, a single-photon avalanche photodiode (SPAD)–is used^39^. Such a framework allows us to significantly compress the spectral data during the measurement stage and remove the excess noise of conventional cameras when imaging at high speed^31^. As the beam is raster-scanned across the sample, optimal filters loaded into the programmable spectrometer allow selection of the Raman scattered light associated with each of the predetermined species of interest and background (see Supplementary Note 1 for further details). Orthogonality between filters, whose widths are sufficiently large enough to account for any spectral shifts, ensures no overlap between spectral channels. In this way, chemical imaging can be achieved at up to ~ 0.3 fps (50 μm^2^). A maximum laser power of ~ 30 mW (before the objective) is used in all experiments to minimise sample degradation (see Supplementary Note 2).

α-Li_2_IrO_3_ particles are dispersed (with Nafion) onto thin transparent cover slides with a conductive coating (ITO or 10 nm Ti, depending on the exact experiment, with negligible difference in cycling/Raman response between substrates, see Supplementary Note 2). 1 M KOH solution, which is used as the electrolyte, is placed in ~ 15 × 5 mm diameter, inert silicone wells to allow for multiple experiments per substrate. Potentials are applied using a standard three-electrode setup consisting of a Pt wire counter electrode and an Ag/AgCl reference electrode (see “Methods” for further details). As is often observed for layered intercalation compounds synthesized by high-temperature solid-state routes, large 2 to 5 μm α-Li_2_IrO_3_ agglomerate particles are observed, which comprise primary particles (which are a few 100 s nm to 1 μm in size and can be randomly orientated in the agglomerate) as shown by the scanning electron microscopy images and schematic in Fig. 1b (see also Supplementary Note 3).

We note that for secondary crystalline catalysts made of primary particles, porous gaps between the primary particles can also be penetrable to the electrolyte. However, our SEM imaging and Raman signals show little evidence of significant porous Nafion binder/gaps, etc, between the crystalline primary particles. Hence, the following interpretations consider the ‘inside’/bulk of the secondary particles as non-electrolyte permeable/non-porous (see Supplementary Note 4 for further discussion), and when referring to the ‘surface’ explicitly refer to that of secondary particles made from primary micro-crystals.

Based on our microscope objective and the optical attenuation properties of the α-Li_2_IrO_3_, the penetration of the laser beam into the samples is ~ 900 + /− 200 nm. Using optical coherence tomography^40^ (see Supplementary Note 4), we estimate particle thicknesses of ~2 to 5 μm, indicating that the Raman signals originate from up to ~ 30% of the particle volume, as depicted in the cartoon in the right part of Fig. 1b. The agglomerates we image are taken to have limited curvature within our optical resolution (i.e., the entire agglomerate is in focus in a single imaging plane; see Supplementary Note 5 for further justification), hence allowing us to approximate them as cubes. The large refractive index mismatches between agglomerates/electrolytes and our imaging system^41^, as well as the presence of Nafion, make bright-field optical imaging of agglomerates challenging. The chemically specific Raman signal is hence used to define the agglomerate boundaries, centre-of-mass, etc. Whilst some agglomerates present are > 5 μm and/or not flat, to be systematic, we limit ourselves to drawing conclusions only from agglomerates imaged that satisfy the above criteria (see Supplementary Note 5 for further discussion).

Similar electrochemical behaviour is observed with this setup, as shown in Fig. 1c, as compared to that recorded using a classical three-electrode cell with the catalyst particles drop-casted onto a rotating disk electrode^18^. First, anodic activation is observed (current peak centred at ~ 1.45 V vs the reversible hydrogen electrode (RHE)), which is associated with the deintercalation of Li^+^ from α-Li_2_IrO_3_ to a stoichiometry close to α-Li_1_IrO_3_ (step 1); we refer to this compound as the activated phase in the following. At more anodic potentials, OER current is measured. Once formed, the oxidised form of the catalyst α-Li_1_IrO_3_ can chemically react with KOH to form a birnessite phase containing hydrated potassium cations (step 2), this reaction being associated with the evolution of molecular oxygen as we previously demonstrated; we refer to this latter compound as α-Li_1_K_x_IrO_3_ henceforth (inserted potassium cations are hydrated but we omit water molecules in the formula for simplification)^18^. The surface sites of α-Li_1_IrO_3_ can also solely participate in the OER via a mechanism involving surface charge balancing, commonly referred to as the adsorbate evolution mechanism^7^, that is often observed for non-porous crystalline catalysts, (step 2’).

1 $${\alpha -{Li}}_{2}{{Ir}}^{4+}{O}_{3}\,\to {\alpha -{Li}}_{1}{{Ir}}^{5+}{O}_{3}+{e}^{-}+{{Li}}^{+}$$

2 $${\alpha -{Li}}_{1}{{Ir}}^{5+}{O}_{3}+x{{OH}}^{-}+x{K}^{+}\,\to \,{{Li}}_{1}{K}_{x}{{Ir}}^{5-x}{O}_{3}+\frac{x}{4}{O}_{2}+\frac{x}{2}{H}_{2}O$$

2’ $$4{{OH}}^{-}\to {O}_{2}+4{e}^{-}+2\,{H}_{2}O$$

However, as discussed, the extent to which cation intercalation (step 2) extends on the reaction between the catalyst and water and its exact potential range of operation remains unknown (see Supplementary Note 6). Ex-situ techniques could only reveal that after the initial anodic scan, hydrated K^+^ can reversibly exchange at potentials concomitant to the OER^18^, but no insights on the rates/dynamics of such exchange at anodic potentials, could be gained from ex-situ studies.

**Fig. 2:**
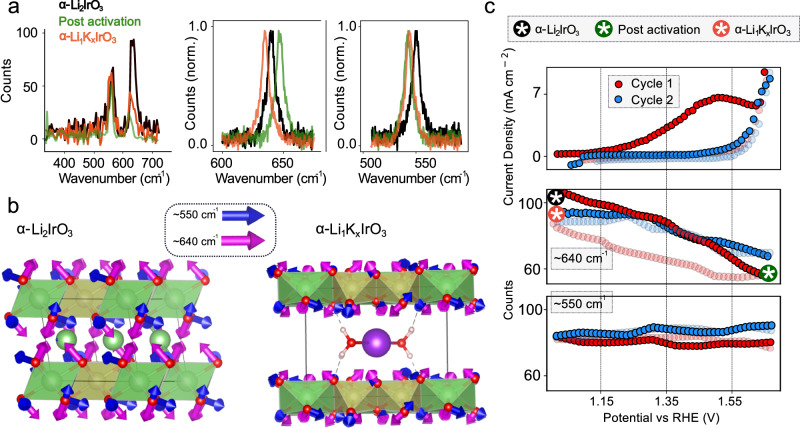
Raman signatures of the different phases. **a** (Left) Low resolution (~ 15 cm^−^^1^ spectral resolution) Raman spectra experimentally recorded for α-Li_2_IrO_3_, post oxidation/delithiation (‘post activation’ i.e., formation of α-Li_1_IrO_3_) and α-Li_1_K_x_IrO_3_. (Centre and middle). Normalised high resolution (~ 1 cm^−^^1^ spectral resolution) Raman spectra of α-Li_2_IrO_3_, post oxidation/delithiation (‘post activation’) and α-Li_1_K_x_IrO_3_. **b** Crystallographic structures of α-Li_2_IrO_3_ and α-Li_1_K_x_IrO_3_ with arrows highlighting computed atomic motion corresponding to Raman modes at ~ 550 cm^−^^1^ and ~ 640 cm^−^^1^. Red – Oxygen, Yellow – Ir, Green – Li, Purple – K, White – H. **c** Cyclic voltammograms were recorded over the first two cycles of α-Li_2_IrO_3_ with the corresponding intensity of the 640 cm^−^^1^ and 550 cm^−^^1^ modes. Cycling was performed at 10 mV/s due to the acquisition speed limitations of the Raman setup. The first and second cycles are shown in red and blue, respectively, with dark circles showing an anodic sweep and faded circles the cathodic one. Coloured circles with a white asterisk mark phases formed at respective start or end-points of a given cycle. The voltage is not iR-corrected.

To investigate the OER mechanism, Raman spectra were initially collected for α-Li_2_IrO_3_, i.e., before cycling (pristine), after the initial activation/delithiation and after cycling, during which potassium is intercalated to form α-Li_1_K_x_IrO_3_ (7 cycles; Supplementary Note 7). As shown in Fig. 2a, the Raman spectrum of α-Li_2_IrO_3_ shows two peaks at ~ 550 cm^−1^ and ~ 640 cm^−^^1^, in agreement with previous studies^42,43^. Activation/delithiation induces a modification of the Raman spectrum for the catalyst with a ~ 50% dimming of the 640 cm^−^^1^ mode and ~ 10% dimming of the ~ 550 cm^−^^1^ mode. High-resolution Raman spectroscopy (~ 1 cm^−^^1^ spectral resolution; right side of Fig. 2a) shows a red shift of both the 640 cm^−^^1^ and 550 cm^−^^1^ peaks on delithiation (see Supplementary Note 8). On cycling the material in KOH to form α-Li_1_K_x_IrO_3_, the Raman mode at 640 cm^−1^ grows in intensity, but only to ~ 40% of the intensity of that in α-Li_2_IrO_3_ (Fig. 2a) and blue shifts by ~ 15 cm^−1^. The 550 cm^−1^ mode intensity and peak position remain relatively unperturbed as compared to the activated form (far right panel of Fig. 2a).

Density functional theory (DFT) calculations were carried out to provide a real-space understanding of the atomic displacements associated with the two observed Raman peaks for both the pristine α-Li_2_IrO_3_ compound, the delithiated form of the material and the catalyst containing potassium after reaction with KOH (Supplementary Note 9). In all cases, the mode at ~ 550 cm^−1^ corresponds to stretching/scissoring of the Ir-O bond in the plane of IrO_6_ edge-sharing octahedra layers, whereas the higher frequency mode at ~ 640 cm^−^^1^ corresponds to an Ir-O stretch out of the layer plane. From a DFT viewpoint, α-Li_2_IrO_3_ and the associated hydrated and (de)intercalated forms of the material are metallic^44^. Hence, DFT only provides information on vibrational mode frequencies, not intensities. Indeed, for a system such as the one considered in this work that contains a large number of atoms in the unit cell, spin-orbit coupling^45^, and hydrated cations^46^, full vibrational theoretical treatment is exceptionally challenging using current methods. On potassium intercalation, the computed in-plane 550 cm^−^^1^ mode shows relatively little change in spectral position, whereas, at higher frequencies around 640 cm^−^^1^, the computed (blue) shifts are larger. In general, the larger changes in the 640 cm^-1^ mode as compared to that at 550 cm^-1^ can be rationalised by realising that the former involves modifications along the *c*-direction which are much stronger (increase of more than 1 Å for the interlayer distance upon hydrated K^+^ intercalation, as previously probed by XRD^18^) when compared to those in the *ab* plane. These observations are consistent with previous studies of layered intercalation compounds where modes of *A*_1g_-like symmetry (in-plane, ~ 550 cm^−^^1^) and *E*_g_-like symmetry (out-of-plane, ~ 640 cm^−1^) drop in intensity on deintercalation^47^. Furthermore, the penetration depth of the Raman laser does not change between the native, activated/delithiated or potassium intercalated forms of the catalyst^48,49^ (see Supplementary Note 4), supporting the assertion that the changes in Raman intensity arise from structural changes as opposed to ‘skin’ (conductivity) effects^50^.

**Fig. 3:**
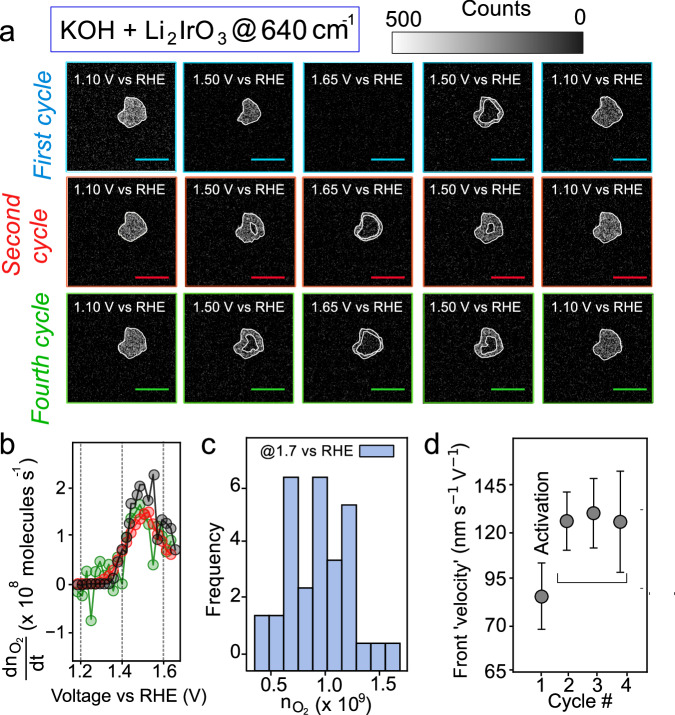
Operando Raman imaging and gas bubble imaging. **a** Raman intensity images of 640 cm^-1^ mode at selected potentials during the first, second and fourth electrochemical cycles of α-Li_2_IrO_3_ using KOH as the electrolyte. Cycling is performed at 4 mV/s (see Supplementary Videos 1–2 for other cycles and Supplementary Videos 3–4 and Supplementary Note 13 for repeats over more particles). The scale bar is 5 μm. **b** Rate of evolution of O_2_ molecules from three different individual agglomerates, obtained using bright-field imaging of gas bubble evolution. **c** Histogram of a number of O_2_ molecules generated from individual agglomerates on reaching 1.7 V vs RHE. 30 agglomerates are imaged. **d** Pseudo ‘velocity’ of phase front, which is dominated by Li^+^ deintercalation during the initial anodic scan and K^+^ deintercalation in scans 2 to 4. All values are extracted from the anodic scan (see Supplementary Note 15 for the method). Error bars represent both measurement and fitting uncertainties.

To further understand the link between cation exchange and changes in the Raman spectrum, operando measurements using the conventional spectrometer were carried out with spectra measured during cyclic voltammetry in 1 M KOH (aq) electrolyte (Fig. 2c). First, we remark that the Raman experiments in Fig. 2 and Fig. 3 below are done at scan rates of 10 mV/s (for non-spatially resolved Raman measurements) and 4 mV/s (for spatially resolved measurements) to allow for effective synchronisation with our detectors. During the initial anodic scan (1.1 V to 1.7 V vs RHE), an immediate dimming of the 640 cm^−1^ (ΔCounts ~ 40%), and to a much lesser extent, the 550 cm^−1^ (ΔCounts ~ 5%) peak is observed. These observations (and also the peak shifts we observe) are consistent with previous literature for charging of layered metal oxides used in Li-ion batteries^47^ and the measurements described above, confirming that during the initial anodic scan delithiation occurs. Upon the subsequent cathodic scan, the 640 cm^−^^1^ peak intensity increases again, however it is to an intensity lower than that of the initial α-Li_2_IrO_3_ phase. The overall behaviour recorded during the initial cycle is consistent with an initial oxidation/delithiation to form a phase with a chemical composition close to α-Li_1_IrO_3_, followed by the intercalation of hydrated potassium as the potential is dropped. After the first scan, subsequent anodic scans in the same potential range result in a dimming of the 640 cm^−1^ mode by around ~ 40% and only a small (~ 5–10%) increase in the intensity of the 550 cm^-1^ mode (see Supplementary Note 4 for changes in peak positions). These changes in mode intensity are reversible, i.e., the intensity of the 640 cm^−1^ (550 cm^−1^) increases (decreases) during the corresponding cathodic scans/reduction. We note that following the initial cycle, the intensity of the 640 cm^−1^ only begins to drop (increase) in the anodic (cathodic) scan after ≈ 1.34 V vs RHE (as also confirmed by taking the derivative of the changes in mode intensity; see Supplementary Note 10) indicating an oxidation event at the potential previously assigned to the onset of K^+^ deintercalation^18^. This is direct visualization that the active form of the catalyst, i.e., α-Li_1_IrO_3_, is regenerated electrochemically at a potential concomitant with the OER. Based on these observations, we can confirm that the intensity of the 640 cm^−1^ mode can be used to track reversible cation intercalation and distinguish between the various phases.

Interestingly, when cycling in 1 M LiOH, no drastic modification of the 640 cm^−1^ (or 550 cm^−1^) mode intensity is observed after the initial anodic scan (Supplementary Note 11). In other words, no Li^+^ intercalation occurs under these conditions due to the larger hydrodynamic radius of Li^+^ as compared to K^+^, in agreement with our previous findings^18^. Furthermore, for crystalline IrO_2_ for which no bulk cation exchange was reported, two broad Raman bands around 500 cm^−1^ and 700 cm^−1^ are observed, and cycling in the potential range of 1.1 to 1.7 V vs RHE does not result in significant modification of the intensity of both bands (Supplementary Note 11). The above observations are consistent with measurements of over > 40 catalyst particles (agglomerates). However, we do not compare the absolute changes in Raman counts between agglomerates due to the optical (focus) and sample (size, nanoscale orientation) effects (see Supplementary Note 11).

Having identified vibrational signatures of cation (de)intercalation, images of the integrated intensity of the 640 cm^−1^ band across a single catalyst particle (and 550 cm^−1^) were collected using compressive Raman imaging (imaging through the backside of the substrate) during each step of increasing and decreasing potential. In this way we can access the spatial distribution of phase fronts associated with the different cationic processes/material stoichiometries. The spectral integration window was chosen such that changes in the peak position did not influence the intensities observed (see Supplementary Note 8 for measurements where the window size/position is varied). During the initial anodic scan, for the 640 cm^−1^ mode, a front of diminishing intensity is observed to move from the surface of a (secondary agglomerate) particle in contact with the electrolyte to the core of the particle (Fig. 3a, top row) as the potential is increased to 1.65 V vs RHE (see also Supplementary Videos 1–4). If we consider that our densely packed agglomerates have distinct surface and bulk reactivity, a ‘toy model’ can be used to rationalise the Raman images. Assuming a 3D structure with limited curvature, with overall delithiation from the surface through the core, in 2D projection: (i) the surface will uniformly drop in Raman intensity, (ii) the bulk layers will lose Raman intensity first from their edge and then their centre. The signal we observe is the sum of these two contributions and hence has an overall core-shell pattern, as sketched in Fig. 1b (far right panel). A similar logic can be applied to the initial cathodic scan. We note that quantifying the relative contribution of bulk and surface signals is challenging. The former likely dominates due to its greater volume percentage, but we refrain from an overall assignment.

The pattern of spatial motion we observe is in line with that previously observed for the delithiation of battery materials (including secondary particles/agglomerates) such as Li_x_CoO_2_ (LCO), Li_x_FePO_4_ and Li_x_Ni_0.8_Mn_0.1_Co_0.1_O_2_ using reflection microscopy and synchrotron imaging^51–55^. During the following cathodic scan, the integrated intensity of the 640 cm^−1^ mode increases from the particle edges in a pattern and intensity consistent with the intercalation of potassium cations, as discussed in Fig. 2a. This conclusion is reinforced by noticing that in LiOH, no change is observed during the cathodic scan (Supplementary Note 11).

For the second anodic cycle, a different spatial pattern is spotted (second row of Fig. 3a). The core of the particle is observed to lose Raman intensity before the exterior during the anodic scan with a shell of higher intensity remaining on reaching 1.65 V vs RHE, in an opposite manner to the ‘classical’ shrinking core pattern found during the first cycle. A similar logic to the first anodic scan can be applied to rationalise the images: at the very outermost (top) surfaces, changes in signal will be uniform (although not necessarily monotonic with potential), whereas signal from the bulk volume of the material will change differently at the edges as compared to core. The sum of these two effects gives the overall pattern. During the cathodic scan, the agglomerate is slowly replenished in potassium, and a homogeneous intensity for the 640 cm^−1^ mode is eventually found when back returning to the open circuit voltage (i.e., 0 mA cm^−2^). This process is observed across numerous cycles, as shown in the bottom row of Fig. 3a, with the shell being consistently higher in Raman intensity i.e., rich in potassium (reduced), while the core is depleted. In comparison, no such change in intensity for the 640 cm^−1^ vibration mode (and the 550 cm^−1^ one) was observed when performing imaging in LiOH, apart from during the initial delithiation occurring in the first cycle (see Supplementary Note 11), confirming that it is related to potassium (de)intercalation from the particles. We note that when cycling electrochemically LiCoO_2_, another crystalline layered compound known to have an OER mechanism involving cation intercalation^56^, similar, cycle-dependent, patterns of behaviour are observed (see Supplementary Note 12).

Our observation of changes throughout the agglomerate particle suggests that α-Li_2_IrO_3_ (and other crystalline, ion-permeable catalysts) store charges as a function of potential during the OER but, unlike for classical dense and crystalline catalysts (e.g., IrO_2_), charges are stored in bulk following the reversible (de)intercalation of cations. Such charge compensation involving every metallic redox centre is normally limited to films of amorphous catalysts for which the electrolyte can penetrate through the thickness of the film^10^.

In order to understand the core-shell structure forming in the second and subsequent anodic scans, further measurement and analysis are required. Using bright-field imaging through the electrolyte^57,58^ we can track and quantify the evolution of O_2_ gas from individual agglomerates. As shown in Fig. 3b, we find that the rate of production of O_2_ gas bubbles increases up to 2 × 10^9^ O_2_ molecules s^−1^ as the potential is ramped from ~ 1.3 to 1.5 V vs RHE. After 1.5 V vs RHE, the measured gas evolution rate slows. This could be due to geometric factors related to the size of the large bubbles forming on the surface (10 μm> diameter hemispherical bubbles^59^). Nonetheless, between 0.5 and 1.5 × 10^9^ O_2_ molecules are estimated to be generated per agglomerate by 1.7 vs RHE on the anodic scan. Gas evolution occurs statistically more frequently from the agglomerate edges (~ 75% of the time; see Supplementary Note 14) versus the top surface, but caution should be taken in correlating this behaviour with activity due to the presence of Nafion in the system and millisecond time resolution/micron spatial resolution of the experiments, limiting identification of the exact nucleation centres.

From the operando Raman imaging data, a pseudo phase front ‘velocity’ associated with cation (de)intercalation can be extracted for our scan rates. Line cuts of the Raman image, passing through the particle boundary, are taken over four orthogonal rectangular strips. The line-cuts have a sigmoidal shape, whose centre moves as the front propagates. Plotting the (fitted) sigmoid centre versus time, in a given voltage range depending on the scan number (see Supplementary Note 15) from the particle boundary (or from the centre-of-mass), allows for the extraction of phase-front ‘velocity’ which we normalise by the voltage (see Supplementary Note 15 for further details). First, upon initial oxidation and delithiation of α-Li_2_IrO_3_ to α-Li_1_IrO_3_, a phase front ‘velocity’ of ≈ 82 ± 20 nm s^−1^ V^−1^ is determined. During the subsequent cycles upon which hydrated K^+^ cations are (de)intercalated, a front ‘velocity’ of ≈ 120 ± 20 nm s^−1^ V^−1^ is measured, which does not change significantly between cycles (Fig. 3d). This latter value is extracted by examining the propagation of the intensity front from the centre-of-mass of the agglomerate by a constant amount (~ 1 μm) and matches well also with the ‘velocity’ obtained for the intercalation of potassium during the first cathodic scan (see Supplementary Note 15). The decay of the sigmoidal line cuts (length of the curve that is between 20% and 80% of the maximum) gives insight into the nature of the phase boundary between the intercalated and deintercalated material. For the first anodic scan, the boundary between the lithiated and delithiated phase remains relatively sharp (~ 400 nm, i.e., close to our spatial resolution), which could indicate a biphasic delithiation mechanism. In contrast, during the potassium de-intercalation, the boundary starts off at ~ 400 nm wide but broadens to up to ~ 1.8 μm in width (see Supplementary Note 15) during the scan, suggesting a solid-solution type behaviour^60^. Repeating Raman imaging experiments at slower scan rates of (0.4 mV/s) results in qualitatively similar spatial patterns to those observed in Fig. 3 (see Supplementary Note 16). This suggests at low scan rates (up to 10 mV/s) the intercalation of potassium cations into the bulk of the catalyst, spatial patterns and behaviour only depends on the applied potential. Whilst it would be interesting to examine behaviour at higher scan rates, the challenges associated with Raman imaging (synchronised) to electrochemistry at such speeds preclude measurement. Indeed, even purely electrochemical scan rate-dependent experiments (with rotating disk electrodes) provide little kinetic insight due to bubble formation and electrode surface blockage with this highly active electrocatalyst (Supplementary Note 16).

**Fig. 4:**
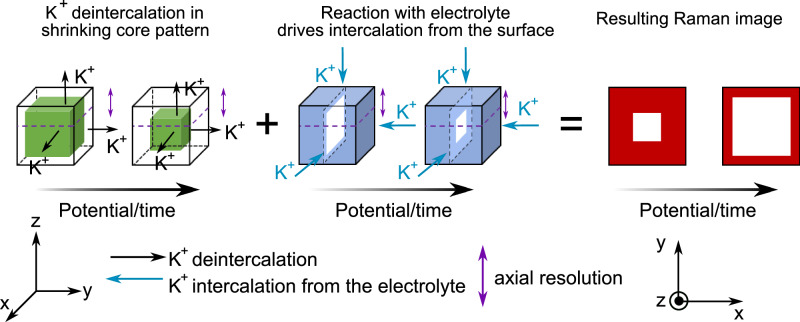
Simplified model of K^+^ (de)intercalation processes at the origin of the observed Raman images. Cartoon schematic summarising how competition between bulk particle deintercalation (in a shrinking core pattern) and intercalation of K^+^ from the electrolyte (starting from the edge of particles) gives rise to the observed spatial distribution of Raman intensity in the images after the first scan. We note this is a qualitative model and there are likely important other effects, e.g., non-uniform diffusion across the particle volume, that need to be considered for fully modelling the results.

Directly, comparing gas evolution rates with the phase-front velocities is challenging due to the multi-particle, 3D nature of agglomerates. However, one can note that the rate of O_2_ generation is increasing against applied potential while the cation (de)intercalation ‘velocity’ remains constant (see Supplementary Note 14). This observation, coupled with the unusual (as compared to previously observed ‘core-shell’ or ‘wave-like’) patterns of ion (de)intercalation after the first scan, suggests that several competing processes are occurring. We rationalise the observations as follows. During the (≥ 2) anodic scans, K^+^ deintercalation is driven, resulting in a decrease in the intensity of the 640 cm^−1^ mode and a shrinking-core type spatial pattern on the particle (i.e., from the edges of the particle first, like the initial Li^+^ deintercalation spotted during the first anodic scan). However, the oxidised particles simultaneously react chemically with the KOH electrolyte, re-intercalating K^+^ into the particle, resulting in an increase in the Raman intensity starting from the edges. The competition of these two effects of K^+^ entering and leaving the particle gives rise to the observed spatial intensity pattern, as summarised in the qualitative model in Fig. 4. However, at high potentials, the rate of gas-evolution is too high for K^+^ exchange, and the cation exchange pathway is too slow to participate in the charge compensation. Consequently, at high overpotentials, charge compensation occurs only at sites located near the surface, whereas at lower overpotentials, it can extend to the bulk.

**Fig. 5:**
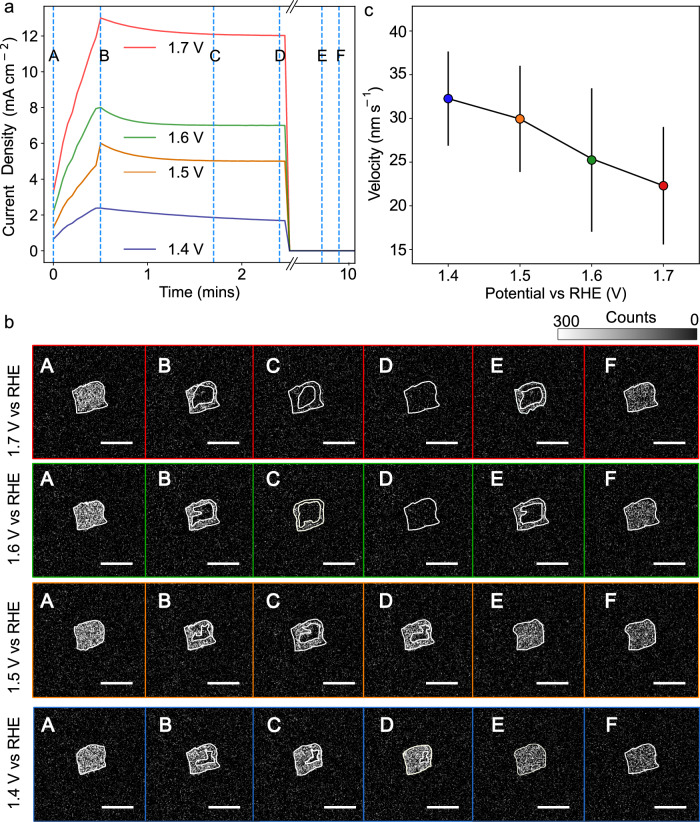
Potentiostatic holding experiments. **a** Ramp to potentials between 1.4 V and 1.7 V vs RHE followed by a 2 min potential hold at the given potential and then a spontaneous release of the potential for 8 mins. **b** Raman images integrating the intensity of the mode at 640 cm^−1^ for a single agglomerate particle at set points (labelled A to F in panel a) during the potential ramp, hold and release. The scale bar is 5 μm. **c** Velocity of the phase front associated with K^+^ ion diffusion back into the catalyst following release of the potential. Velocities are extracted from the spatial motion of the 640 cm^−1^ intensity front in the temporal region D to F in panel (**a**). Error bars are derived from repeat measurements over 35 particles and fitting errors.

To investigate further this assertion of a potential dependence in the charge compensation pathways, potentiostatic holding experiments were carried out at different potentials after two initial cycles of the catalyst (Fig. 5a and Supplementary Video 5–8). Ramping up to and holding at a potential of 1.6 and 1.7 V vs RHE for 2 minutes (high overpotentials associated with high rates of O_2_ evolution), Raman imaging (Fig. 5b) reveals that the full activation of the particles is not readily complete (images B and C). A potassium-rich shell is formed during the initial part of the holding, as previously observed during the CV scans (Fig. 3), before disappearing to reveal a fully oxidised particle at the end of the 2-minute hold (image D). Hence, despite the charged particles having sufficient oxidative power to chemically evolve oxygen and insert potassium, potassium cations do not have sufficient time to diffuse into the bulk of the particle (because of the high O_2_ evolution rates), and the particles are eventually fully deintercalated. The OER thus proceeds on the periphery of fully charged agglomerate particles, and charge compensation is limited to surface redox active sites. Eventually, upon relaxation (release of the potential), potassium intercalates, starting from the surface (images E and F). For potentials below that threshold, but sufficiently large for potassium to deintercalate, i.e., 1.4 and 1.5 V vs RHE, the centre of the particles is first depleted in potassium, alike at higher potentials (images B and C). However, even after an activation time of 2 min, the shell does not disappear, and the surface (and near-surface layers) remain rich in potassium owing to constant intercalation of potassium by chemical reaction with the electrolyte, revealing that the core-shell pattern reflects a quasi-static equilibrium (image D). We note that following the potential hold, the velocity of the intensity front corresponding to intercalating potassium (calculated using a similar approach as in Fig. 3d (see also Supplementary Note 16)) is approximately independent (slight decrease) of the previous holding potential and is in the order of ≈ 25 ± 7 nm s^−1^ (Fig. 5c; error accounts for measurement and fitting errors and varies depending on the exact potential), similar to the non-voltage normalised velocity we obtain during CVs (≈ 30 ± 5 nm s^−1^). This finding adds further weight to the notion that we are imaging concurrent pathways. At low current density (potentials), potassium exchange and intercalation are sufficiently fast to sustain the charge compensation during OER, and the surface is found rich in potassium. However, at high current densities (potentials), potassium intercalation is not fast enough to provide a bulk pathway for charge compensation, and charge compensation is therefore limited to the surface sites of the catalyst. We note that due to the multi-particle nature of the agglomerate particles we study, inter-agglomerate reaction heterogeneities and the complex electrochemical environment, we cannot distinguish the individual properties (velocities and hydration extents) of K^+^ simultaneously leaving and entering the particles that give rise to this pattern. Indeed, for such a complex system, even simple rate or phase-field models^61^ remain beyond reach, with electrochemical analyses such as cyclic voltammetry providing limited insight. This highlights the importance of moving towards chemically sensitive time-resolved imaging for the understanding of such electrochemical systems.

## Discussion

In summary, using operando Raman imaging, we have demonstrated the interplay between two charge compensation pathways occurring upon OER for α-Li_2_IrO_3_ (and LiCoO_2_), non-porous, non-liquid electrolyte permeable, crystalline catalysts. At high current densities, the oxygen evolution rate is too high for cation exchange to occur substantially, and only the surface (and first few surface layers) redox active sites are involved in the charge compensation upon OER. At lower overpotentials, the oxygen evolution rate becomes slow enough for cations to intercalate, and the charge compensation extends to the bulk of the catalyst. Whilst we have focussed on two systems here, our results are likely applicable to other layered electrocatalysts and also in acidic media. Indeed, in materials like β-IrO_3_ in H_2_SO_4_ electrolyte, proton insertion into the structure has shown to be concomitant with the chemical generation of gaseous oxygen^14^.

More generally, our results reveal the power of computational microscopy and operando Raman imaging using the compressive sensing framework. Here, the data load and analysis typically associated with hyperspectral imaging is significantly reduced, speed and sensitivity are boosted, and cost and need for expensive cameras removed^35,39,62^. The Raman spectrum of the system tackled here is relatively simple, but with appropriately designed filters^63,64^ the above methods can be readily applied to more complex systems, where there may be multiple spectrally overlapped species, without losing sensitivity^39,64^. The unique (material agnostic) liquid and solid phase chemical sensitivity, and potential to be quantitative (Raman intensity is proportional to concentration), means such methods will find great use in probing other electrochemical systems, e.g., batteries, where intercalation mechanisms, solvent/electrolyte polarisation gradients and interfacial reactions may all be studied^65^. This is particularly in contrast to other low-cost optical techniques, such as scattering/reflection microscopy^32,53^, which are limited to solids and can also be challenging to interpret/make quantitatively (see Supplementary Note 17). Increasing the time-resolution of Raman (imaging) will allow faster scan rates to be explored, and using optically or electronically-gated methods^26,66^ it may even be possible to probe pico- to nanosecond processes such as electron-transfer and ion (de)solvation^67^. Enhancing the spatial resolution of Raman imaging with near-field^68^ or super-resolution^69,70^ methods will further aid in answering of such questions.

## Methods

### Preparation of the working electrodes

α-Li_2_IrO_3_ was synthesized by grinding IrO_2_ and Li_2_CO_3_ (in excess of 5%) according to the literature^71^. IrO_2_ was purchased from Alfa Aesar (Premion®, 99.99% metals basis, Ir 84.5% min) and LiCoO_2_ from Sigma-Aldrich (99.8% trace metals basis). Electrodes were prepared by drop-casting an ink containing oxide catalyst powder on ITO-coated cover slides (400 mm², 0.15 – 0.17 mm thick, Diamond Coatings Ltd) or 0.15 – 0.17 mm thick cover slides with ~ 10 nm of Ti evaporated atop of them (see Supplementary Note 2). The ink was prepared by sonicating 5 mg of catalyst in 970 µL of tetrahydrofuran (anhydrous, ≥ 99.9%, inhibitor-free, Sigma-Aldrich) for 1 h. 30 µl of a Nafion D-520 dispersion (5% w/w in water and 1-propanol, ≥ 1 meq/g exchange, Alfa Aesar) were then added to the dispersion. The as-obtained dispersion was gently shaken and subsequently dropcasted onto the ITO or Ti electrodes.

### Electrochemical setup

An Ag wire (0.5 mm diameter; Sigma) was attached to the conductive cover slide using epoxy glue and conductive copper tape to allow connection to the working electrode. The reference (Ag/AgCl porous frit electrode; redox.me) and counter (Pt wire; 0.5 mm thickness; Sigma) were well separated, and the reference was held just above the surface of the cover slides. Micrometre screw gauges were used to achieve exact positioning. Silicone rectangular or circular shaped wells (Mcmaster Carr) with a predefined area were bonded to the sample such that the active area with electrolyte could be determined. Wells were filled with the given electrolyte (200 μL to ~ 1500 μL depending on the experiment) to ensure all electrodes were wet. A Gamry, Reference 600 potentiostat was used to apply all potentials (see Supplementary Note 18 for further details). The experiments were performed in 1.0 M KOH or LiOH electrolytes (pH ~ 14) at room temperature (21 °C). The Ag/AgCl reference electrode was calibrated against a reversible hydrogen electrode (HydroFlex®, from Gaskatel, Germany) in each electrolyte. Potentials were subsequently converted to the RHE scale. Low ohmic resistance was measured by electrochemical impedance spectroscopy, and the voltage was not iR-corrected.

### Raman microspectroscopy

The microscope was a standard layout of an epi-detected Raman microscope. A pump laser beam (wavelength = 532 nm, Coherent Mira) was spectrally cleaned up by a bandpass filter (FLH05532-4, Thorlabs), and its beam width was expanded to 7.2 mm before entering a home-built inverted microscope. Additional waveplates (half-waveplate and quarter-waveplate for 532 nm, Foctek Photonics) precompensated the ellipticity introduced by the dichroic filter (F38-532_T1, AHF) and also generated circularly polarised light. We used high numerical aperture (NA) oil-immersion objectives (Nikon 60X/1.4NA oil) to ensure high-resolution imaging and increase collection efficiency. The maximum pump power before the objective was ~ 30 mW, a power level that ensured no degradation of samples. The samples were scanned with galvanometric mirrors (Thorlabs). The Raman inelastic backscattered light was collected by the same objective and focused with the microscope tube lens either onto the slit of the conventional spectrometer (Andor, Shamrock 303i, grating 300 l/mm) or a 30 mm line slit (Thorlabs) which is at the entrance of the home-built compressive spectrometer whose specifications are detailed in Sturm et al.^39^. A notch filter blocked residual pump light (NF533-17, Thorlabs) before guiding the signal to the spectrometers. The conventional spectrometer is equipped with a high-sensitivity charge-coupled camera (Andor, iXon 897). All images presented with the compressive spectrometer were taken with integration times/pixel in the 20 to 50 μs range. Imaging was performed on ‘flat’ particles. See Supplementary Note 5 for an extensive discussion of particle selection. Recording of data was performed by a custom Matlab programme with external synchronisation to the potentiostat for control of the potential whilst recording Raman hyperspectra/images. For spectrally resolved data, background subtraction was performed using a modified iterative polynomial smoothing method^72^. In Supplementary Note 17 we discuss the ‘low-cost’ nature of our approach and its comparison to other benchtop characterisation methods.

### Density functional theory (DFT) calculations

DFT is performed with localised Gaussian basis functions and the PBE0 hybrid functional as implemented in the CRYSTAL17 code^73^. The choice of the PBE0 functional for modelling exchange and correlation is done in order to reproduce Raman active measured frequencies. For Iridium (Ir), Hydrogen (H), Lithium (Li) and Potassium (K), we employed a triple-ζ split-valence plus polarisation basis set, while we used a double-ζ split-valence basis set for oxygen (O) atoms. Calculations were made for the K^+^ intercalated phase with a stoichiometry of α-Li_1_K_0.25_IrO_3_.0.5H_2_O, close to the one that was experimentally found of α-Li_1_K_0.3_IrO_3_.0.7H_2_O in our previous work^18^. Calculations for the delithiated phase were performed with the α-Li_0.5_IrO_3_ stoichiometry, which is the end product obtained after full delithiation of the phase when oxidised in a Lithium-ion battery (the fully delithiated α-Li_0_IrO_3_ phase cannot be experimentally obtained). This way, changes in the Raman vibrations upon delithiation can be discussed by comparing the fully lithiated phase with the phase with the highest delithiation state. Experimentally, α-Li_0.5_IrO_3_ was not observed upon OER as the partially delithiated phase α-Li_1_IrO_3_ is found to react prior to the full delithiation. Nevertheless, the exact amount of lithium remaining in the phase is hard to experimentally assess, and the final composition is, very likely, very close to α-Li_0.5_IrO_3_ when holding the potential at high potential for a prolonged period of time. Full details on DFT calculations are given in Supplementary Note 9.

### Bright-field ‘bubble imaging’

Optical imaging of bubbles was performed using a custom upright microscope with a 0.4 numerical aperture, 60 × objective with a protected aperture for imaging through the (1 M) KOH electrolyte. α-Li_2_IrO_3_ samples were prepared on ITO substrates and contacted exactly as for Raman imaging. White light illumination was used, and the reflected light was collected by a complementary metal oxide semiconductor (CMOS) camera (UI-3080CP Rev. 2, IDS). We focus on agglomerates that are relatively isolated from one another and are 2–5 μm in lateral size to be in keeping with our Raman imaging. The spatial resolution in such experiments is ~ 400 nm with a localisation precision derived from the signal-to-noise and is ~ 300 nm. The frame rate of 10 frames per second sets the time resolution of 0.1 s. The potential was scanned at 5 mV/s in experiments. All imaging was performed after the second cycle.

### Scanning electron microscopy (SEM)

SEM images were acquired on a GeminiSEM 360 microscope from ZEISS, using an acceleration voltage of 5 kV.

## Supplementary information

Supplementary Information for Manuscript

Peer Review File

Description of Additional Supplementary Files

Supplementary Video 1

Supplementary Video 2

Supplementary Video 3

Supplementary Video 4

Supplementary Video 5

Supplementary Video 6

Supplementary Video 7

Supplementary Video 8

## Data Availability

The raw data that support the findings within this paper are available at 10.5281/zenodo.13380572.

## Code availability

Analysis and instrumentation codes are available from the corresponding author upon request.
