# Supplementary material for: Concurrent oxygen evolution reaction pathways revealed by high-speed compressive Raman imaging
Source: Nat Commun. 2024 Sep 27;15:8362. doi: 10.1038/s41467-024-52536-7 (PMC11437135; doi:10.1038/s41467-024-52536-7)
Supplement: Supplementary file 1 — Supplementary Information for Manuscript [file 41467_2024_52536_MOESM1_ESM.pdf]

Concurrent oxygen evolution reaction pathways revealed by high-speed compressive Raman imaging

[1,2,3]Raj Pandya✉, [4,5]Florian Dorchies[#], [6,7]Davide Romanin[#], [8]Jean-François Lemineur, [8]Frédéric Kanoufi, [1]Sylvain Gigan, [6]Alex W. Chin, [1]Hilton B. de Aguiar✉ and [4,5,9]Alexis Grimaud✉

[1]Laboratoire Kastler Brossel, ENS-Université PSL, CNRS, Sorbonne Université, Collège de France, 24 rue Lhomond, 75005 Paris, France

[2]Department of Physics, Cavendish Laboratory, University of Cambridge, JJ Thomson Avenue, Cambridge CB3 0HE, United Kingdom

[3]Department of Chemistry, University of Warwick, Coventry, CV4 7AL, United Kingdom

[4]Chimie du Solide et de l'Energie, UMR 8260, Collège de France, Paris, France

[5]Réseau sur le stockage Electrochimique de l'Energie (RS2E), Amiens, France

[6]Sorbonne Université, CNRS, Institut des Nanosciences de Paris, UMR7588, F-75252, Paris, France

[7]Université Paris-Saclay, CNRS, Centre de Nanosciences et de Nanotechnologies, 91120, Palaiseau, France

[8]Université de Paris, ITODYS, CNRS-UMR 7086, 15 rue Jean-Antoine de Baïf, 75013 Paris, France

[9]Department of Chemistry, Boston College, Merkert Chemistry Center, 2609 Beacon St., Chestnut Hill, MA, 02467 USA

✉Correspondence to: raj.pandya@warwick.ac.uk, h.aguiar@lkb.ens.fr, alexis.grimaud@bc.edu

[#]These authors contributed equally

Supplementary information

Table of contents

Supplementary Note 1: Compressive Raman methodology 3
Supplementary Note 2: Substrate effect and degradation 7
Supplementary Note 3: Scanning electron microscopy images of α-Li_2IrO_3 and $LiCoO_2$ 9
Supplementary Note 4: Rationalisation of Raman signals 11
Supplementary Note 5: Identification and influence of agglomerate flatness on substrate 17
Supplementary Note 6: Regions of interest on the anodic scans 23
Supplementary Note 7: Raman spectra over multiple cycles 24
Supplementary Note 8: High-resolution Raman imaging and influence of peak-shifting 26
Supplementary Note 9: Density Functional Theory Calculations 29
Supplementary Note 10: Derivative of the Raman intensity vs time 34
Supplementary Note 11: Operando Raman imaging using LiOH electrolyte and α-Li_2IrO_3 particles and KOH electrolyte and IrO_2 35
Supplementary Note 12: Operando Raman imaging of $LiCoO_2$ in KOH 45
Supplementary Note 13: Operando Raman imaging with KOH electrolyte of α-Li_2IrO_3 at 550 cm^{-1} and over multiple particles 47
Supplementary Note 14: *Operando* gas bubble imaging 50
Supplementary Note 15: Estimation of cation (de)intercalation velocities 55
Supplementary Note 17: Cost comparison between single pixel Raman spectrometers, EMCCD based Raman spectrometers and bright-field microscopes 65
Supplementary Note 18: Additional details on experimental setup 67
Supplementary References 69

Supplementary Note 1: Compressive Raman methodology

The methodology of compressive Raman microspectroscopy has been detailed fully previously[1–4]. Here, we demonstrate the validity of our implementation and provide details of spectral filters used, in particular demonstrating no leakage between species channels. We consider the spectrum of our system as consisting of 4 species with different spectra (despite the fact that only 3 chemical species are present): $\mathbf{S_1}$ = 550 cm^{-1} mode of Li_2IrO_3; $\mathbf{S_2}$ = 640 cm^{-1} mode of Li_2IrO_3; $\mathbf{S_3}$ = Nafion and $\mathbf{S_4}$ = Background. We can write the library spectra as a matrix, **S**. The filters for each of the species $\mathbf{S_1}$ to $\mathbf{S_3}$ will cover each of the Raman bands of the given species, whilst $\mathbf{S_4}$ covers the remainder of the spectrum. We denote the matrix of spectral filters **F** as depicted in **Supplementary Figure 1**. We define a matrix $\mathbf{G} = \mathbf{S}^{T}\,\mathbf{F}$. Within the compressive Raman imaging framework it is key to ensure that these filters, which correspond to patterns on the digital micromirror device (DMD), allow efficient estimation of each of the species. The species proportion $\hat{\mathbf{z}}$ is estimated as $[\mathbf{G}^{T}\mathbf{G}]^{-1}\mathbf{G}^{T}\mathbf{n}$, where the T denotes the transpose and **n** represents the measurements. In the main manuscript, we plot images based on **n**. Given that **G** is close to diagonal, there is little to no species leakage between the measurements **n**. This is better seen in **Supplementary Figure 2** where we demonstrate this to be the case, validating our approach. Therefore, the figures in the main manuscript are measurements of the species themselves.

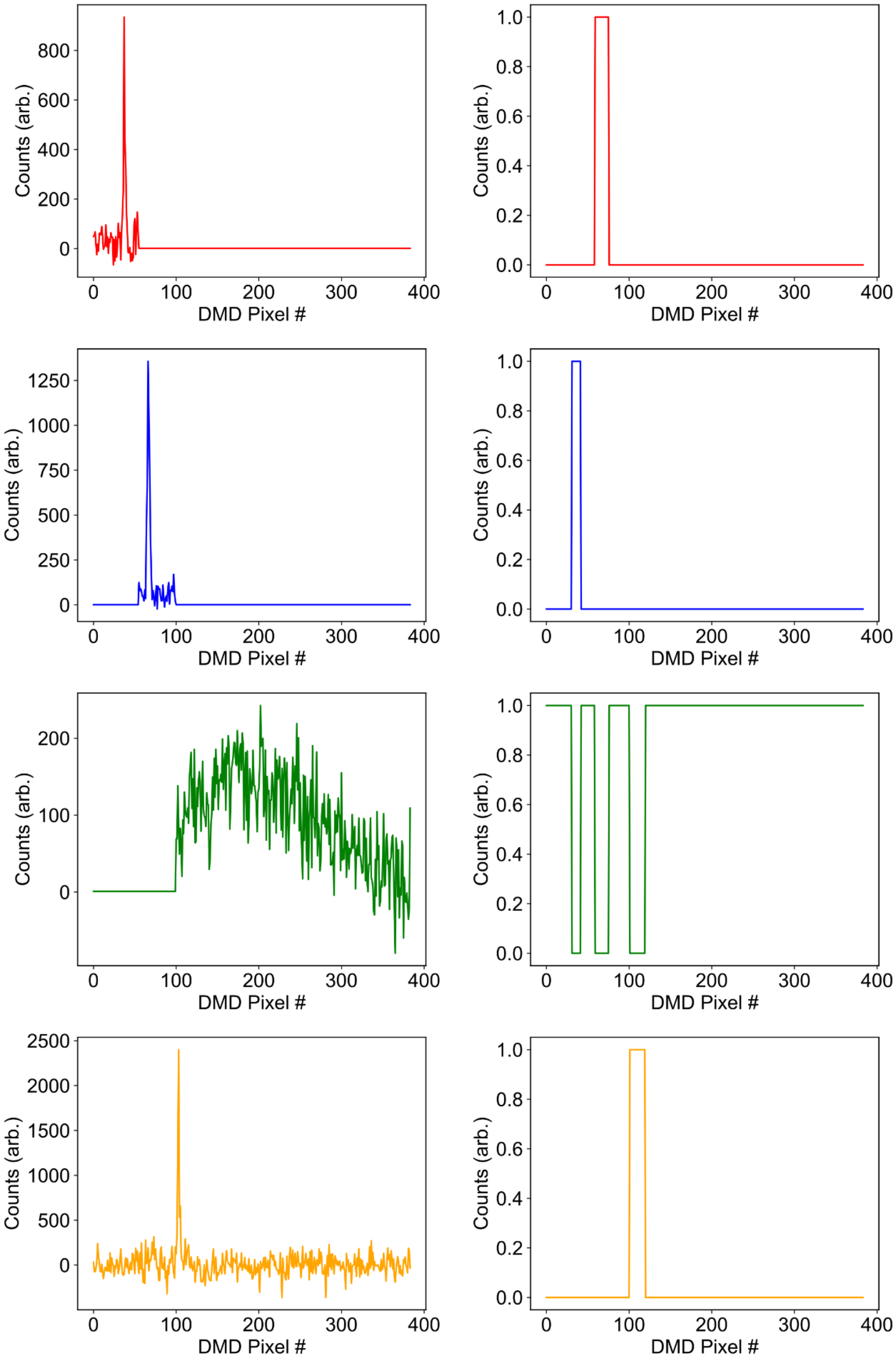

Supplementary Figure 1: Spectra and filters for compressive Raman spectroscopy. Spectra of four species present in spectra of system with associated filters. We note that we consider the two peaks of α-Li_2IrO_3 and background as three separate species as they each have

a different response. We hence also segment the spectra for each component around its band, padding the remainder of the spectrum with zeros.

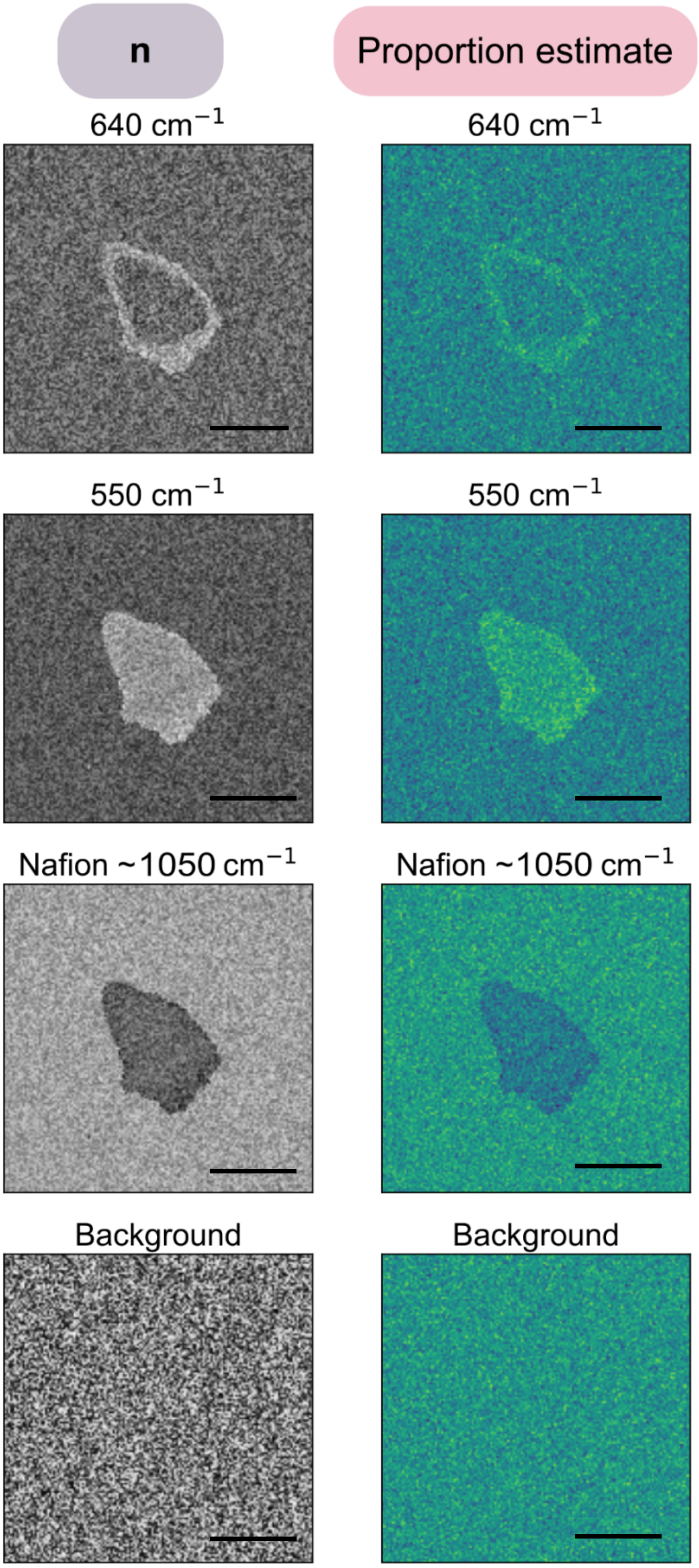

Supplementary Figure 2: Proportion estimation images at various times during electrochemical cycling. Images of system at given timepoints during cycling (left) and associated images obtained from proportion estimation (right). Images appear identical based

on visual inspection indicating no leakage between channels. On left colorbar is black (low counts) to white (high counts). On the right the colorscale is blue (low counts) to yellow (high counts). Scale bar is 5 µm.

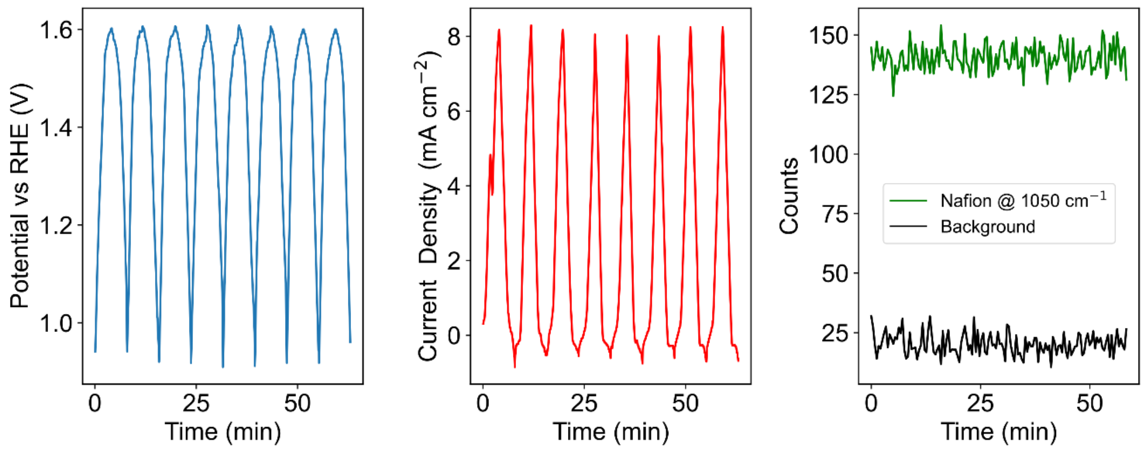

Supplementary Figure 3: Nafion Raman response during cycling. Intensity of Nafion spectral band (away from agglomerate particles) centred around ~1050 cm^{-1} and background. The intensity of both remains constant throughout cycling of the catalyst. The voltage is not iR corrected.

Supplementary Note 2: Substrate effect and degradation

ITO is known to degrade in high (low) pHs at negative (positive) potentials[5]. Under the high pH, positive potential and sub-1 hr exposure times used, we find a change of only 5 to 10% in the substrate transmission over the 60 to 80 mins over which experiments are performed. Nonetheless to ensure that such effects do not influence our results, measurements requiring cycling over 40 mins *e.g.* multiple slow cycles, were also performed on coverslides (0.13 – 0.15 mm thickness) coated in Ti (10 nm).

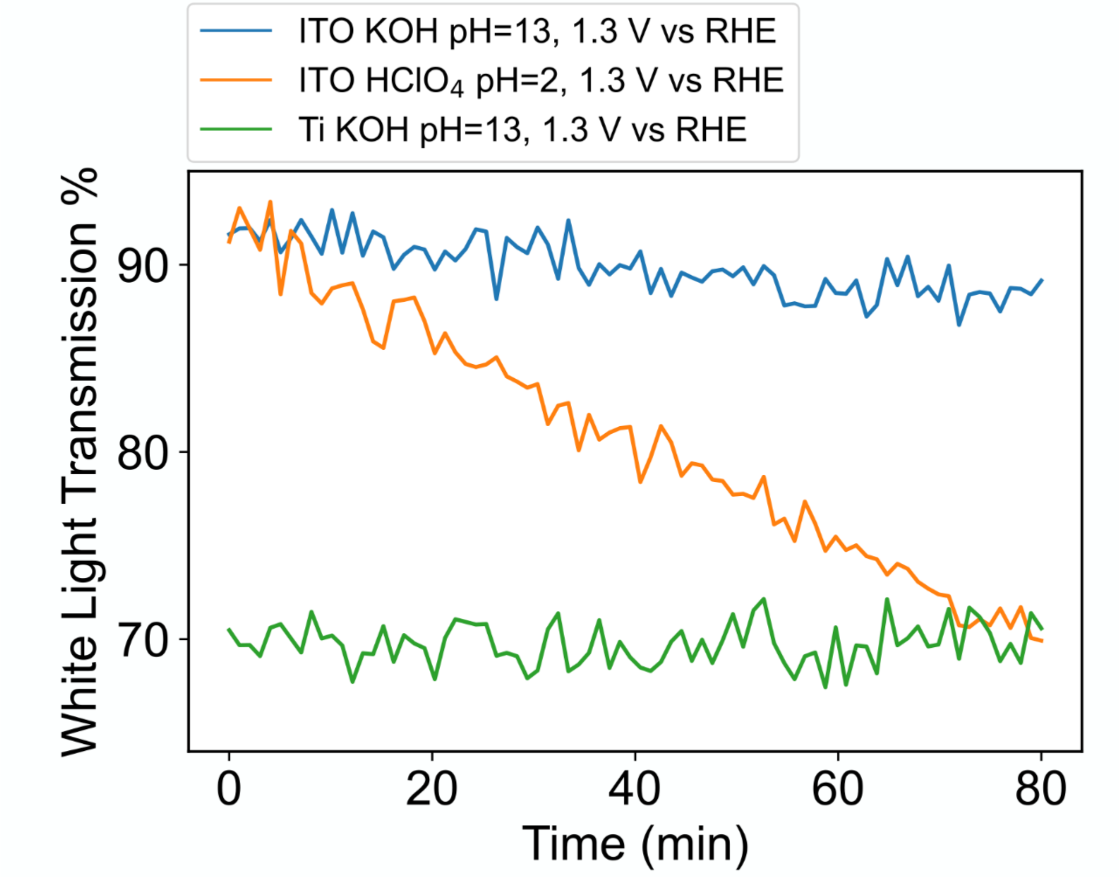

Supplementary Figure 4: Stability of ITO or Ti coated slides during potential holds. Transmission of ITO or Ti coated glass slide under different conditions demonstrating stability under basic conditions at positive potentials but not acidic ones. Potential is ramped to 1.3 V vs RHE before measuring transmission through well of given aqueous electrolyte (2 mm diameter).

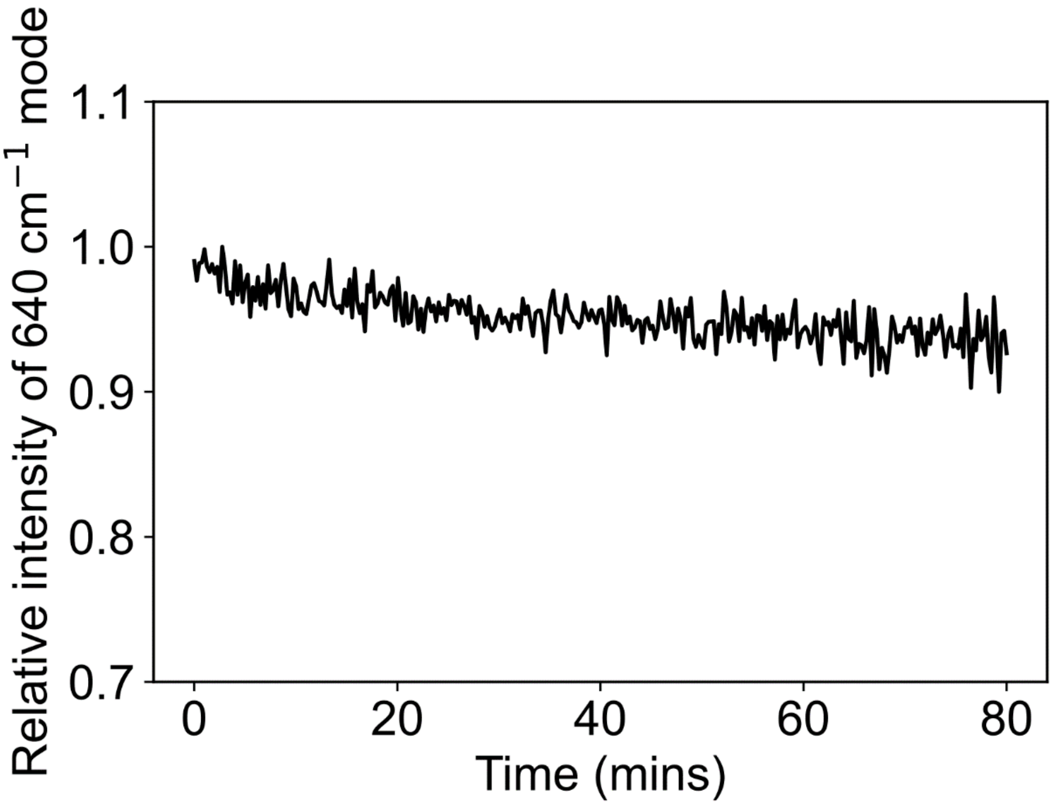

Supplementary Figure 5: Laser stability of α-Li_2IrO_3. Raman mode intensity of ~640 cm^{-1} mode of α-Li_2IrO_3 over 80 mins. Spectra are measured every 25 seconds from a single particle which is continuously illuminated with the 532 nm ~100 mW power before objective. The intensity of the signal decays around ~5% over this period far below that observed in cycling experiments.

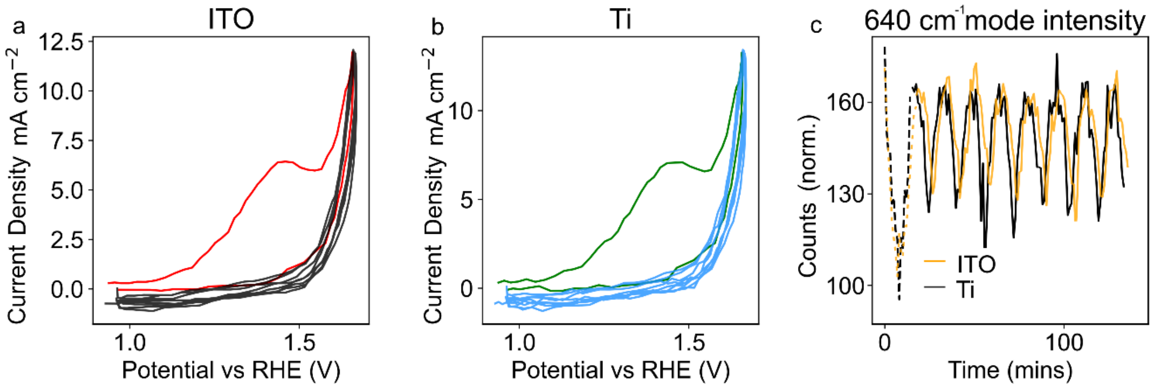

Supplementary Figure 6: Effect of substrate on cyclic voltammogram of α-Li_2IrO_3. a-b. Cyclic voltammograms of α-Li_2IrO_3 deposited on ITO or Ti coated coverslides. An identical electrochemical response is observed between the two. **c.** Intensity of 640 cm^{-1} Raman mode when cycling α-Li_2IrO_3 on Ti and ITO coverslides. The counts are normalised arbitrarily but on both sample slides a similar response is observed. The voltage is not iR corrected.

Supplementary Note 3: Scanning electron microscopy images of α-Li_2IrO_3 and $LiCoO_2$

The α-Li_2IrO_3 we study consists of agglomerate particles that are 2 to 5 μm in lateral size and also 2 to 5 μm in thickness as revealed by the scanning electron microscopy (SEM) images in **Supplementary Figure 7**. The primary particles of the agglomerate can have a quasi-lamellar shape and stacking within the agglomerate but can also be anisotropically stacked and shaped and range in size between 50 and 300 nm based on the SEM images (*e.g.* **Supplementary Figure 7c**).

There are other agglomerate sizes found on the electrode but we focus on those that are 2 to 5 μm in size (i.e. of the smallest sizes that can be reliably resolved with Raman imaging) and composed of over 50 primary particles.

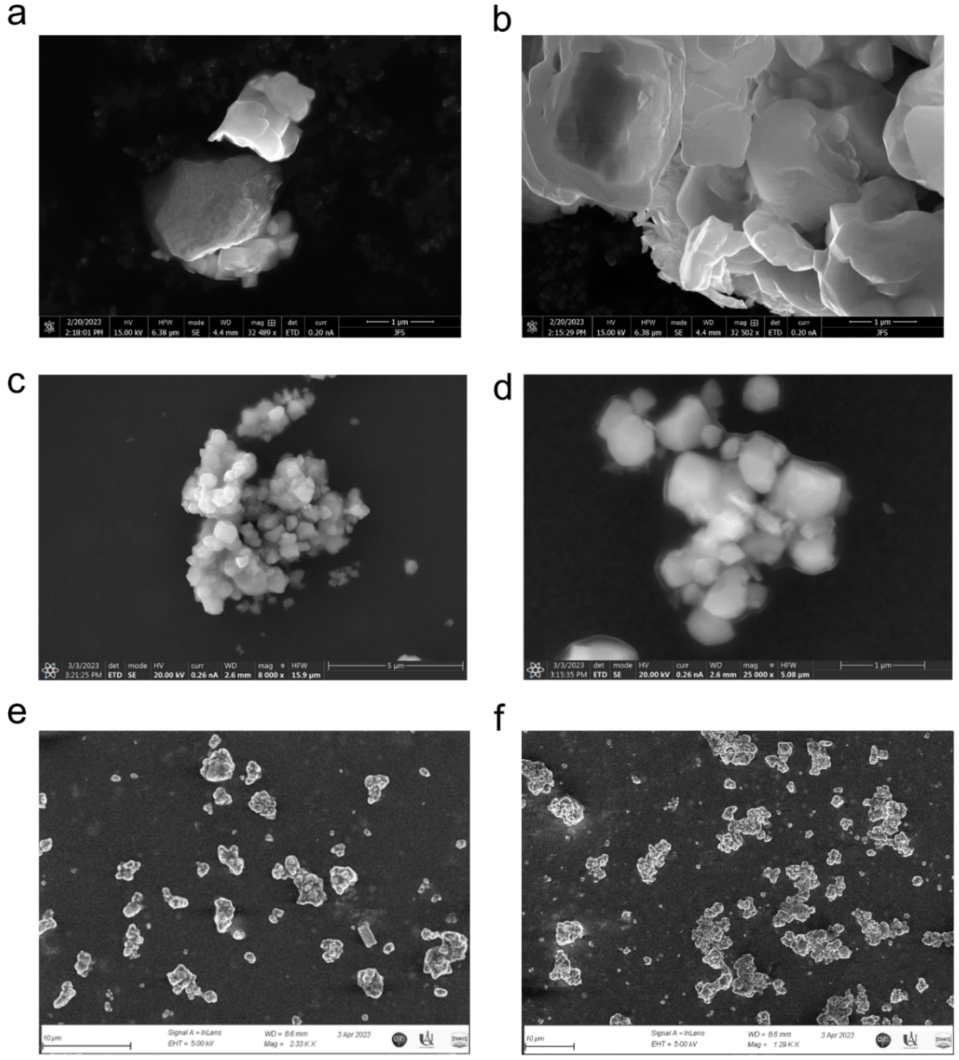

Supplementary Figure 7: Scanning electron microscopy (SEM) images of α-Li_2IrO_3. a-b. Scanning electron microscopy (SEM) images of α-Li_2IrO_3 agglomerates that are ~2 μm in size deposited onto FTO without Nafion. **c-d.** SEM images of α-Li_2IrO_3 agglomerates that are ~2 μm in size deposited onto FTO with Nafion (which presents itself as a blurry shell around the

particle). **e-f.** Zoomed out SEM images of 2 to 5 μm sized α-Li_2IrO_3 agglomerates deposited onto FTO with Nafion.

Supplementary Note 4: Rationalisation of Raman signals

SEM imaging reveals that agglomerates are composed of primary particles (see **Supplementary Figure 7**). The primary particles of the agglomerate can have a quasi-lamellar shape and stacking within the agglomerate, but can also be anisotropically stacked and shaped. Although a range of agglomerate sizes exist we focus on those that are 2 to 5 µm in size (i.e. of the smallest sizes that can be reliably resolved with Raman imaging) and composed of 50 – 100 primary particles.

To reconcile our Raman images with the agglomerate nature of particles we must first consider what volume of the particles our signal arises from, which itself is governed by the balance of the laser attenuation through the sample and the lateral/axial resolution. The lateral resolution $\Delta x = \Delta y$ of an optical microscope (ignoring specific modality) is typically estimated from the formula $\frac{0.61 \cdot \lambda}{NA}$, where NA is the numerical aperture of the optical system (1.4) and λ (532 nm; as the Raman signal scales as $1/\lambda^4$, this wavelength provides a good balance between signal and sample damage from the high-energy laser pump) is the excitation wavelength. For our system this corresponds to $\Delta x = \Delta y$ ~ 230 nm. For the axial resolution a number of formulas exist depending on the degree of confocality of the detection system. In the case of confocal detection the axial point spread function (PSF) $\Delta z = \frac{0.88 \cdot \lambda}{(n^2 - \sqrt{n^2 - NA^2})}$, where n is the refractive index of the oil immersion medium is used (~1.5 here). Using this value, we obtain a value of ~300 nm for the parameters of our system. For widefield detection the axial resolution is defined as $\frac{1.77 \cdot n \cdot \lambda}{NA^2}$ which for our system would be ~770 nm[6]. In our Raman experiments specifically the entrance slit of our spectrometer acts as a (poor) confocal pinhole in one direction so theoretically we might expect to sit somewhere just below the wide-field value.

However, the above formulas do not take account of any spherical aberrations which arise most predominantly from the refractive index (RI) mismatch between the sample and the immersion medium. These spherical aberrations tend to reduce the resolution of a system by distorting the PSF[6]. For the imaging of our agglomerates (refractive index ~2), immersed in aqueous electrolyte (refractive index ~1.33) with an oil immersion objective (immersion oil refractive index ~1.55), calculating the (compound) effect of refractive index mismatch is challenging and no single equation is likely appropriate. We hence estimate the value experimentally by scanning the sample through the focus of our agglomerates and monitoring the magnitude of the Raman signal at 640 cm^{-1} (**Supplementary Figure 8a**). The depth through which moving

the focus the signal remains approximately constant can be taken approximately as the axial signal extent.

We find that the Raman signal does not vary (by more than 15% of its maximum value) within 900 nm of scanning into the agglomerate (**Supplementary Figure 8**) and hence can approximate this as the axial signal extent. To see how this compares with the laser attenuation length, we monitor the intensity of reflected 532 nm light when scanning a thick film of agglomerate through the laser focus. We find the reflected light signal decays to 1/e of its maximum value after 1.32 ± 0.2 μm of scanning into the agglomerate (**Supplementary Figure 8b**)[7]. This value is approximately independent of the intercalation state of the material similar to what is observed in other $LiMO_2$ materials except LCO (see discussion below). This latter value is consistent with the optical skin-depth (attenuation length) as estimated from the absorption coefficient of iridium oxides. Various texts place the absorption coefficient (α) of IrO_x compounds between 0.05×10^5 and 0.15×10^5 cm^{-1} at 532 nm depending on the particle sizes, temperature and crystallinity, giving attenuation lengths (1/α) of 625 nm to 2 μm[8,9]. Using white-light reflectivity measurements we find an approximate α for α-Li_2IrO_3 of ~0.07×10^5 cm^{-1} giving an attenuation length of ~1.4 μm.

It is challenging to ascertain the thickness of the agglomerates from SEM alone (or other surface probes such as AFM). We hence use time-gated optical coherence tomography[10] (TD-OCT; see methods) where we compare the time taken for light reflected from the surface of the agglomerate to arrive at fixed camera position with that taken for light reflected from the substrate adjacent to the particle[11]. From this time difference we can approximate the height of the agglomerates on the substrate. We find that there is some inter-agglomerate variation but measure typical thicknesses of 2 – 5 μm for agglomerates that are 2 – 5 μm in maximum lateral size (**Supplementary Figure 8c,d**). Across a single agglomerate little to no variation in thickness is observed suggesting that the roughness of the agglomerates we measure is within our axial resolution (~0.9 μm).

Taking all the above pieces of information together our results indicate that the overall Raman image we measure is a 2D projection of behaviour at the particle top surface and bulk, with the signal arising from approximately 20% to 35% of the particle depth.

For the lateral dimension it is challenging to exactly ascertain how spherical aberrations influence our resolution, because obtaining a ground truth, e.g. by measuring particle sizes

optically and then via SEM, remains impossible due to an inability to remove the aqueous electrolyte without disturbing agglomerates. However, we can do so by performing Raman imaging across the step edge of a resolution target (immersed in KOH) with assumed infinite sharpness. The width of the derivative of the step across the edge gives an upper bound of the resolution which we find to be ~300 nm.

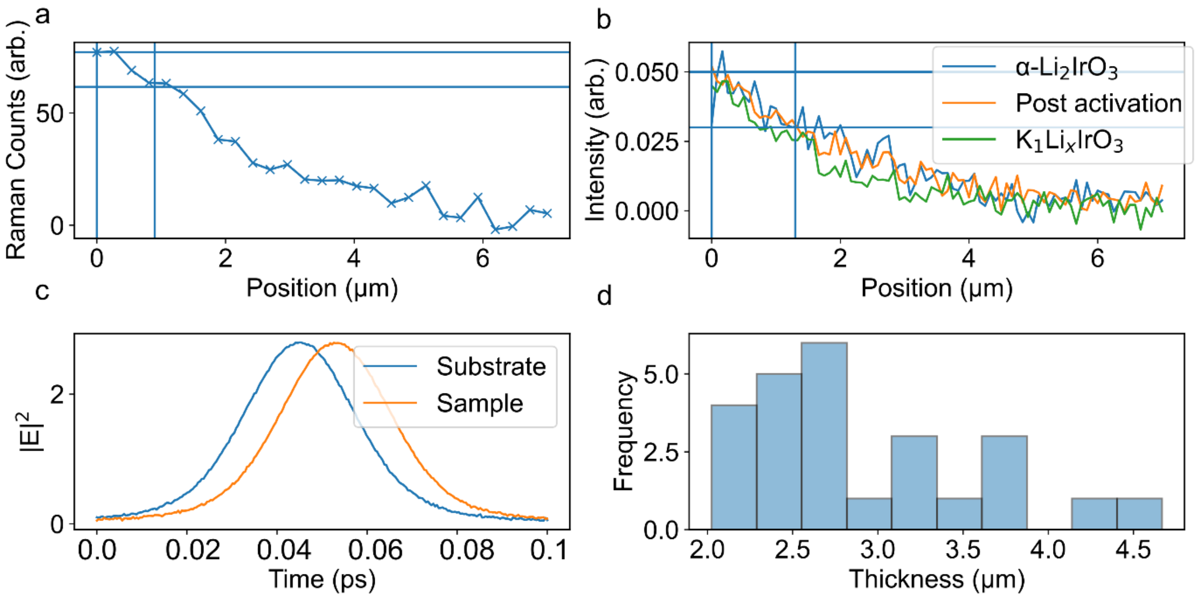

Supplementary Figure 8: Axial resolution of the Raman microscope: a. Raman counts measured by scanning sample through objective focus. The horizontal and vertical lines mark the maximum of the signal and the region where it approximately remains constant (within ~90% of its original value). **b.** Exponential decay of reflected 532 nm light intensity at centre of α-Li_2IrO_3 agglomerate (blue), post activation agglomerate (orange line) and potassium intercalated agglomerate (green). Blue lines mark region where signal remains within of $1/e$ of the initial value. From this the attenuation coefficient of the material can be estimated which is 1.3 μm, i.e. 1.5× the axial resolution. This value is independent of the intercalation state of the material. **c.** Electric field amplitude from time-domain optical coherence tomography (TD-OCT) stack scanning through an agglomerate particle. From the difference in reflectivity peaks the agglomerate thickness can be estimated **d.** Histogram of agglomerate thickness measured using TD-OCT for agglomerates that are 2 to 5 μm in lateral dimension.

Although it is somewhat surprising to observe distinct patterns of intercalation in agglomerates we note that such behaviour is observed and well-documented in the case of ion intercalation in battery materials of similar structure composition. For example, in agglomerates of LFP core-shell patterns of (de)intercalation have been observed using X-Ray microscopy[12] and TEM[13]. Similarly, for NMC agglomerates of various compositions a core-shell pattern of intercalation has been used to explain the (de)lithiation behaviour at the mesoscale[14,15]. Although these patterns and their origin remain debated[16] a competition between surface

reactivity and bulk diffusion remains in the agglomerates and gives rise to such behaviour. We note that at the single particle level, the mechanism of ion (de)intercalation may be the same between agglomerates and single crystals as suggested for NMCs[14–16] or different, as has been described for LFP[13,17].

If we consider for our agglomerates that we have distinct surface and bulk reactivity we can use toy models to rationalise the Raman signals observed (**Supplementary Figure 9**). For example, our observation is that the delithiation follows a core-shell pattern. Assuming a 3D structure, with overall delithiation from the surface through the core, in 2D projection, the: (i) surface will uniformly drop in Raman intensity, (ii) bulk layers will lose Raman intensity first from their edge and then centre. The signal we observe is the sum of these two contributions and hence has an overall core-shell pattern. A similar logic can be applied for the $n \geq 2$ anodic scans: at the very most outer top surfaces changes in signal will be uniform (although not necessarily monotonic with potential), whereas signal from the bulk will change differently at the edges as compared to core, the sum of these two effects gives the overall pattern. We note that quantifying the relative contribution of bulk and surface signal are challenging, the former likely dominates due to its greater volume percentage but we refrain from an overall assignment. An assumption of the outlined toy-model is that agglomerates can be approximated as cuboids with surfaces that are relatively flat (i.e. low curvature). Based on the SEM images presented above the former may be a passable assumption with the flatness discussed below. Nonetheless if agglomerate curvature contributed significantly to the observed signals then one would expect the Raman signal across an agglomerate before cycling to be non-uniform with less signal at the edges. Given this is not observed we do not need to significantly consider curvature.

As discussed in the main text, secondary crystalline catalysts made of primary particles, can have porous gaps between the primary particles which could be penetrable to the electrolyte. However, our SEM imaging (**Supplementary Figure 7**) and Raman signals (**Supplementary Figure 2**) show little evidence of significant porous Nafion binder/gaps, etc between the crystalline primary particles i.e., in the secondary particles. This is our rationalisation for considering the ‘inside’/bulk of the secondary particles as non-electrolyte permeable/non-porous and terming the ‘surface’ that of the secondary particles. Whilst any pores might also result in a ‘core-ring’ like pattern in the Raman imaging (because reaction is first with the external surface that depletes the liquid in the porous structure resulting in a shell

that propagates towards the core[18]), this behaviour would be expected across cycles i.e., no distinct behaviour between first and second anodic scans. This provides further weight that the penetration of any liquid through the limited porosity (which would also create a faster apparent diffusion) can be largely ignored here. We also finally emphasise that in our previous study[25] we showed that α-Li_2IrO_3 has a very low porosity from a Brunauer-Emmett-Teller (BET) analyses which shows a specific surface area of 0.10 m^2/g with no mesoporosity.

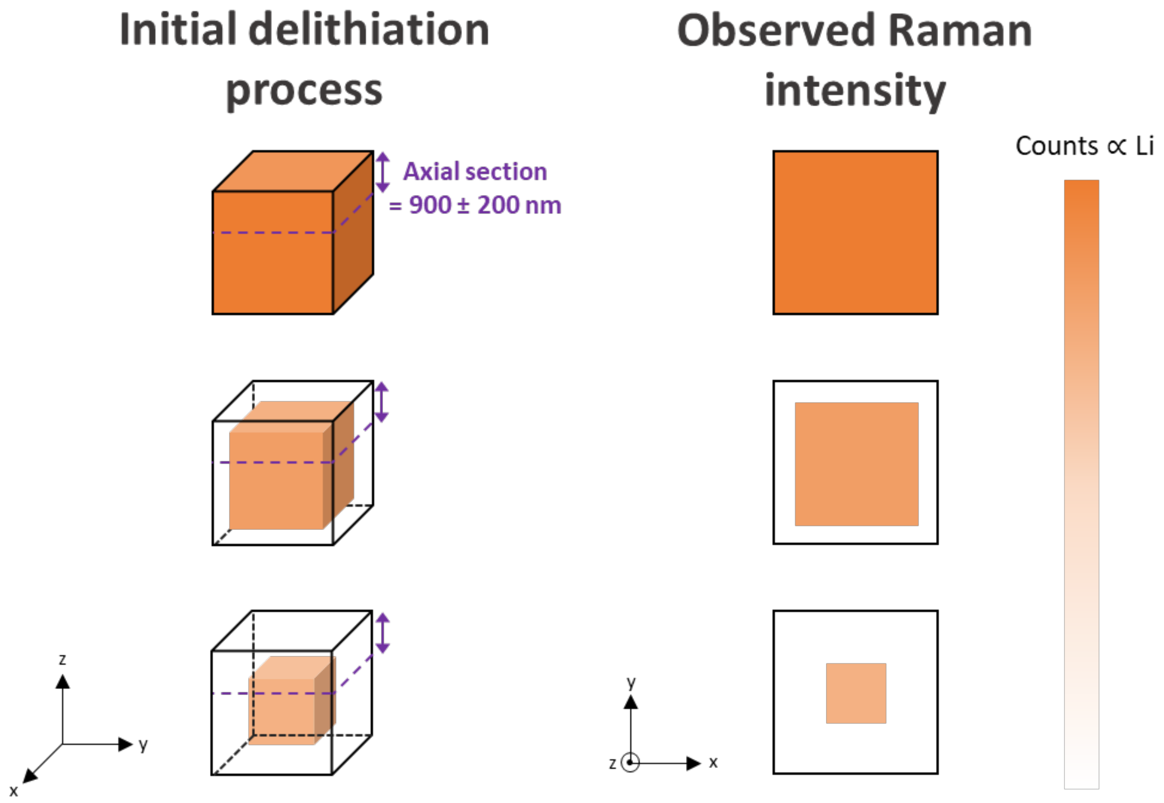

Supplementary Figure 9: Schematic of signals being imaged. Cartoon schematic of the initial delithiation process in a simplified cubic agglomerate, which occurs in 3D (left). The axial section, as calculated above for our setup, is shown in purple. Given that we probe a significant section of the particle bulk (900 ± 200 nm) and not only the top surface, an isotropic 3D delithiation process can give rise to a 2D core-shell pattern when imaging the agglomerates from the top, explaining the observed Raman intensity patterns.

Finally, we also performed imaging of $LiCoO_2$ (LCO) agglomerates (see **supplementary note 12**). LCO has an absorption coefficient higher than that of iridium oxides at 532 nm, which strongly depends on the lithiation state (ranging from 0.1×10^6 cm^{-1} to 0.015×10^6 cm^{-1})[19,20]. This sets the attenuation length inside the material to below that of the axial resolution

(~600 nm). Taking into account the agglomerate thickness this means that the signals we observe for this material arise from less than ~10% of the axial extent.

Supplementary Note 5: Identification and influence of agglomerate flatness on substrate

To determine agglomerates suitable for imaging we first use brightfield imaging. The depth-of-field of a brightfield microscope is calculated as $\frac{\lambda \cdot n}{NA^2} + \frac{(n \cdot p_{sens})}{(M \cdot NA)}$ where, M is the total magnification of the optical system, i.e., objective and imaging lens, and p_{sens} is the pixel sensor size. For our bright-field microscope (taking a centre wavelength of λ of 600 nm) we compute the depth-of-field to be 430 nm. For our Raman imaging the depth-of-field is calculated to be 730 nm. These values are smaller than thickness of the particle underlining that the signal arises from a convolution of both surface and bulk effects as discussed above.

However fully evaluating agglomerate shapes from bright-field/wide-field images is challenging. This is due to optical aberrations and non-uniform illumination in wide-field imaging as well as the covering of particles with Nafion (which is more challenging to discern in the non-chemically specific bright-field image). We hence rely on our Raman images, where the agglomerates can be identified from their spectral signature, to ascribe agglomerate morphologies most accurately. The bright-field images only act as a guide to approximately evaluating agglomerate shape and size.

To determine the flatness, i.e., face perpendicular to the light beam, we use three methods:

1. Before Raman imaging, optical imaging is performed with a Mirau 50× 0.55 *NA* objective. This objective contains a beam splitter that divides the incoming light into a reference and signal path which produce interference fringes. The angle of these fringes relative to the *x-y* imaging plane indicates the degree of object flatness. If fringes are horizontal within the x-y imaging plan the object is optically flat. In this way a flat topped aggregates could be qualitatively assessed. Simply put this objective operates as an optical profilometer.
2. In our time-domain OCT experiments to determine agglomerate thickness (see **Supplementary Figure 8)** we are interfering a reference and signal (reflected light from the sample) to obtain the sample electric field. From this a wrapped phase image can be determined. The orientation of the interference fringes, *i.e.*, how horizontal they are in the *x-y* plane, gives the flatness of the object. For the particles which we measure the phase lines typically deviate ±10° from the horizontal as shown in **Supplementary Figure 10**.

3. Finally, we also measure bright-field images at different z-focal positions and fit the step change between the agglomerate and the substrate at four perpendicular edges. For agglomerates whose top surface is relatively flat the gradient of the step edge is expected to be similar across all 4 edges and change smoothly as the object is moved in and out of the focus. In contrast agglomerates which are not flat would be expected to have edges that are 'blurred' to different extents. The gradient of the step across these edges may also be expected to not change uniformly with focus position. Fitting is performed with a sigmoid function of the form $\frac{L}{(1+e^{-k(x-x_0)})} + b$ where $1/k$ represents the gradient of the step edge. In **Supplementary Figure 11-12** we illustrate the results of such a procedure.

This combined approach allows us to systematically select agglomerates to measure and aids data interpretation.

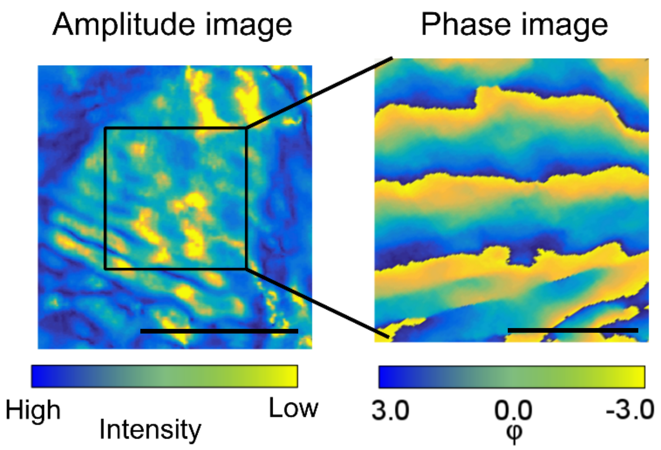

Supplementary Figure 10: Assessing particle 'flatness' using holography. a. Amplitude only image of agglomerate measured (left) and (right) phase image determined from optical coherence tomography of agglomerate showing fringes parallel to horizontal. The interference fringes observed on the amplitude image (left) arise from the use of coherent source for imaging and are not necessarily related to the object flatness. Scale bar is 10 µm on left image and 5 µm on the right image.

For completeness we also include data from agglomerates that do not meet the above flatness criteria (**Supplementary Figure 13)**. These can show similar or different behaviour which could be due both to the lack of flatness or other effects such as the general connectivity of the particles to the electrode. These observations highlight the general challenge of single particle measurements in electrochemistry.

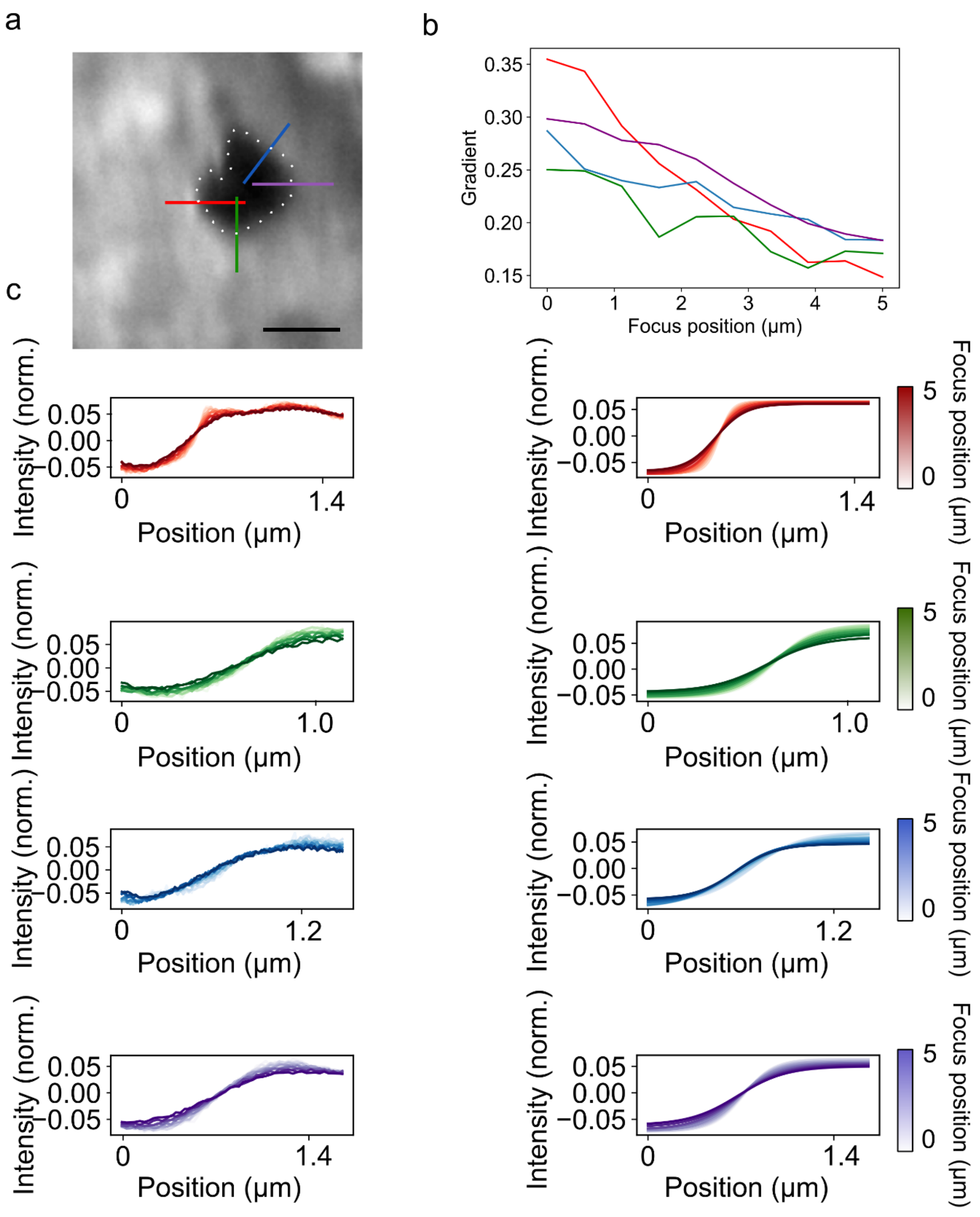

Supplementary Figure 11: z-focus scan across 'flat' α-Li_2IrO_3 particle. a. Bright-field image of 'flat' α-Li_2IrO_3 agglomerate. Coloured lines show pixels along which cut is taken to evaluate sharpness of agglomerate edge in the bright-field image. Scale bar is 1.5 μm. **b.** (Fitted) gradient of sigmoid along edges of agglomerate as a function of focal position. Colours correspond to the edges along which line-cuts are taken in a. **c.** Intensity of bright-field image (left) along pixels shown in a, with corresponding fit of sigmoid function (right). Different lines represent different focus positions 0 (light) to 5 micron (dark).

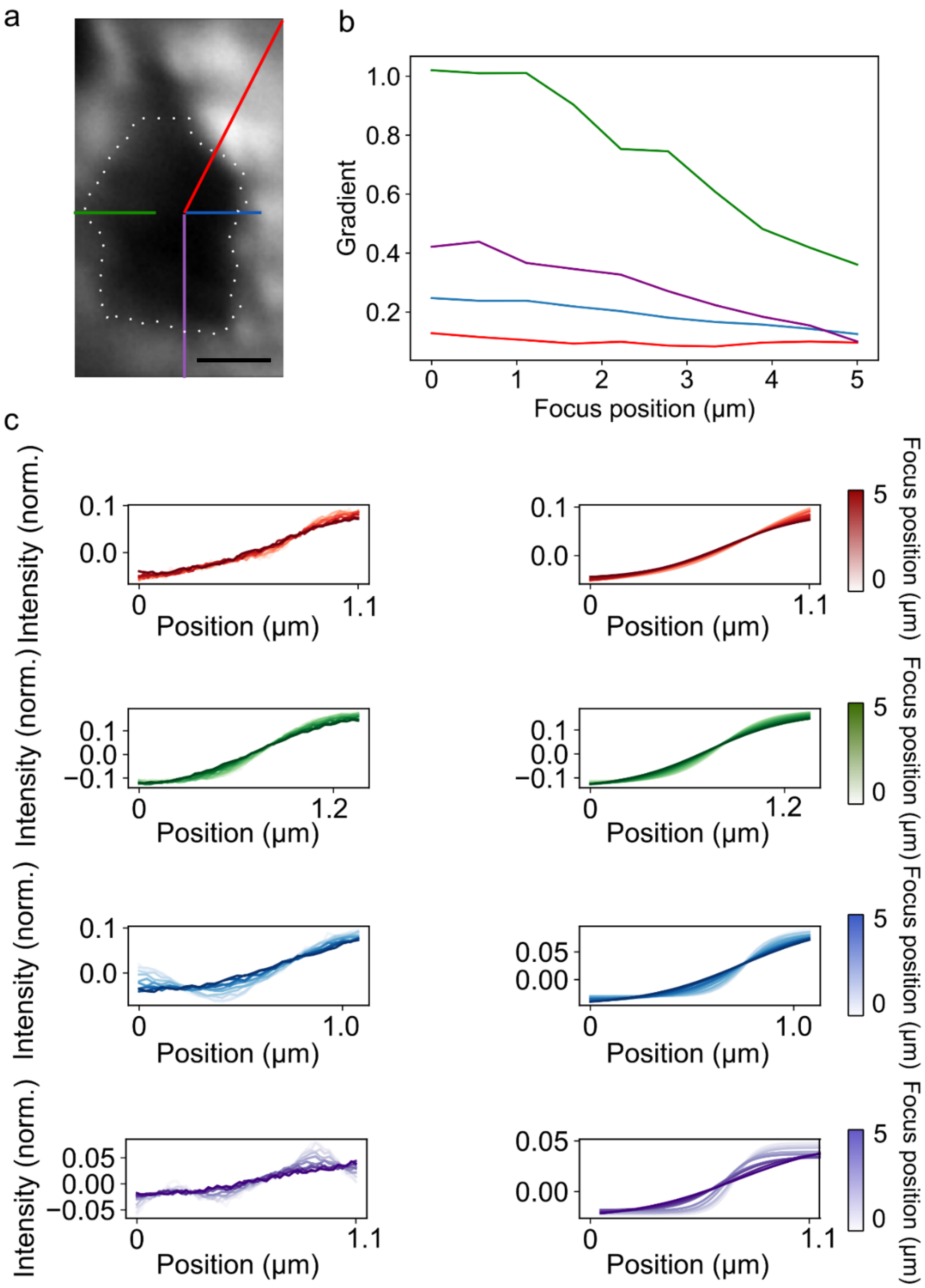

Supplementary Figure 12: z-focus scan across 'non-flat' α-Li_2IrO_3 particle. a. Bright-field image of 'non-flat' α-Li_2IrO_3 agglomerate. The outline of the agglomerate and its distinction from the Nafion/background is achieved using the Raman images which allow us to distinguish species based on different vibrational signatures (see below). Coloured lines show pixels along which cut is taken to evaluate sharpness of agglomerate edge in the bright-field image. A clear blur can be seen on the left-hand side of the image as compared to the right side demonstrating that the agglomerate cannot be fully in focus in a single plane i.e., is not flat. Scale bar is 1 µm. **b.** (Fitted) gradient of sigmoid along edges of agglomerate as a function of focal position. Colours correspond the edges along which line-cuts are taken in a. **c.** Intensity of bright-field image (left) along pixels shown in a, with corresponding fit of sigmoid function (right). Different lines represent different focus positions 0 (light) to 5 micron (dark).

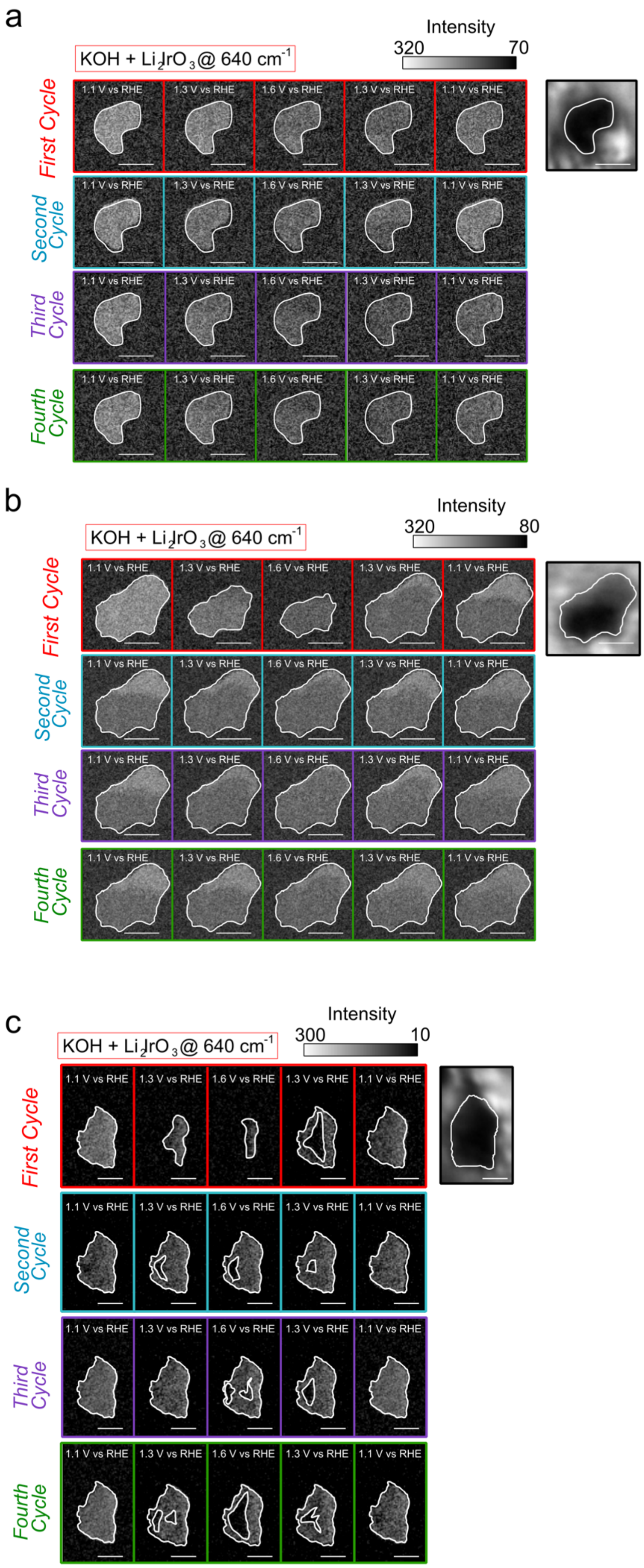
a
Intensity
320
70
KOH + Li2IrO3 @ 640 cm-1
First Cycle
Second Cycle
Third Cycle
Fourth Cycle
1.1 V vs RHE
1.3 V vs RHE
1.6 V vs RHE
1.3 V vs RHE
1.1 V vs RHE
b
Intensity
320
80
KOH + Li2IrO3 @ 640 cm-1
First Cycle
Second Cycle
Third Cycle
Fourth Cycle
1.1 V vs RHE
1.3 V vs RHE
1.6 V vs RHE
1.3 V vs RHE
1.1 V vs RHE
c
Intensity
300
10
KOH + Li2IrO3 @ 640 cm-1
First Cycle
Second Cycle
Third Cycle
Fourth Cycle
1.1 V vs RHE
1.3 V vs RHE
1.6 V vs RHE
1.3 V vs RHE
1.1 V vs RHE

Supplementary Figure 13: Influence of particle 'non-flatness' on Raman imaging. a. Raman imaging of an agglomerate that does not meet (flatness) selection criteria as compared to other agglomerates studied in this work. A bright-field image of the agglomerate is shown on the right and the scale bar in all images is 5 μm. During the first anodic scan there is a dimming of the Raman intensity at 640 cm^{-1} across the agglomerate which increases again during the cathodic scan, in a similar way to agglomerates shown in the main text. The dimming is slightly more pronounced at the agglomerate edges giving rise to the 'shrinking-core' patterns previously discussed. In cycles 2 – 4 the intensity of the Raman image drops during the cathodic scan and increases during the anodic scan as observed for other agglomerates. The decrease in intensity however is non-uniform across the agglomerate with the central top-right hand part of the agglomerate showing a greater dimming as compared to the rest. The overall magnitude of the dimming is small compared to other agglomerates examined in this work, but the spatial changes in Raman intensity are qualitatively similar to other agglomerates *i.e.*, centre of agglomerate dimming with respect to edge. Such behaviour may be a consequence of the agglomerate not lying flat or having a different electrochemical activity. **b.** Raman imaging of a second agglomerate that does not meet (flatness) selection criteria as compared to other agglomerates studied in this work. A bright-field image of the agglomerate is shown on the right and the scale bar in all images is 5 μm. During the anodic scan a shrinking-core type behaviour is observed, with the Raman intensity returning across the agglomerate during the cathodic scan. Interestingly the intensity does not return uniformly across the agglomerate with the top-right of the agglomerate having a distinctly higher intensity than the rest. In the subsequent cycles there is a dimming of the agglomerate during the anodic scan and brightening in the cathodic scan. This behaviour occurs from the centre of the agglomerate to the edges (as was observed for other agglomerates) but only occurs in the top right side of the agglomerate. **c.** Raman imaging of a third agglomerate that does not meet (flatness) selection criteria as compared to other agglomerates studied in this work. A bright-field image of the agglomerate is shown on the right and the scale bar in all images is 5 μm. Delithiation of the agglomerate (during the first anodic scan) occurs from the agglomerate edges and proceeds towards the centre as observed for other agglomerates. In the subsequent cathodic scan, a front of Raman intensity proceeds back from the edges to the centre. This is in-line with previous behaviour observed for potassium intercalation into agglomerates. In the subsequent anodic and cathodic scans of the agglomerate a 'ring' like pattern emerges in the Raman intensity across the agglomerates. This is similar to the behaviour displayed for the agglomerate in the main text although in this case the drop in Raman intensity at the agglomerate centre is somewhat asymmetric across cycles and the agglomerate. Furthermore, the behaviour is confined to the left-side of the agglomerate. The discrepancy between the Raman image (at 1.1 V vs RHE) and the bright-field image for this agglomerate highlights the challenge in using the bright-field images to fully characterise the morphology of agglomerates.

Supplementary Note 6: Regions of interest on the anodic scans

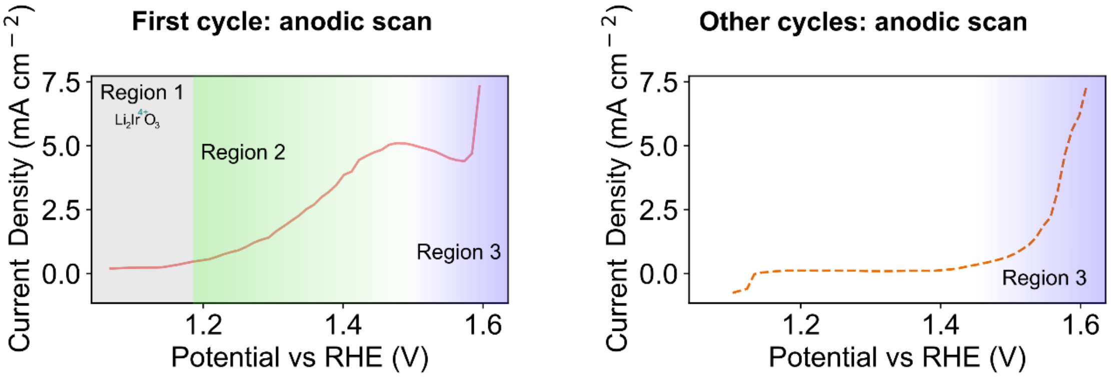

Supplementary Figure 14: Summary of processes occurring at different potentials during the anodic scan of α-Li_2IrO_3. Schematic showing processes occurring at different potential ranges during the anodic scans (which are recorded in 1 M KOH (aq) electrolyte). The voltage is not iR corrected.

In Region 1 of **Supplementary Figure 14** the catalyst is in its initial state (α-Li_2IrO_3), i.e., when no Faradaic processes have occurred. Region 2 corresponds to the initial irreversible oxidation of α-Li_2IrO_3 into α-Li_1IrO_3. Regions 1 and 2 are unique to the very first cycle. Region 3 corresponds to the OER potential range, with α-Li_1IrO_3 being the active phase in this region. As the potential is dropped during the cathodic scan, the chemically-driven intercalation of hydrated potassium occurs, yielding the phase denoted as α-$Li_1K_xIrO_3$ in the main text. As demonstrated in our previous study, on (≥ 2) anodic scans, K^+ deintercalation is driven at ≈1.4 V vs RHE giving again the OER active phase α-Li_1IrO_3. In the anodic scan in the OER region, for 1 M KOH (aq) electrolytes, the two concurrent charge compensation pathways (**steps 2** and **2’** discussed in the main text) are thereafter occurring, their extent/interplay being dependent on the overpotential/current density and at the core of our present study.

Supplementary Note 7: Raman spectra over multiple cycles

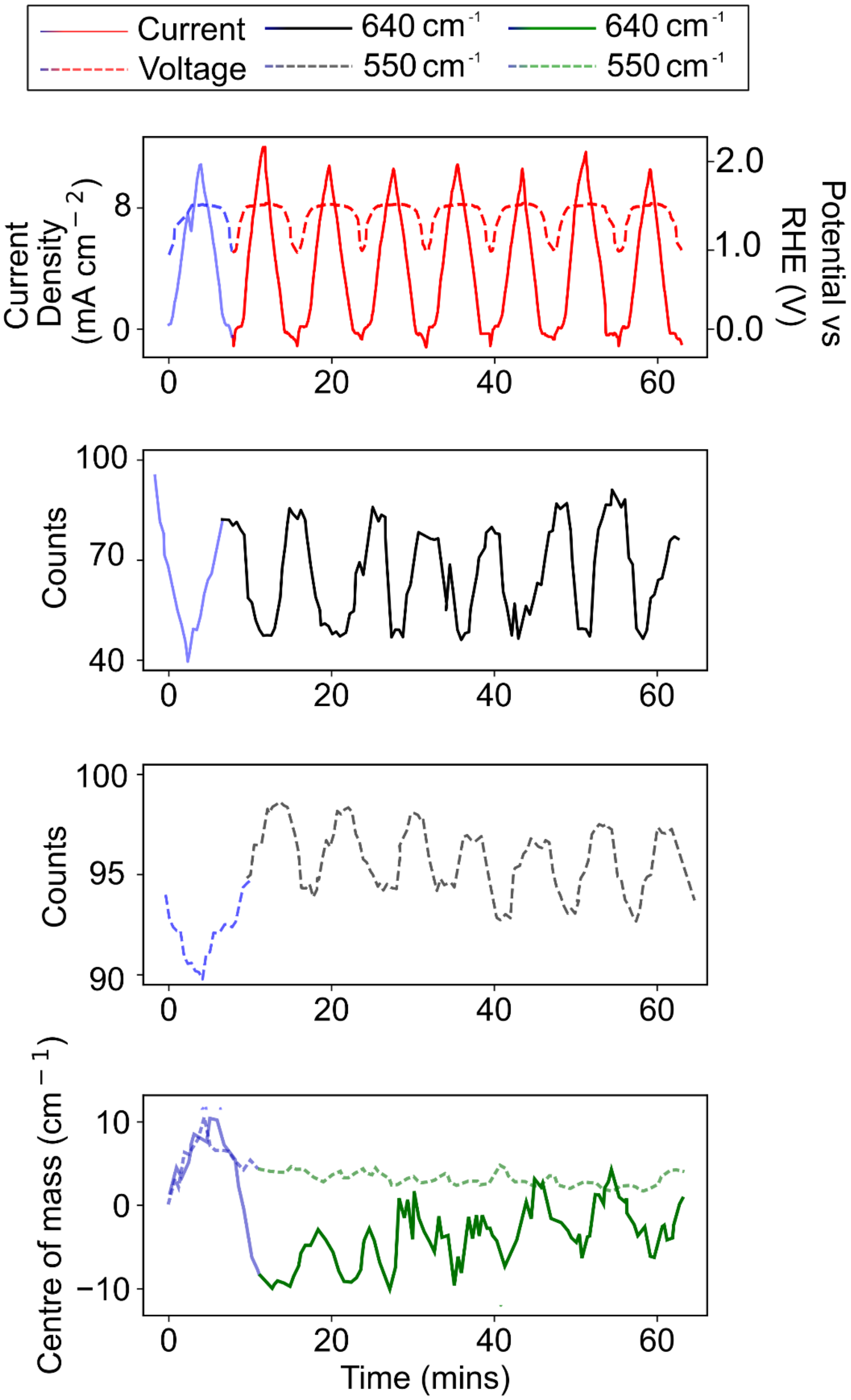

Supplementary Figure 15: Mode intensity and centre frequency evolution during electrochemical cycling of α-Li_2IrO_3. Current (left axis)-voltage (right axis) profile and associated intensity of modes and centre-of-mass over 8 cycles of α-Li_2IrO_3. First cycle (activation) is highlighted in blue. The voltage is not iR corrected.

The broad spectral window of collection we use (300 – 2200 cm^{-1}) for single shot Raman measurements results in the sacrifice of spectral resolution to ~12 cm^{-1}. Hence, rather than report absolute peak positions we report the peak centre-of-mass. The weighted estimate of the peak centre $x_{CoM} = \frac{\sum x_i I_i}{\sum I_i}$ where x_i and I_i are the position and intensities of the ith data point, provides a more accurate value for the peak position in our case.

Supplementary Note 8: High-resolution Raman imaging and influence of peak-shifting

We perform spectral only measurements with a higher resolution (1200 lines per mm grating) focussing on the 640 cm^{-1} mode in both LiOH and KOH (**Supplementary Figure 16**). These measurements show that in KOH the 640 cm^{-1} mode initially shifts to higher frequencies on delithiation (post activation spectrum), followed by a shift to lower frequencies on potassium intercalation. In contrast in LiOH the initial shift to higher frequencies on delithiation is also observed but following a cathodic scan the centre of the spectrum does not shift. These observations demonstrate that the Raman spectra are very sensitive to Li^+ and K^+ intercalation. In our Raman imaging we typically integrate across the entire spectral bandwidth >50 cm^{-1} and it is the distinct spatial behaviour of the intensity fronts in LiOH and KOH that allows us to distinguish between behaviour in the two electrolytes. To see if we can capture the effect of this spectral shift in our images we performed an experiment where we set up three spectral filters: one at the red edge (RF) of the 640 cm^{-1} peak one at the blue edge (BF) and a third filter to capture the background/any other effects. We perform imaging on two particles in KOH and LiOH monitoring the first anodic and cathodic scans. If our above assertions hold then noting the changes in intensity during the anodic/cathodic scans:

1. In KOH during the anodic scan, intensity in the BF channel should drop more relative to the RF channel as in the former there will also be a loss of intensity due to peak shifts.
2. In KOH during the cathodic scan, intensity in the BF channel should grow faster relative to the RF channel as in the latter there is a loss of intensity due to peak shifts.
3. In LiOH during the anodic scan intensity in the BF channel should drop more relative to the RF channel as in the former there will also be a loss of intensity due to peak shifts.
4. In LiOH during the cathodic scan any small intensity changes might be expected to be larger in the RF as compared to the BF channels.

Examining the results of such experiments as shown in **Supplementary Figure 17-18** suggests that our observations do indeed match with the above hypotheses. In summary, the different spectral shifts observed during the anodic/cathodic scans and the different spatial patterns of intensity observed indicate that Raman is indeed sensitive enough to distinguish between behaviours in KOH and LiOH.

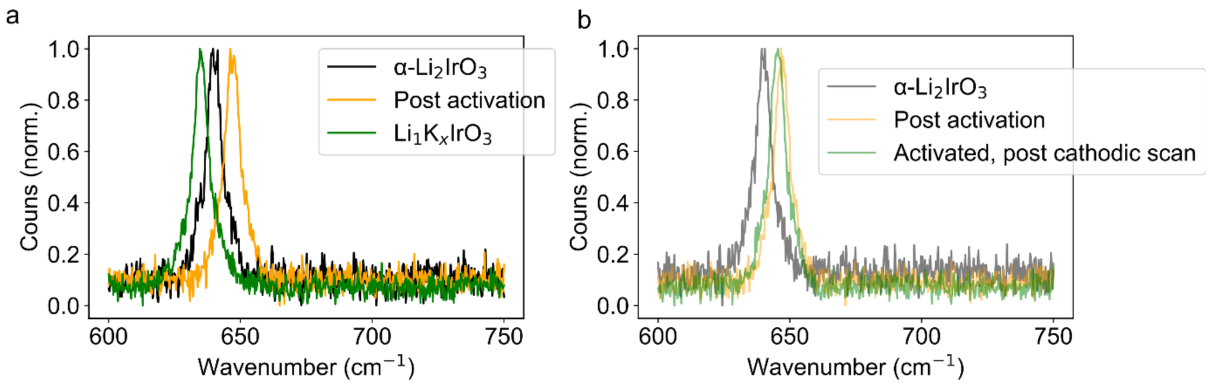

Supplementary Figure 16: High resolution Raman spectra during electrochemical cycling of α-Li_2IrO_3. a-b. Normalised high-resolution (~1 cm^{-1}) Raman spectra centred around 640 cm^{-1} measured in KOH (a) and LiOH (b). Spectra show the α-Li_2IrO_3 phase, phase following delithiation (post activation) and phase following the activation and a cathodic scan.

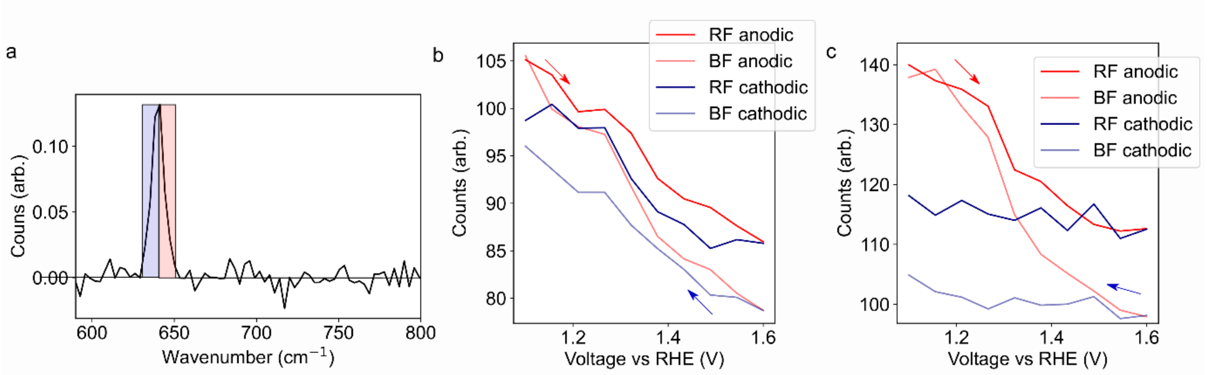

Supplementary Figure 17: Influence of spectral shifts on Raman intensity changes during electrochemical cycling of α-Li_2IrO_3. a. Raman spectrum of α-Li_2IrO_3 with two spectral filters defined at blue and red-spectral edge. **b-c.** Change in intensity of 640 cm^{-1} Raman mode calculated at red-edge (RF) and blue-edge (BF) of spectrum during cathodic and anodic scans in KOH (b) and LiOH (c). Intensities are derived from images in **Supplementary Figure 18**. The voltage is not iR corrected.

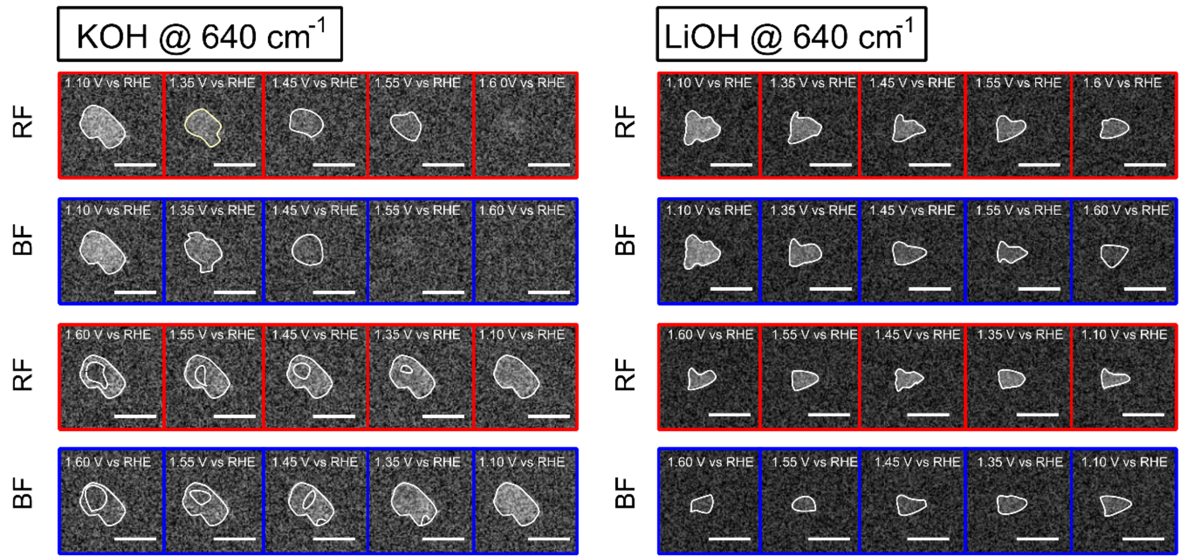

Supplementary Figure 18: Influence of spectral shifts on Raman images during electrochemical cycling of α-Li_2IrO_3. Compressive Raman images of α-Li_2IrO_3 at 640 cm^{-1} in KOH (left) and LiOH (right), taken at the blue-edge (BF) and red-edge (RF) as defined in **Supplementary Note 8**. Scale bar is 4 μm in both images.

Supplementary Note 9: Density Functional Theory Calculations

Density functional theory (DFT) is performed with localized Gaussian basis functions and the PBE0 hybrid functional as implemented in the CRYSTAL17 code[21,22]. The choice of the PBE0 functional for modelling exchange and correlation is done in order to reproduce Raman active measured frequencies. For Iridium (Ir) [23], Hydrogen (H), Lithium (Li) and Potassium (K) [24] we employed a triple-ζ split-valence plus polarization basis set, while we used a double-ζ split-valence basis set for oxygen (O) atoms[23]. Real space integration tolerances of 7-7-7-15-30 for α-$Li_{0.5}IrO_3$, 8-8-8-16-32 for α-Li_2IrO_3 and 10-10-10-20-40 for the K^+ intercalated system were used. Calculations were made for the K^+ intercalated phase with a stoichiometry of α-$Li_1K_{0.25}IrO_3.0.5H_2O$, close to the one that was experimentally found of α-$Li_1K_{0.3}IrO_3.0.7H_2O$ in our previous work[25]. Calculations for the delithiated phase were performed with the α-$Li_{0.5}IrO_3$ stoichiometry, which is the end product obtained after full delithiation of the phase when oxidized in a Lithium-ion battery (the fully delithiated α-Li_0IrO_3 phase cannot be experimentally obtained). This way, changes in the Raman vibrations upon delithiation can be discussed by comparing the fully lithiated phase with the phase with the highest delithiation state. Experimentally, α-$Li_{0.5}IrO_3$ was not observed upon OER as the partially delithiated phase α-Li_1IrO_3 is found to react prior to the full delithiation. Nevertheless, the exact amount of lithium remaining in the phase is hard to experimentally assess and the final composition is, very likely, very close to α-$Li_{0.5}IrO_3$ when holding the potential at high potential for a prolonged period of time.

We set the energy tolerance for the self-consistent cycle to 10^{-10} Ha. The Brillouin zone was sampled with a grid of 8×8×8 *k*-points for α-$Li_{0.5}IrO_3$ and the potassium intercalated system and 10×10×10 k-points for α-Li_2IrO_3, with a Fermi-Dirac smearing of 0.0008 Ha in all cases. We have only considered a paramagnetic groundstate since experimental measurements are performed at a sufficiently high temperature that magnetic order cannot be stabilized. We fixed the lattice parameters to the experimental values and relaxed the internal coordinates.

Since Ir can lead to spin-orbit interaction (SOC), we checked its relevance on the Raman active frequencies in α-$Li_{0.5}IrO_3$ and α-Li_2IrO_3 at the PBE level via plane-waves and Quantum ESPRESSO[26,27]. This also allowed us to check the quality of the Gaussian basis sets we have chosen. For this scope we have employed a ultrasoft pseudopotential[28,29] for Li and Ir and a projector augmented-wave (PAW) pseudopotential[28,29] for O, with 75 Ry and 600 Ry cut-off

for the energy and density respectively. The energy convergence for the self-consistent cycle is set to 10^{-10} Ry. The irreducible Brillouin zone has been sampled with 150 *k*-points and a Methfessel-Paxton smearing of 0.008 Ry (0.004 Ry when considering spin-orbit coupling) in the case of α-$Li_{0.5}IrO_3$. On the other hand, the irreducible Brillouin zone has been sampled with 282 k-points and a Methfessel-Paxton smearing of 0.0008 Ry (with and without SOC) in the case of α-Li_2IrO_3.

QE – no SOC [cm^{-1}]	QE – SOC [cm^{-1}]	CRY – no SOC [cm^{-1}]
170.0	157.0	160.8
180.6	188.7	176.7
183.4	192.2	179.8
285.2	283.1	277.1
343.1	341.7	330.5
533.3	524.1	536.1
541.8	526.5	549.5
544.9	558.1	552.4
566.9	559.5	565.9
584.5	588.8	573.4

Supplementary Table 1: Raman mode frequencies obtained with PBE functional and no spin orbit coupling for α-Li_2IrO_3. Comparison of the Raman active frequencies at the PBE level with and without spin-orbit coupling (SOC) as obtained via Quantum ESPRESSO (QE) and via CRYSTAL17 (CRY) for α-$Li_{0.5}IrO_3$.

QE – no SOC [cm^{-1}]	QE – SOC [cm^{-1}]	CRY – no SOC [cm^{-1}]
-130.9	105.2	-110.3
82.6	149.3	67.0
154.6	203.1	154.8
252.8	249.7	280.3
274.1	270.8	294.2
280.9	273.1	316.0
308.4	342.7	370.0
321.4	347.4	382.9
374.6	376.6	387.8
431.3	444.1	462.2
449.2	485.1	475.5
456.8	496.6	476.9
515.6	510.0	531.0
519.5	520.5	533.3
589.6	598.4	586.7

Supplementary Table 2: Raman mode frequencies obtained with PBE functional and spin orbit coupling included for α-Li_2IrO_3. Comparison of the Raman active frequencies at the PBE level with and without spin-orbit coupling (SOC) as obtained via Quantum ESPRESSO (QE) and via CRYSTAL17 (CRY) for α-Li_2IrO_3.

In **Supplementary Table 1** and **Table 2** we report the comparisons of Raman frequencies for α-$Li_{0.5}IrO_2$ and α-Li_2IrO_3, respectively: we can see that spin-orbit coupling has little to no effect on the Raman active frequencies, with the highest effect happening in the low-frequency part of the spectrum. We would also like to stress that the choice of the PBE0 functional removes the vibrational instability appearing at the PBE level without the spin-orbit interaction, as it happens when taking into account SOC.

For $LiK_{0.25}IrO_3.0.5H_2O$ the mode frequencies are indicated in **Supplementary Table 3** below.

Mode number	**Frequency (cm^{-1})**
01	42.16
02	55.38
03	68.25
04	128.97
05	162.37
06	172.39
07	204.39
08	231.09
10	248.82
11	284.95
12	289.34
13	302.60
14	318.11
15	296.64
16	514.07
17	518.18
18	550.98
19	562.51
20	567.70
21	581.57
22	592.89
23	622.44
24	630.19
25	634.47
26	641.27
27	673.97
28	692.24
29	706.41
30	712.67
31	1618.32
32	3585.88
33	3684.67

Supplementary Table 3: Raman mode frequencies of $LiK_{0.25}IrO_3.0.5H_2O$ with PBE functional and no spin orbit coupling included. Table summarising frequency of Raman active modes of $LiK_{0.25}IrO_3.0.5H_2O$. All modes are of A_g symmetry. Calculations are computed using the PBE0 functional (no spin-orbit coupling inclusion).

It is important to note that in studying catalytic phenomena, we are often dealing with open-shell systems (as in our case with K^+) which can influence the accuracy of density functional theory (DFT)[30]. More specifically, in such multireference (MR) systems there is more than one Slater determinant or configuration state function (CSF). This means obtaining quantitative information on physical properties is not possible due to the inherent single-determinant nature of the commonly employed Kohn-Sham formulation of DFT[31,32]. More pertinently, since our systems are metallic from the viewpoint of DFT analysis, it is impossible to obtain mode intensities[33].

Multi-configurational computational techniques from theoretical chemistry, such as coupled cluster (CCSD(T)[34]) could be more suitable for obtaining quantitative predictions in MR systems. However, for such a system with a large unit cell as ours these techniques are prohibitively expensive from a computational resources point-of-view. Moreover, the modelling of water encapsulation via DFT is hard and extremely dependent on the exchange-correlation functional[35].

Given that all we seek from the DFT is qualitative picture of the types of atomic motions involved that could give rise to the stretches we observe and any trends due to K^+ intercalation (rather than using the DFT to interpret precise changes in mode intensity), we take the above treatment.

Our results are also fully supported by symmetry arguments. For the two different symmetries of α-Li_2IrO3 (and $Li_{0.5}IrO_3$) which is in space group #12 (C2/m with monoclinic symmetry) the two active Raman modes are of A_{1g} and E_g symmetry. On potassium intercalation to form $Li_1K_xIrO_3.yH_2O$ these two symmetries remain the main Raman active modes but with different contributions (this can be tested by examination of symmetry tables). It is well acknowledged that for layered materials of these symmetries the band at greater wave number is A_{1g}-like with stretching along the *c*-axis, so consistent with elongation following the intercalation while the band at lower number is E_g-like, so in the *ab* plane[36,37].

The other origin of the change in intensity of the Raman signals could be the 'skin-effect'. This is where the conductivity of a material changes rapidly with (de)lithiation resulting in a change in laser penetration depth and hence signal intensity. An example of this occurring is in specifically the (de)lithiation of LCO. However, this behaviour tends to be the exception than a rule in layered oxides. To confirm this is not occurring in α-Li_2IrO_3 we measure the laser penetration depth for the pristine, activated and potassium intercalated phase of the material. In all three cases we find similar values suggesting changes in Raman intensity do indeed arise from structural changes (**Supplementary Figure 8**).

Supplementary Note 10: Derivative of the Raman intensity vs time

Supplementary Figure 19 demonstrates a plot of the derivative in counts versus voltage for the 640 cm^{-1} mode (from Figure 2c of the main text). This demonstrates both that the 640 cm^{-1} mode counts begin to change only after ~1.3 to 1.4 V and the clear differences between the first and second cycles.

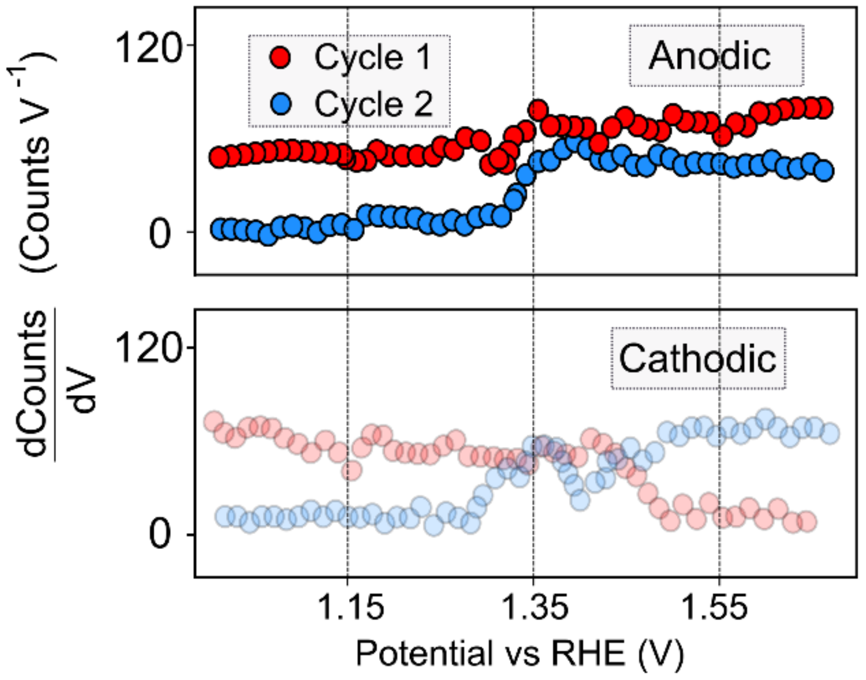

Supplementary Figure 19: **Differential of Raman intensity and potential changes.** Differential of counts *versus* voltage for 640 cm^{-1} mode shown in Figure 2c of main text. The voltage is not iR corrected.

Supplementary Note 11: Operando Raman imaging using LiOH electrolyte and α-Li_2IrO_3 particles and KOH electrolyte and IrO_2

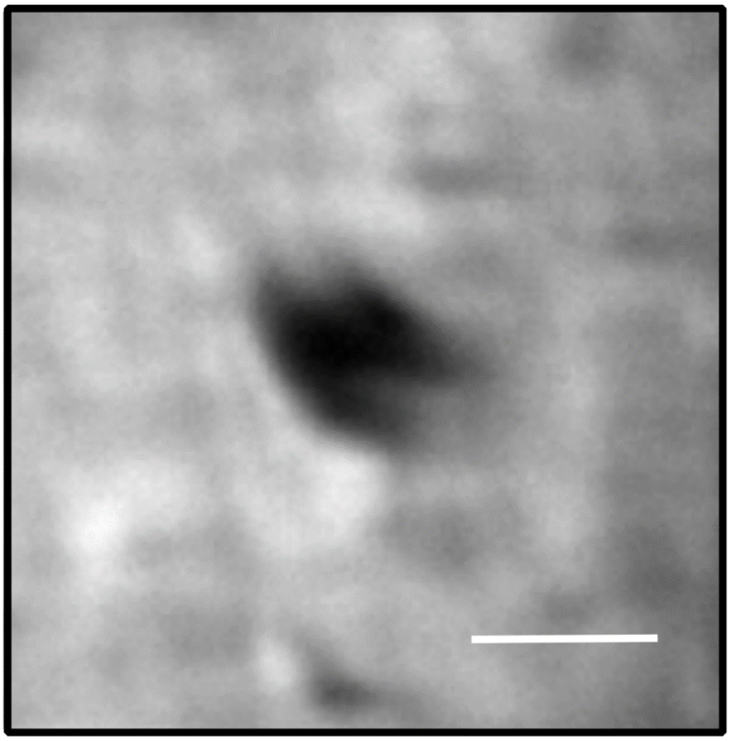

Supplementary Figure 20: Image of of α-Li_2IrO_3 particle studied. Bright-field optical image of α-Li_2IrO_3 particle. Scale bar is 5 µm.

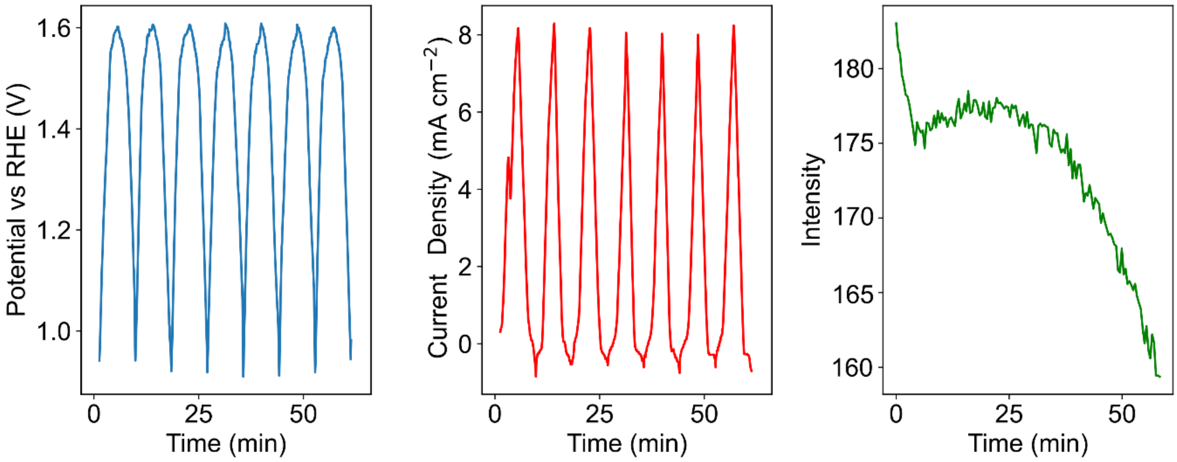

Supplementary Figure 21: Variation in the intensity of the 640 cm^{-1} mode of α-Li_2IrO_3 when cycling in LiOH. There is a drop in the 640 cm^{-1} mode intensity of α-Li_2IrO_3 associated with the delithiation in the first anodic cycle, followed by negligible (~10%) decrease in mode intensity in the subsequent scans. The voltage is not iR corrected.

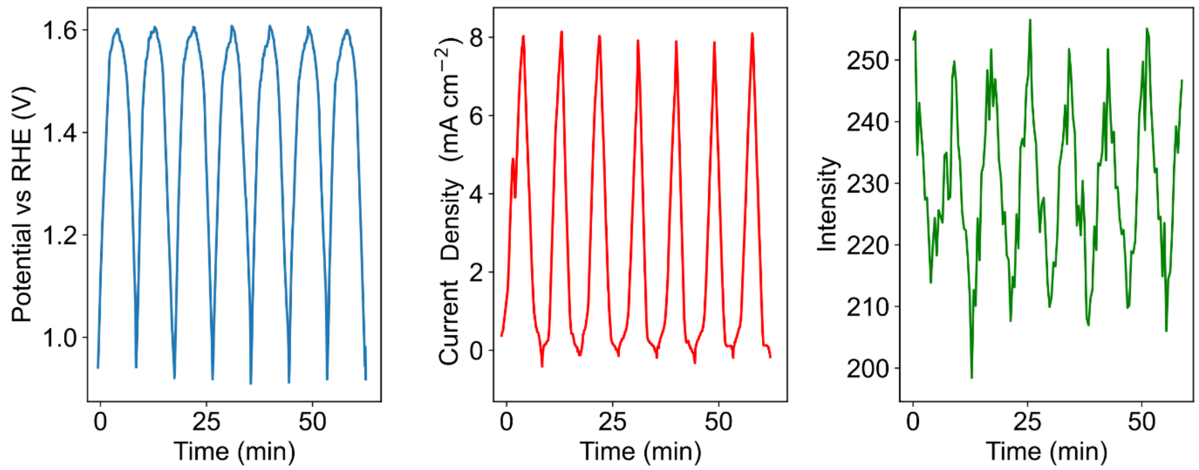

Supplementary Figure 22: Variation in intensity of the 550 cm^{-1} mode of α-Li_2IrO_3 when cycling in LiOH. A small 10% modulation in intensity is observed in the 550 cm^{-1} mode of α-Li_2IrO_3 when cycling in LiOH. The voltage is not iR corrected.

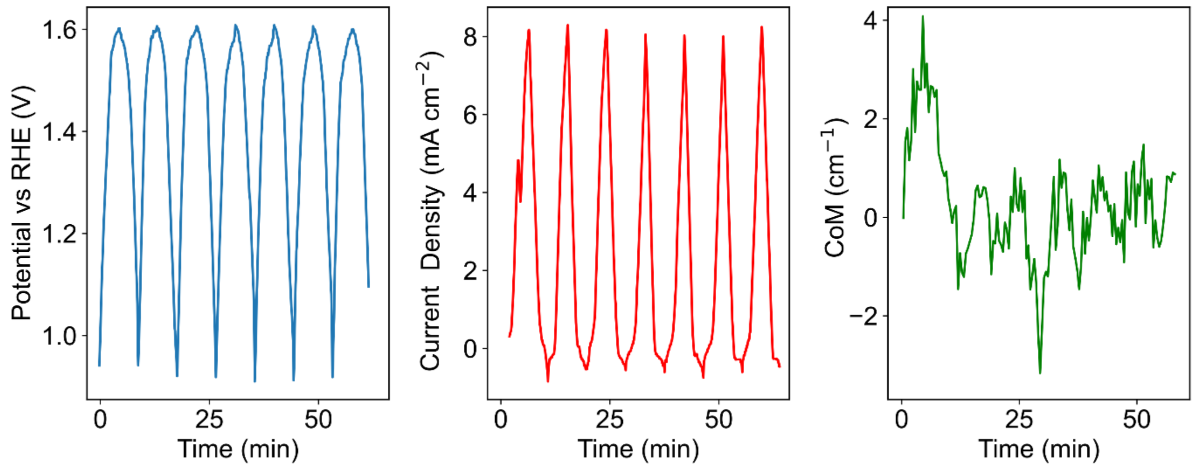

Supplementary Figure 23: 640 cm^{-1} mode of α-Li_2IrO_3 centre of mass changes during cycling. Variation in the CoM of 640 cm^{-1} mode of α-Li_2IrO_3 when cycling in LiOH. The voltage is not iR corrected.

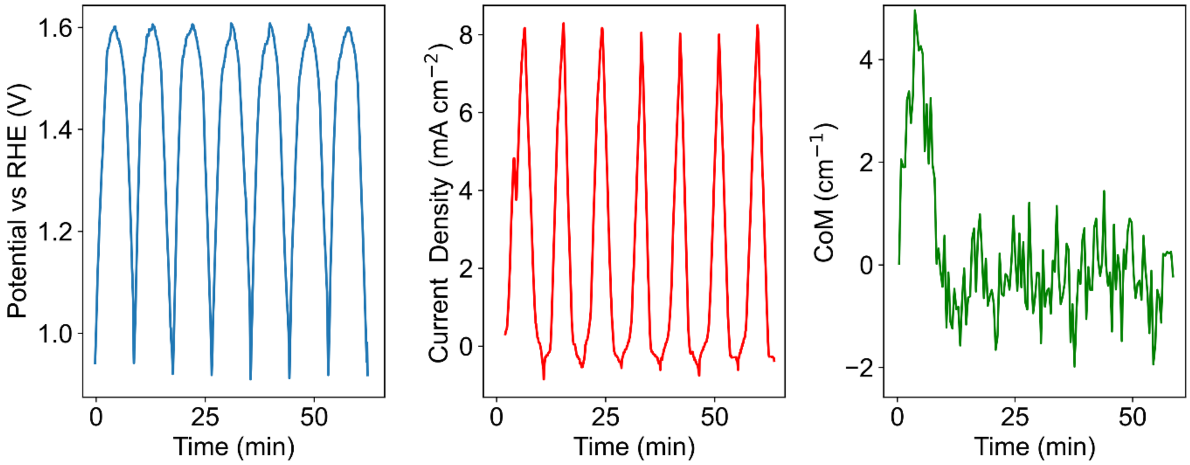

Supplementary Figure 24: 550 cm^{-1} mode of α-Li_2IrO_3 centre of mass changes during cycling. Variation in the CoM of 550 cm^{-1} mode of α-Li_2IrO_3 when cycling in LiOH. The voltage is not iR corrected.

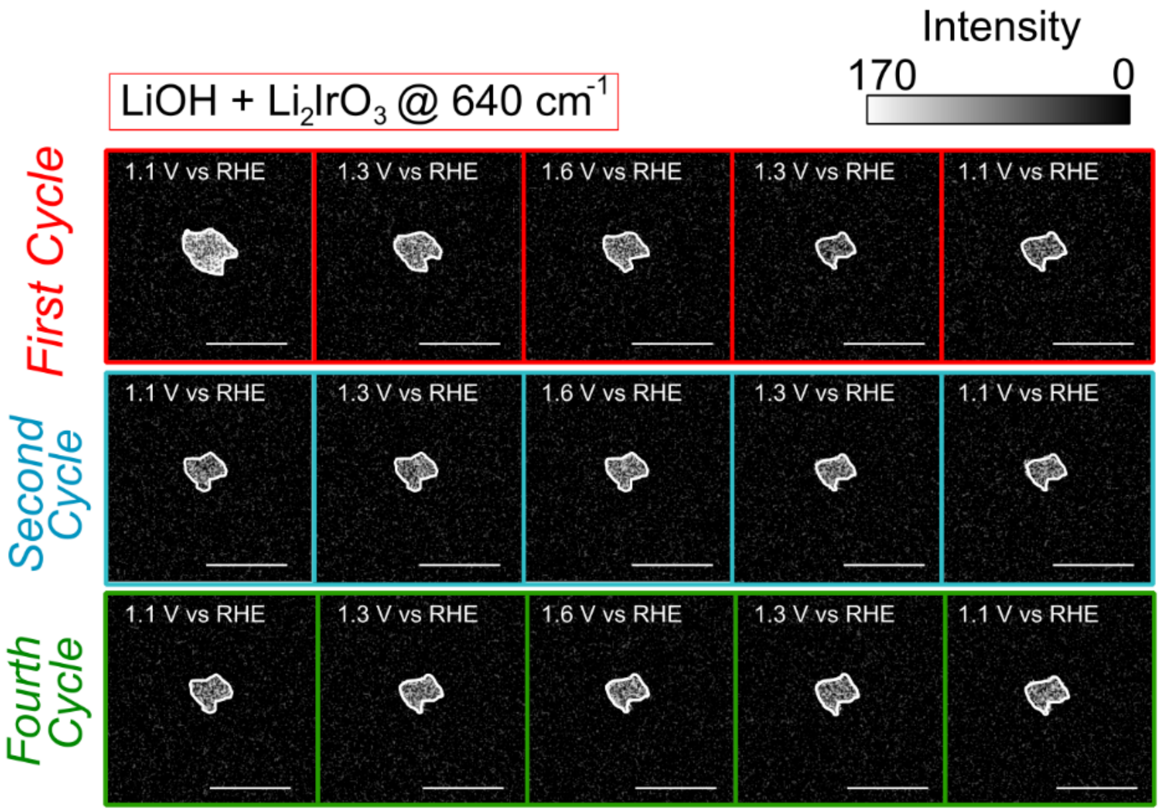

Supplementary Figure 25: Spatial distribution of intensity of 640 cm^{-1} mode, during cycling of α-Li_2IrO_3 agglomerate in LiOH. During the first anodic scan a shrinking core type pattern can be observed consistent with delithiation. The intensity of the 640 cm^{-1} does not

change in the cathodic scans and subsequent cycles in stark contrast to the core-shell behaviour observed in KOH. Scale bar is 5 μm. The voltage is not iR corrected.

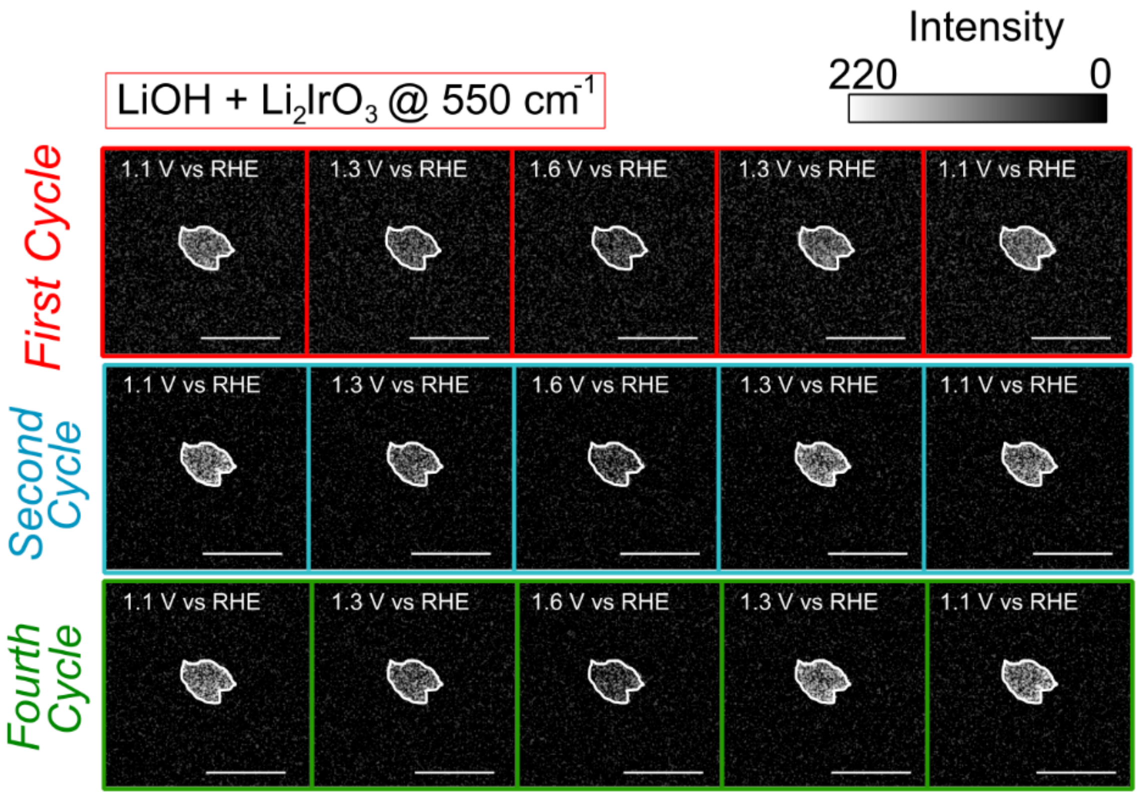

Supplementary Figure 26: Spatial distribution of intensity of the 550 cm^{-1} mode, during cycling of α-Li_2IrO_3 in LiOH. The mode intensity uniformly decreases (increases) across the particle during the anodic (cathodic) scan. Scale bar is 5 μm. The voltage is not iR corrected.

We note that the irreversible delithiation in the first cycle means it is not possible to repeat measurements on the same particle in different electrolytes making quantitative comparison of the change in Raman counts between experiments/electrolytes challenging. In **Supplementary Figure 27** we plot the change in Raman counts during the first cycle for 46 particles measured in LiOH and 46 measured in KOH. There is generally good agreement suggesting that on average the change in Raman intensity is similar between the two electrolytes. This same behaviour is revealed in a plot of the ratio of the intensity changes at 550 cm^{-1} and 640 cm^{-1} as shown in **Supplementary Figure 27b**. Furthermore, plotting the change in mode intensity at 550 cm^{-1} against that at 640 cm^{-1} in **Supplementary Figure 27c** reveals that magnitude changes in both are correlated for the first cycle. Together these results confirm that delithiation of α-Li_2IrO_3 is similar between both electrolytes.

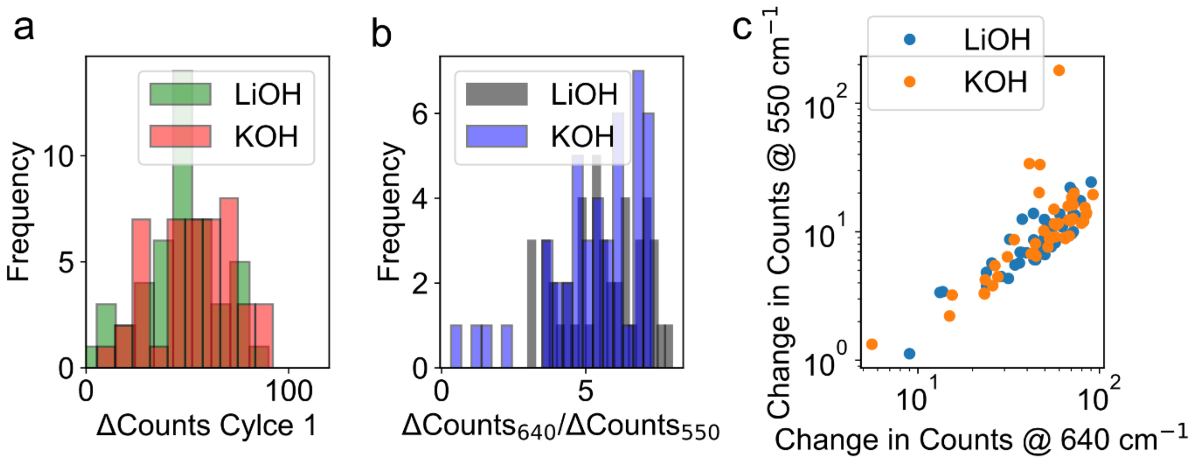

Supplementary Figure 27: Statistics of mode intensity changes when electrochemically cycling α-Li_2IrO_3. a. Change in counts of 640 cm^{-1} mode during first anodic scan of agglomerates in KOH (red) and LiOH (green). **b.** Ratio of change in counts of 640 cm^{-1} mode as compared to 550 cm^{-1} mode for agglomerates cycled in KOH and LiOH. **c.** Log-log scale plot of change in intensity of 640 cm^{-1} vs 550 cm^{-1} mode. There is a linear correlation between the change in intensity of both modes in LiOH and KOH.

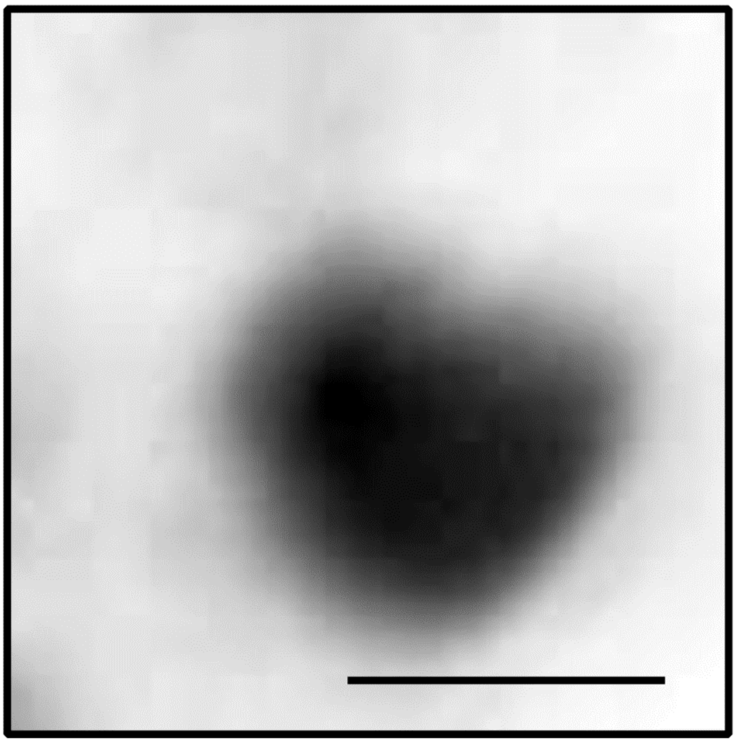

Supplementary Figure 28: IrO_2 particle studied. Bright-field reflection image of IrO_2 particle. Scale bar is 4 µm.

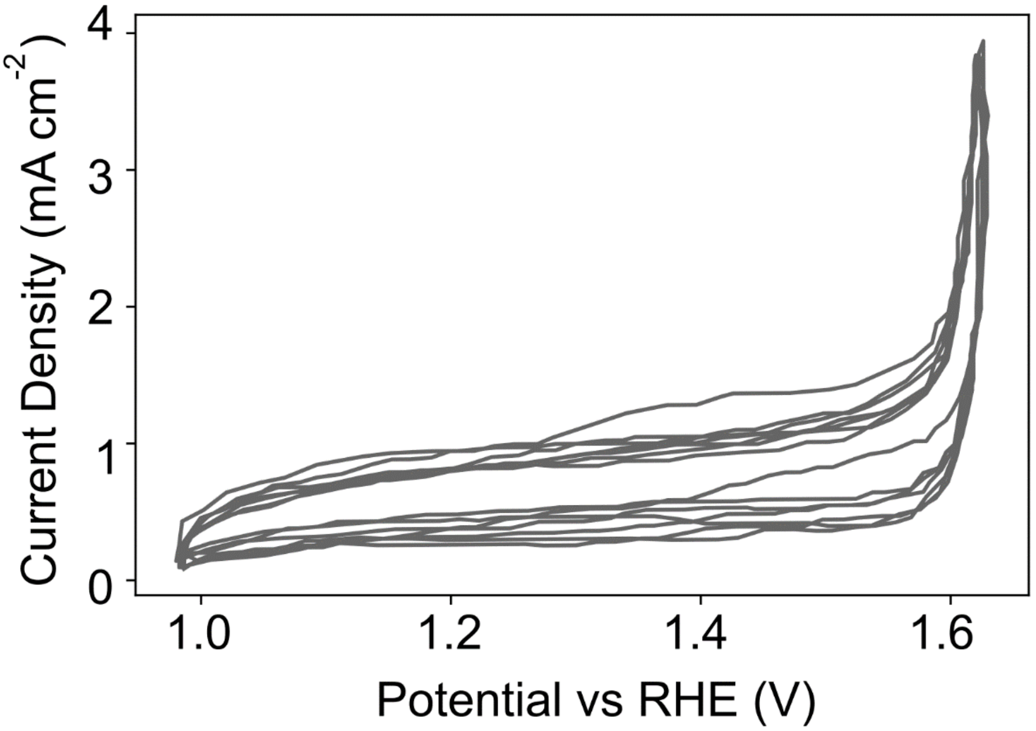

Supplementary Figure 29: Cyclic voltammogram of IrO_2 studied. Cyclic voltammogram of IrO_2 cycled in KOH over 8 cycles. The voltage is not iR corrected.

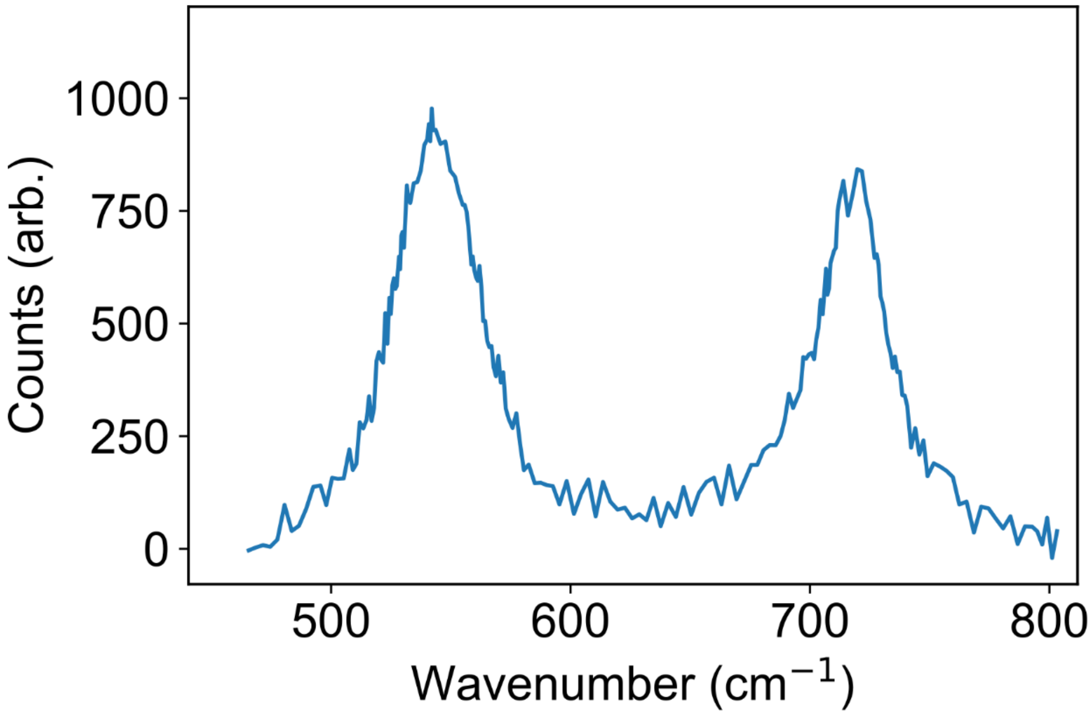

Supplementary Figure 30: Raman spectrum of IrO_2. The spectrum is in good agreement with previous studies[38–40].

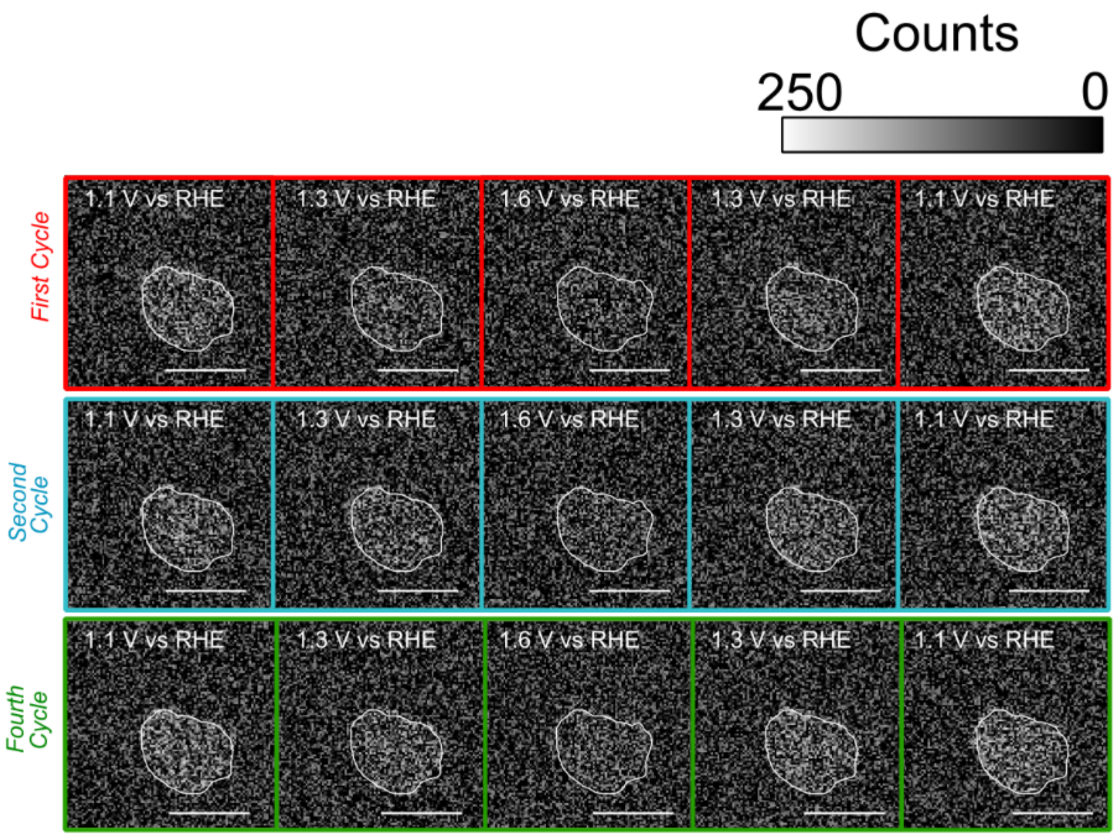

Supplementary Figure 31: Spatial intensity changes of 500 cm^{-1} mode for IrO_2. Spatial intensity distribution integrating over ~500 cm^{-1} mode for IrO_2 at given potentials and cycle number. The voltage is not iR corrected. Scale bar is 4 µm.

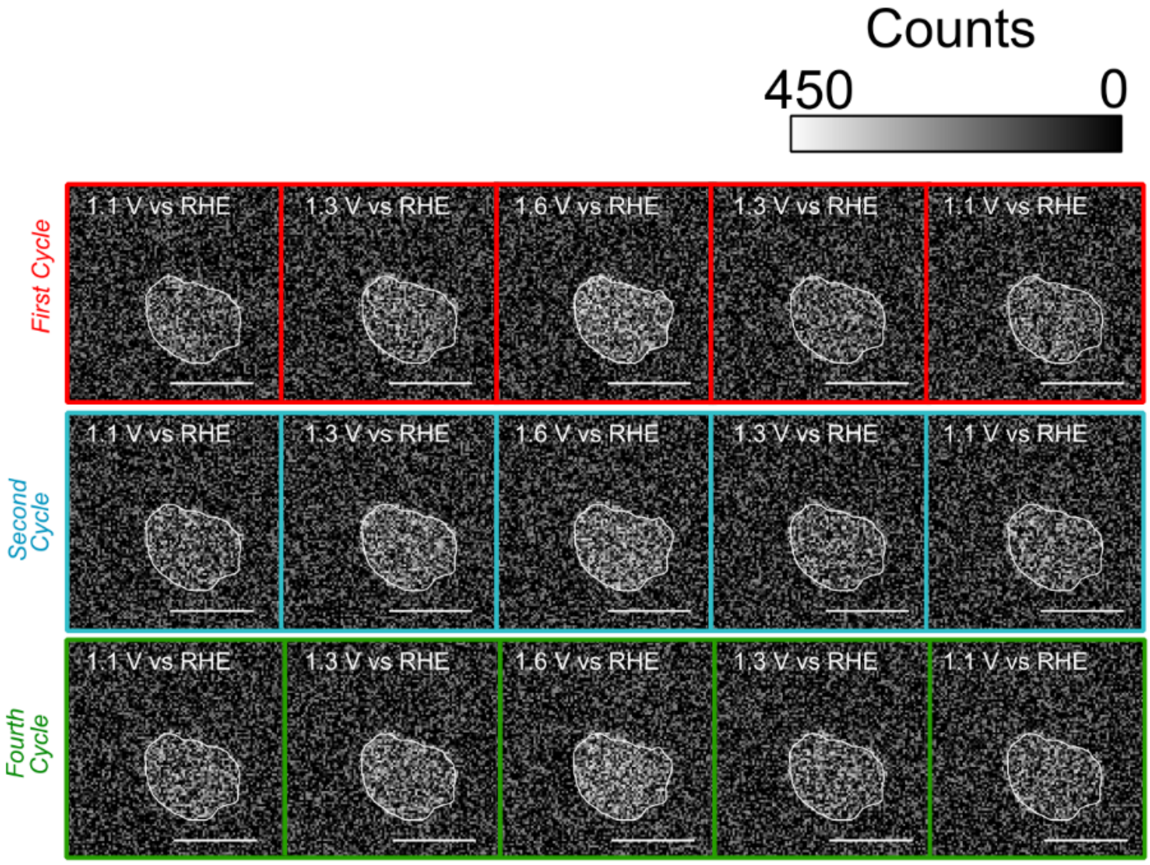

Supplementary Figure 32: Spatial intensity change of 700 cm^{-1} mode for IrO_2. Spatial intensity distribution integrating over 700 cm^{-1} mode for IrO_2 at given potentials and cycle number. The voltage is not iR corrected. Scale bar is 4 µm.

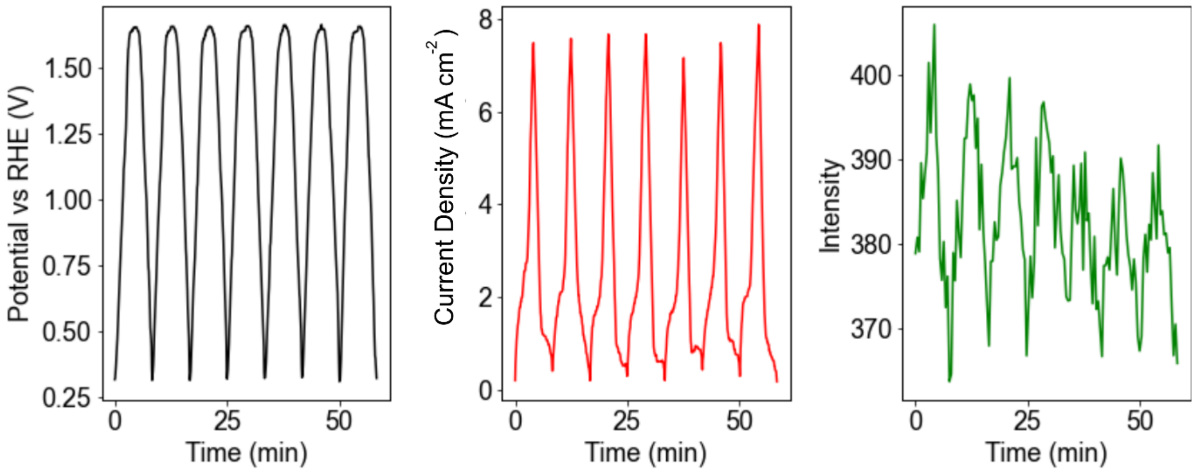

Supplementary Figure 33: Intensity change of 500 cm^{-1} mode for IrO_2. Intensity of 500 cm^{-1} mode for IrO_2 across multiple cycles. The voltage is not iR corrected.

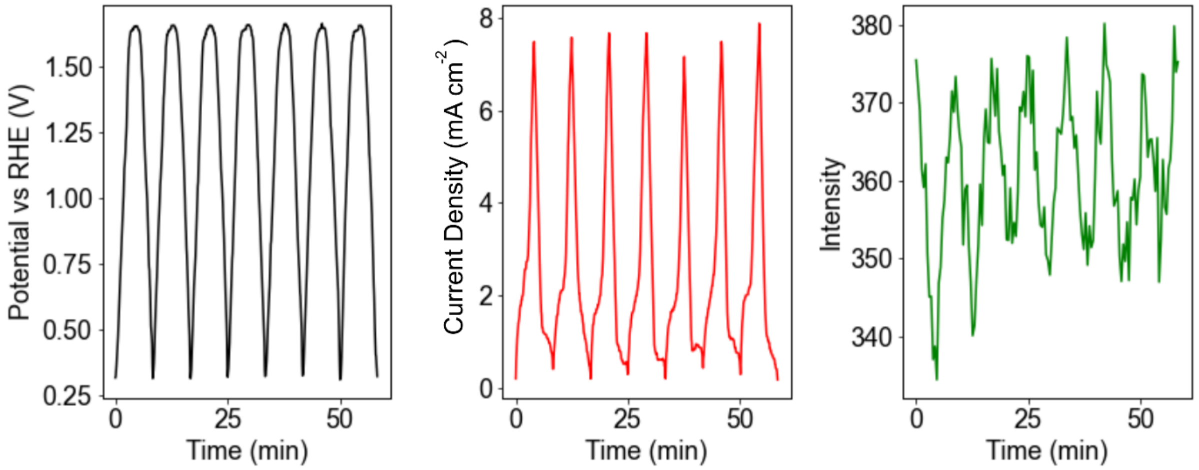

Supplementary Figure 34: Intensity change of 700 cm^{-1} mode for IrO_2. Intensity of 700 cm^{-1} mode for IrO_2 across multiple cycles. The voltage is not iR corrected.

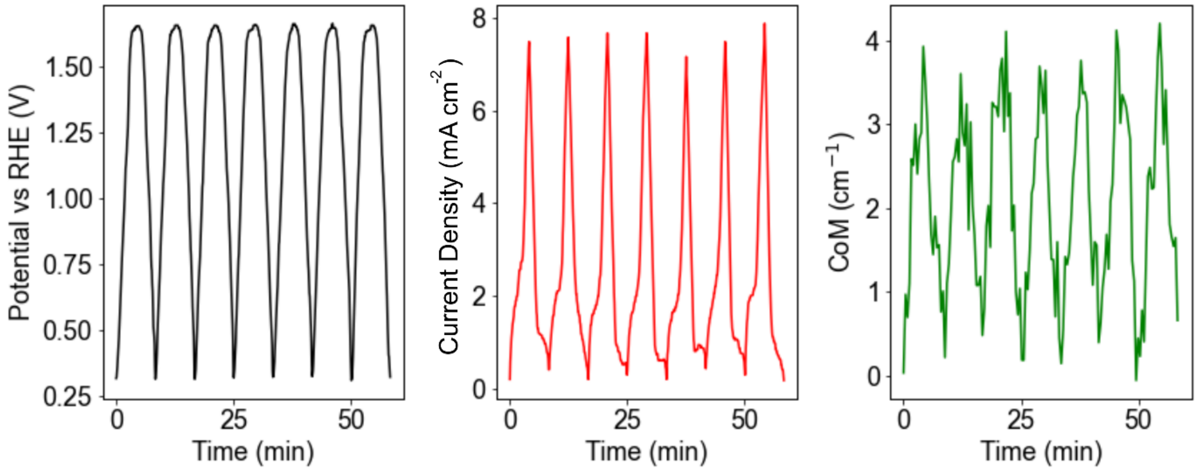

Supplementary Figure 35: Centre of mass (CoM) changes of 500 cm^{-1} mode of IrO2 during electrochemical cycling. CoM of 500 cm^{-1} mode for IrO_2 across multiple cycles. The voltage is not iR corrected.

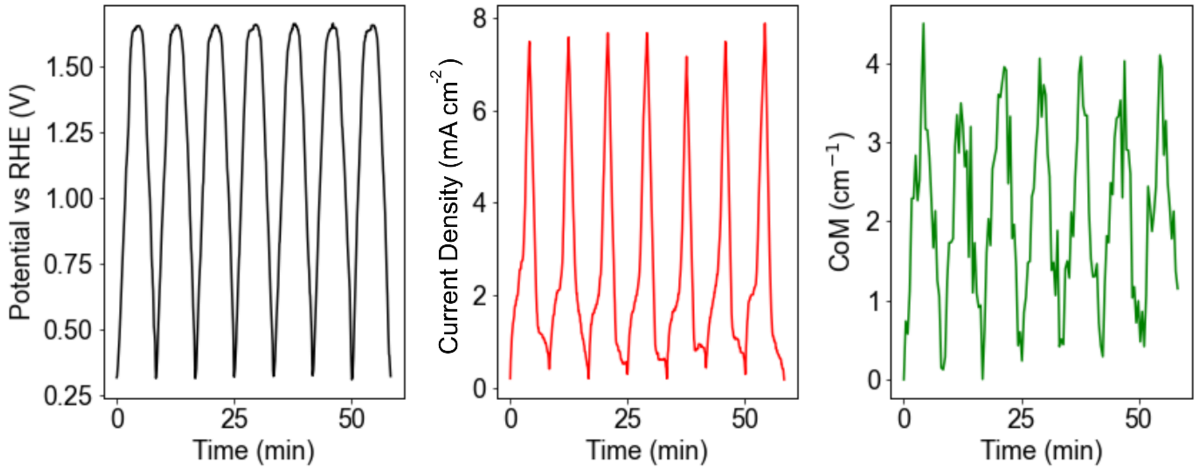

Supplementary Figure 36: Centre of mass (CoM) changes of 700 cm^{-1} mode of IrO2 during electrochemical cycling. CoM of 700 cm^{-1} mode for IrO_2 across multiple cycles. The voltage is not iR corrected.

Supplementary Note 12: Operando Raman imaging of $LiCoO_2$ in KOH

We also perform Raman imaging of LCO agglomerates (**Supplementary Figure 37**). As detailed in refs[41–43], similar behaviour might be expected for LCO as compared to α-Li_2IrO_3 when cycled in KOH, albeit with higher overpotentials. In **Supplementary Figure 38** we show that we obtain a similar 'shrinking core' pattern of delithiation and potassium intercalation during the first anodic and cathodic scan, respectively. All imaging is performed at the A_{1g} band (590 cm^{-1}) of LCO, which reports on out-of-plane lattice changes[44,45], to make comparison with the behaviour seen at the equivalent 640 cm^{-1} band of α-Li_2IrO_3. Although for LCO the changes in intensity during the delithiation and potassium intercalation likely arise from a changes in the attenuation length as opposed to stretching/scissoring. During the subsequent ($n \geq 2$) anodic scans, a 'core-ring' pattern (albeit one that is less clear than for α-Li_2IrO_3) emerges. Such similar observations in a different intercalation electrocatalyst provides further support to the robustness of our Raman imaging and indicates the generality of our finding that for such crystalline catalysts charge compensation can take place beyond the surface metal sites.

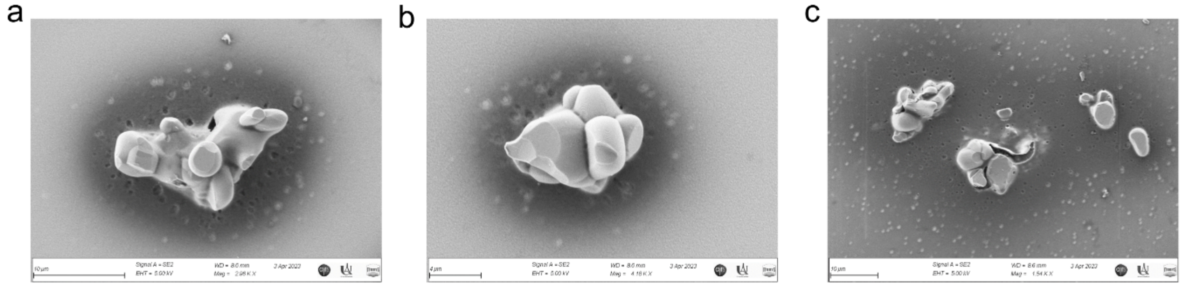

Supplementary Figure 37: SEM images of LCO agglomerates. a-c. SEM images of LCO agglomerates studied deposited with Nafion. The scale bars are indicated in the figure.

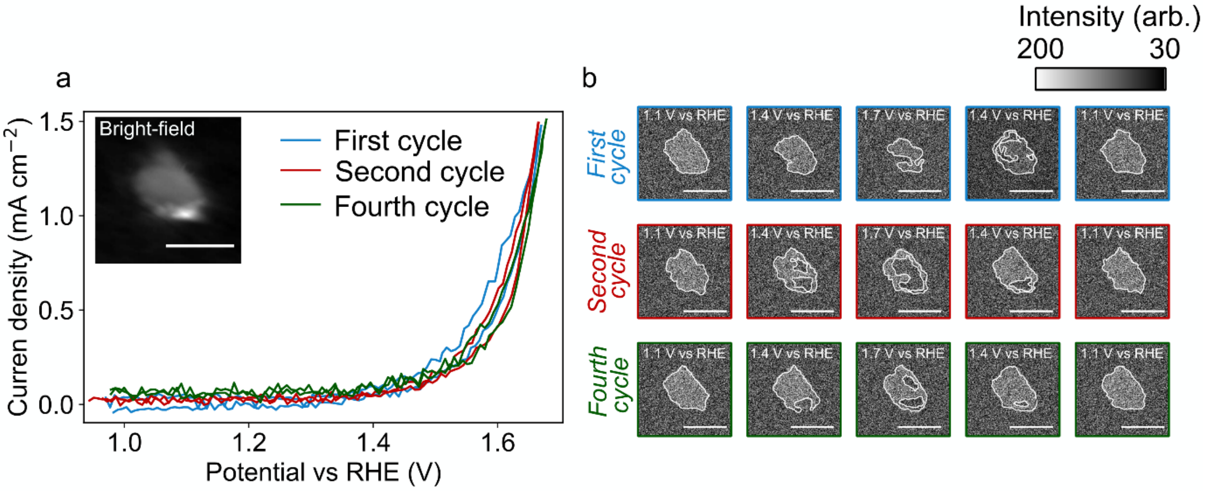

Supplementary Figure 38: Concurrent Raman imaging and electrochemical cycling of $LiCoO_2$ agglomerates. a. CV associated with first three cycles of $LiCoO_2$ agglomerates (mixed with Nafion) in KOH. The voltage is not iR corrected. Unlike for α-Li_2IrO_3 no clear delithiation peak can be observed in the first anodic scan, although this process still occurs in this material. Inset shows bright-field optical image. Scale bar is 3 µm. **b.** Compressive Raman imaging of catalyst during cycling. Imaging is at 590 cm^{-1} (out-of-plane A_{1g} band). A similar core-shell pattern to that observed in α-Li_2IrO_3 in the first anodic scan is observed, followed by a ring like build-up of Raman intensity in the subsequent anodic scans (clearest in the second cycle). Scale bar is 3 µm.

Supplementary Note 13: Operando Raman imaging with KOH electrolyte of α-Li_2IrO_3 at 550 cm^{-1} and over multiple particles

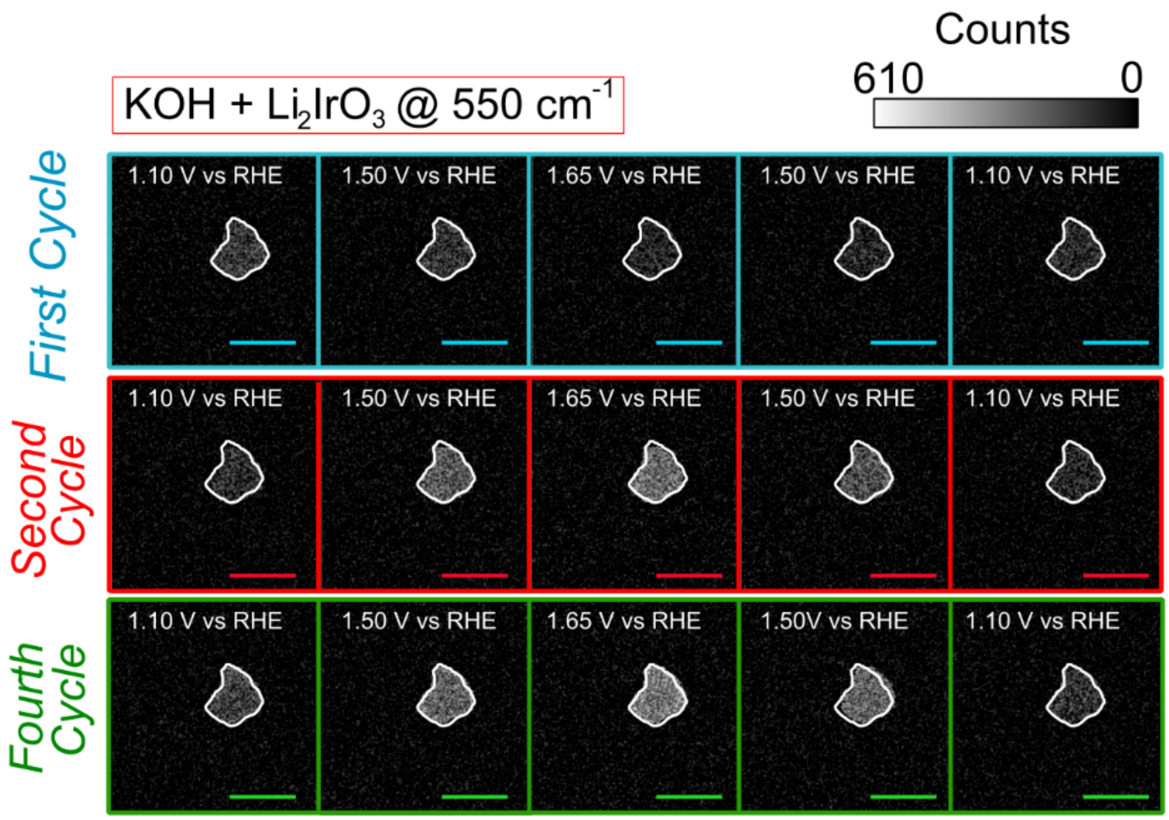

Supplementary Figure 39: Spatial distribution of intensity of 550 cm^{-1} mode during cycling of the α-Li_2IrO_3 agglomerate particle shown in main text in KOH. In the first cycle a dimming of the mode intensity, uniformly across the particle, is seen on the anodic scan followed by a brightening on the cathodic scan. For subsequent scans (≥ 2), the anodic scan results in brightening of the particle and then dimming on the cathodic scan. Further, cycles are shown in **Supplementary Videos 1-2**; in the videos we note the mode intensity counts are arbitrary. Scale bar is 5 μm. The voltage is not iR corrected.

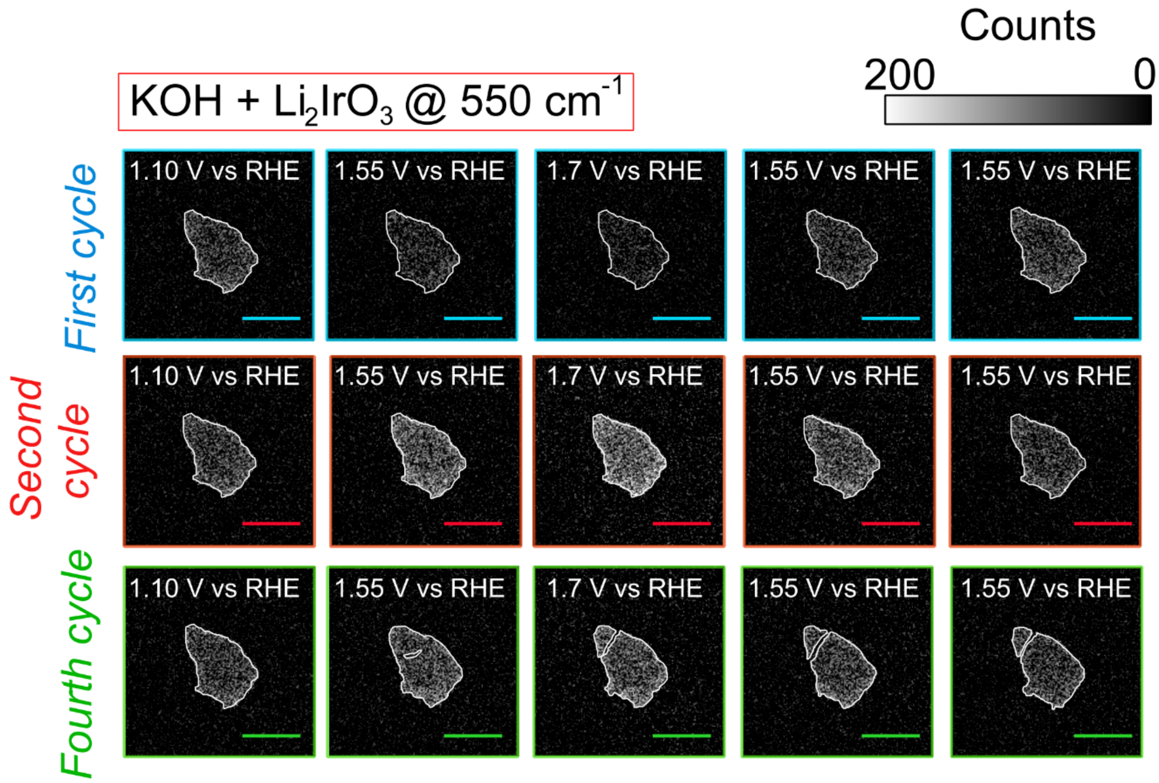

Supplementary Figure 40: Spatial distribution of intensity of 550 cm^{-1} mode during cycling of a second α-Li_2IrO_3 agglomerate particle in KOH. In the first cycle a dimming of the mode intensity, uniformly across the particle, is seen on the anodic scan followed by a brightening on the cathodic scan. For subsequent scans (≥ 2), the anodic scan results in brightening of the particle and then dimming on the cathodic scan. Further, cycles are shown in **Supplementary Videos 3-4**; in the videos we note the mode intensity counts are arbitrary. Scale bar is 5 μm. The voltage is not iR corrected.

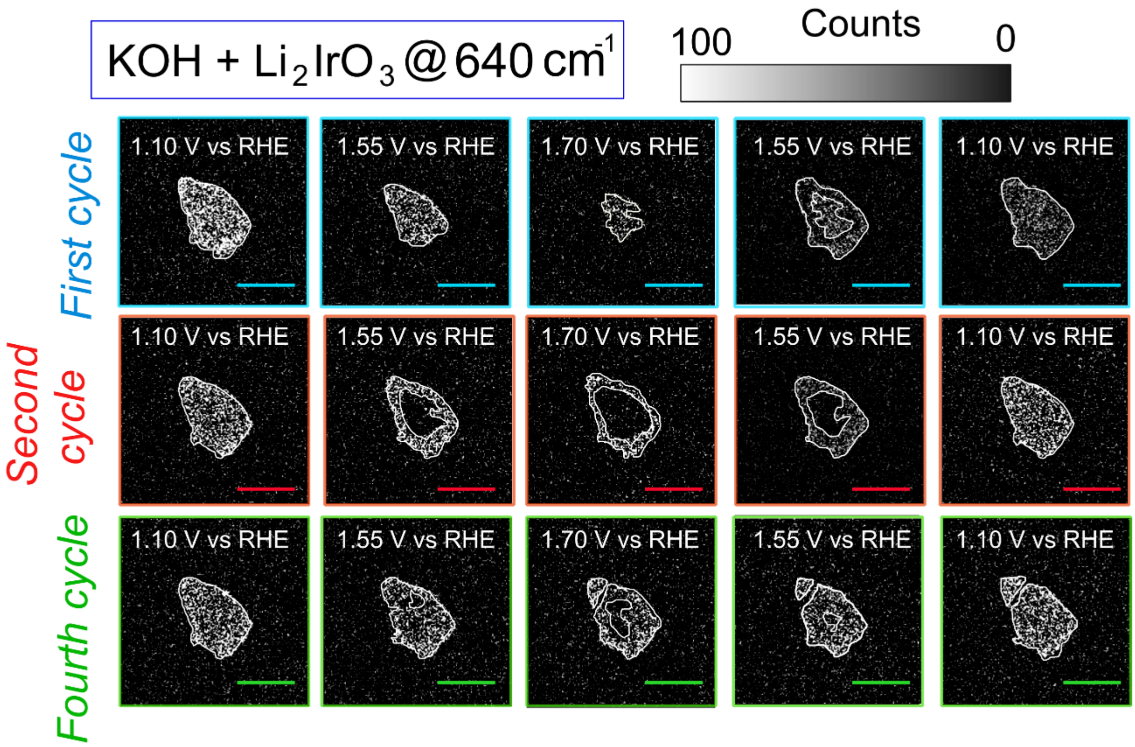

Supplementary Figure 41: Spatial distribution of intensity of 640 cm^{-1} mode during cycling of a different α-Li_2IrO_3 agglomerate particle to that considered in main text in KOH. Although a similar pattern of behaviour to that observed in the main text is observed, for this agglomerate full delithiation does not occur during the first cycle and in the fourth cycle there is cracking/fatiguing of the particle which partially obscures the reaction fronts. The scan rate is 4.0 mV s^{-1}. Scale bar is 5 μm. The voltage is not iR corrected.

Supplementary Note 14: *Operando* gas bubble imaging

It is interesting to examine the relationship between where intercalation occurs on the agglomerates and where O_2 is evolved. To do so we perform optical imaging experiments of the microbubbles evolved during OER (see **Methods** in main text).

Microbubbles, which appear as a region of dark circular intensity, with a bright central spot, in bright-field images are observed to form after ~1.35 V vs RHE, reaching a maximum lateral extent at the maximum of the potential (1.7 V vs RHE; see **Supplementary Figure 42**). On the cathodic scan bubbles begin to shrink but do not necessarily collapse fully, nor do they begin to shrink immediately after reversing the potential. To estimate the size of bubbles we follow the methods of Lemineur *et al.*[46], by first generating a differential image where the agglomerates and background substrate are subtracted from the image. A binary image is then created and the Hough circle transform method[47] is then used to estimate the radius of the bubble (**Supplementary Figure 43**). Bubbles are assumed to be hemi-spherical in shape which is a reasonable assumption given their size. The volume of the bubble V_B is then estimated as $\frac{\pi}{24}\frac{D^3}{\sin(\theta)^3}(2+\cos(\theta))(1-\cos(\theta))^2$, where θ, the contact angle with the surface, is taken to be 90° and D is the bubble diameter extracted from the 2D optical images[48]. Taking measurements across ~30 agglomerates we obtain bubble volumes of 20 to 60 μm^3 (±5 μm^3 fitting and measurement error on each volume value) after 30s (i.e., after reaching 1.7 V vs RHE). The amount of O_2 evolved n_{O_2} after 30s can then be estimated from $\frac{V_B P_{gas}}{k_B T}$ where P_{gas} is taken to be $P_0 + \frac{4\gamma}{D}$ (P_0 is 1 atm and γ is the surface tension of the solution 73 mN/m)[49]. Using these equations and the estimated bubble volumes we obtain a histogram of the amount of gas evolved which ranges between 2.5 and 8 × 10^9 molecules per agglomerate. The rate of gas formation is estimated to be about ~0.15 ×10^9 molecules s^{-1} per agglomerate, with the rate being relatively linear with potential between 1.37 and 1.52 V vs RHE. Beyond 1.52 V vs RHE the measured gas evolution rate begins to slow. This could be due to geometric factors associated with large bubble growth, i.e., a singular large bubble can no longer support itself.

Using the centre-of-mass of the bubbles in the frame when they first appear we can estimate where gas is evolved from/nucleation sites (**Supplementary Figure 44**). We note on some agglomerates there are multiple bubbles generated initially on the agglomerate which then merge. We also caveat our observations with the fact that gas evolution in these systems is very

fast and despite our time resolution of up to ~0.1s, smaller nano-bubbles could be nucleating from sites below our resolution (400 nm spatial resolution). Nonetheless, we observe that bubbles nucleate from both the top surface of agglomerates and the agglomerate edges. From our measurements over 30 agglomerates it appears that statistically bubbles evolve more often from the agglomerate edges (~75%; **Supplementary Figure 45**).

Nucleation sites for the bubbles do not necessarily correlate with the most active site, especially in a system as complex as ours. However, our observations are in keeping with the notion of agglomerates where reactivity may occur both on the edges and top-surface.

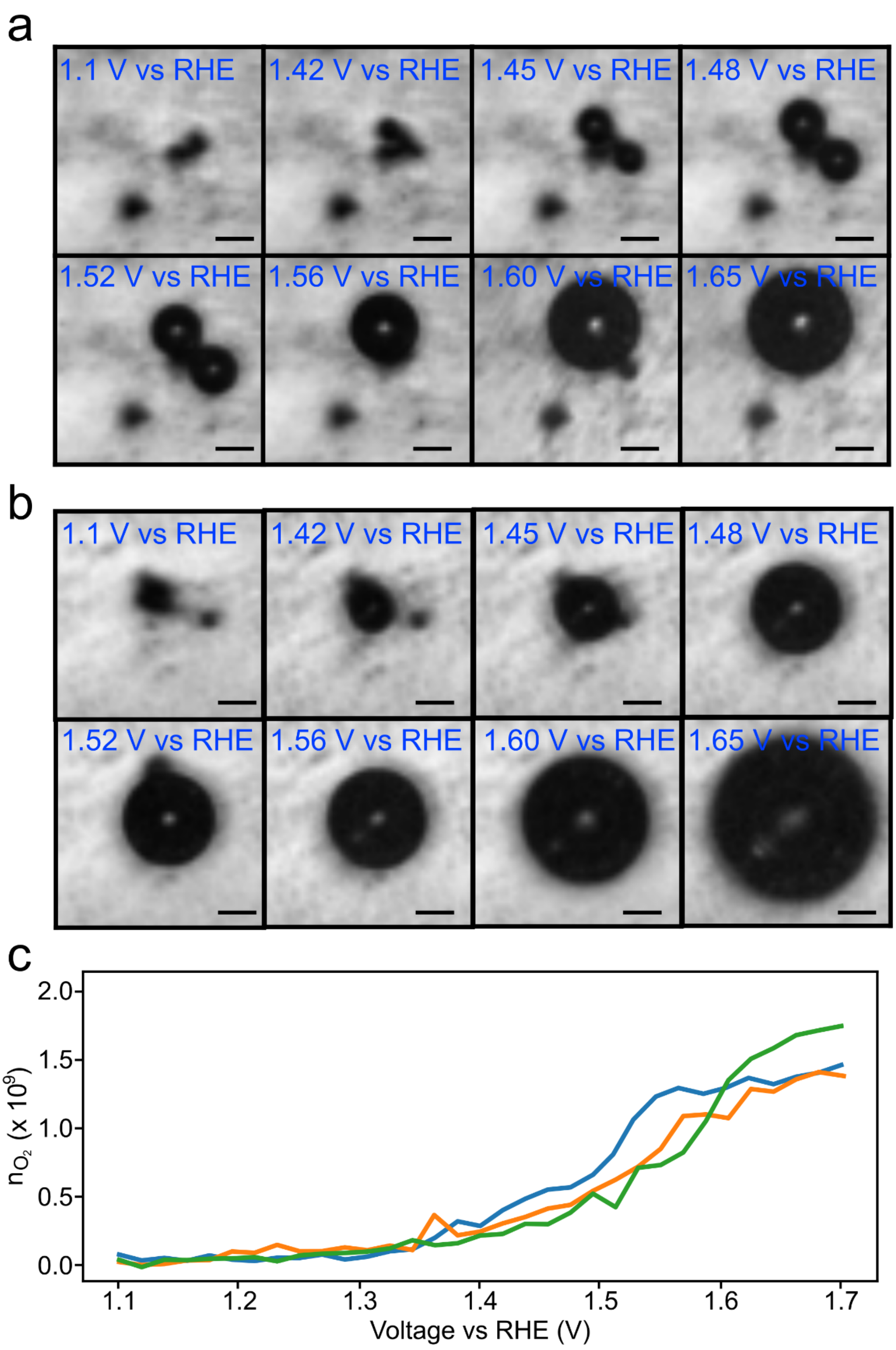

Supplementary Figure 42: Imaging gas bubble evolution during cycling of α-Li_2IrO_3. a-b. Bright-field images of agglomerates as O_2 gas is evolved (imaging from the top of agglomerates through electrolyte). Dark circles show bubble(s) forming on agglomerates as

potential is increased (blue). **c.** Moles of O_2 gas evolved as a function of potential for 3 different agglomerates. The voltage is not iR corrected. Scale bar is 2 μm in all images.

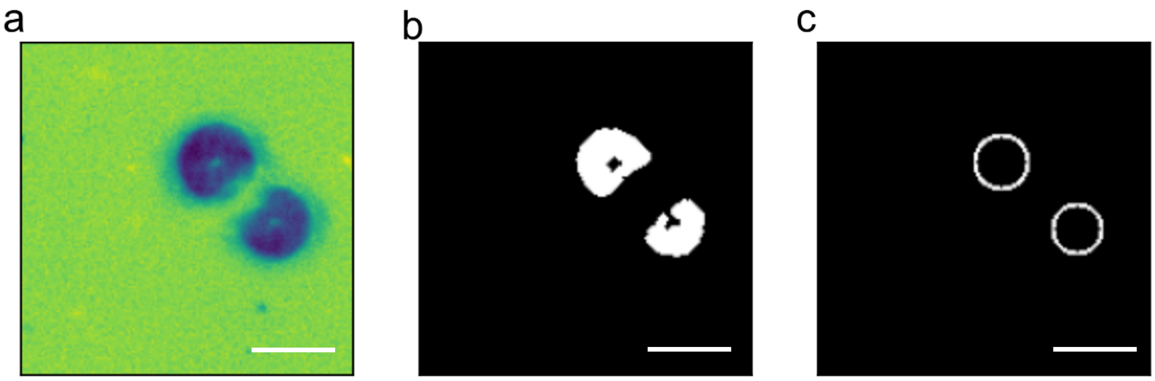

Supplementary Figure 43: Extraction of bubble size during gas bubble imaging from α-Li_2IrO_3 as it is cycled. a. Optical image of bubbles following subtraction of background and agglomerates. **b.** Binarised image using the Otsu method. **c.** Identification of circles on image using Hough circle method, with circle radii highlighted. Scale bar is 2.5 μm in all images.

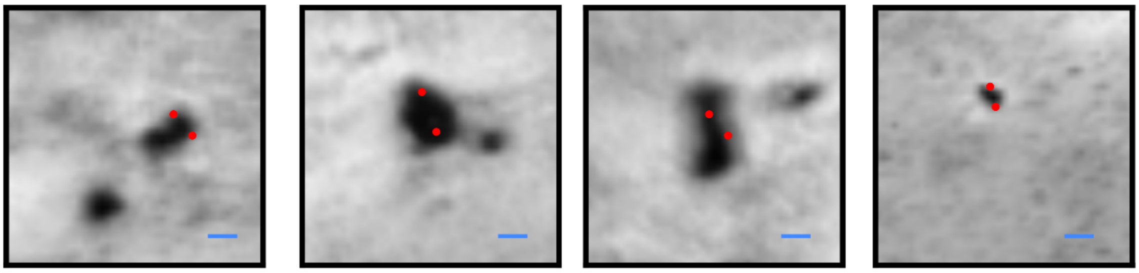

Supplementary Figure 44: Determining the bubble nucleation location during gas bubble imaging from α-Li_2IrO_3 as it is cycled. a. Brightfield images of agglomerates with observable nucleation sites of bubbles marked as red circles. Scale bar is 2 μm in all images.

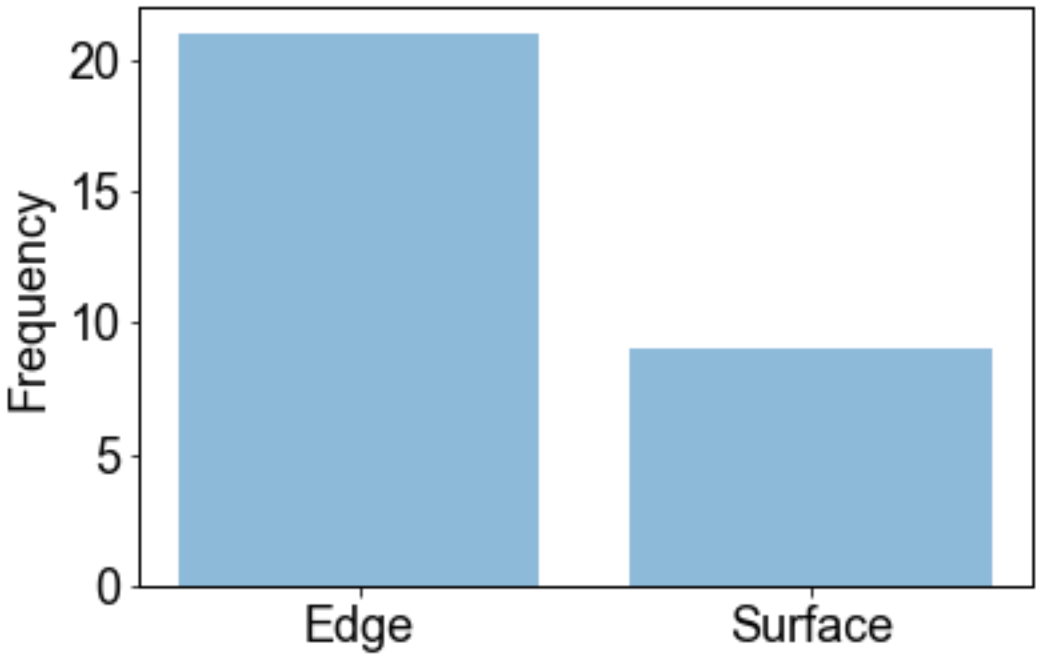

Supplementary Figure 45: Histogram of bubble evolution location. Histogram counting the number of agglomerates where bubbles are generated towards the edge of agglomerates as compared to the top surface. Measurements taken over 30 agglomerates.

The above data underscores the fact that as we go to higher current densities/potentials the number of O_2 molecules generated per s will increase. At some point this rate of gas evolution will go beyond the cation intercalation velocity which remains constant and cannot meet the O_2 generation rate to maintain the electrochemical-chemical pathway for charge compensation.

Supplementary Note 15: Estimation of cation (de)intercalation velocities

The approach we use to determine front velocities/diffusion lengths follows that previously outlined for ion diffusion in conjugated polymers[50] and battery materials[51]. Although in our case the overall diffusion in agglomerates is 3D, because we are viewing a 2D projection we believe this approach is appropriate to obtain an estimate of intercalation velocities at the single agglomerate level.

Partially averaging along the spatial dimension perpendicular to the in-plane direction of phase front propagation allows for improved signal-to-noise. All velocities/diffusion lengths were obtained from the anodic scan using the following protocols:

(i) For the first delithiation cycle the time window the data is examined in is that taken to scan from 1.35 V_{RHE} (when delithiation begins) to 1.65 V_{RHE} at 4 mV/s. The centre of the agglomerates is defined by their centre-of-mass. Four regions (orthogonal to one another) are then chosen to measure the front propagation over. The spatial direction the front is measured over is approximately perpendicular to the particle edge and towards the centre-of-mass. In the case of full delithiation we are typically examining the front propagating from the agglomerate boundary to the centre-of-mass, but we strictly stick to the above time window for all four regions of the agglomerate i.e., in some cases the front does not reach the agglomerate centre-of-mass. The velocity values obtained are averaged over the four regions to give the overall front velocity for a given agglomerate.

(ii) For the second, third and fourth anodic scans the same method as above is used. Except the front is taken as it propagates from the particle centre-of-mass towards a given edge. To ensure consistency we evaluate the data only from ~1.35 V_{RHE} to 1.50 V_{RHE}, i.e., the potential region dominated by potassium deintercalation.

(iii) For experiments where we release the potential the same protocol as detailed in (i) is used except the time period used is that from when the potential is released to when the mean intensity taken across a 500 nm^2 region at the particle centre-of-mass is within one standard deviation of that at the boundary (taken as a strip of pixels 500 nm wide tracing the boundary) i.e., Raman intensity across the agglomerate has homogenised.

(iv) For examining the width of the boundary we examine the distance between the points where the normalised Raman signal along a cross-cut lies between 0.2 and 0.8.

(v) In order to fit the data and extract velocities (see below), data in some cases is pre-smoothed with a Gaussian filter. The disadvantage of this filter is that it can artificially broaden features in data. To ensure this is not occurring we check that the gradient of the boundary between the particle and background in the Raman images does not increase by more than 5% after filtering (which is within the uncertainty of the experiment itself).

We believe the above criteria set out a both scientifically justified and robust approach to extract velocities. Whilst some degree of judgement is also involved in setting out these criteria, we re-emphasise the trends and orders of magnitude which are most important to our conclusion do not vary with choice of exact extraction region.

To account for measurement uncertainties in both the pixel intensity (based on the signal-to-noise ratio of experiments) and position uncertainty (based on our spatial resolution) we use the approach of a 'weighted' non-linear uncertainty when fitting any data. Each data point in a fit is weighted by $\frac{1}{\Delta I^2 \times \Delta x^2}$ where ΔI is the uncertainty in the pixel intensity and Δx is the error on the spatial position. In this way a normally distributed error based on the measurement error in position can be incorporated into our fit to give an overall variance. This gives an error on the iso-intensity position we extract which we then propagate into our fit for the velocity. This measurement and fit uncertainty is then incorporated into the error bars which also account for the variation across repeat experiments. **Supplementary Table 4** summarises the typical errors obtained in experiments.

Experimental quantity	Uncertainty
Raman pixel intensity	~0.4% per pixel (from standard deviation of noise)
Raman pixel position	300 nm (3 pixels in our configuration)
Bright-field bubble imaging	500 nm (6 pixels in our imaging configuration)

Supplementary Table 4: Table of measurement uncertainties for techniques explored in this work. Table summarising uncertainty in various measurement techniques used in this text.

Finally, to extract the velocities themselves line-cuts are taken along four approximately orthogonal edges of the agglomerate. A maximum of 10 pixels are averaged together in the spatial dimension perpendicular to the in-plane direction of phase front propagation to improve signal-to-noise. Overall this procedure results in a step-like phase boundary. The centre-of-mass of agglomerates is then defined. A binary image of the particle is first calculated using Canny edge detection[52]. The centre-of-mass is then determined as $\gamma_{COM} = \frac{\sum_i \gamma_i \times m_i}{\sum_i m_i}$ where γ is the x or y coordinate. The process is illustrated in **Supplementary Figure 46**. The contrast in Raman signal between the agglomerates and Nafion/background can be used to define the agglomerate edges. The line-cuts are fit with a sigmoid function of the form $I_{Raman} = \frac{L}{(1+e^{-k(x-x_0)})} + b$ where x_0 is the centre of the sigmoid, L is the top to bottom height, $1/k$ represents the interface gradient and b is an intensity offset. Before fitting Gaussian smoothing is applied to the line-cuts to improve the fit whilst ensuring it does not change the gradient of the boundary in the raw data visually. Once fit a plot of x_0 is made a function of time. Within the regions defined above a straight line fit of x_0 *versus* time is made. From the gradient of this plot (for all four regions of the agglomerate examined) a velocity for the given phase-front of interest can be estimated. All velocities are obtained from the anodic scan. Several examples of how we determine the velocities are shown in **Supplementary Figures 47-50**.

We emphasise at the single crystal level transport of ions into α-Li_2IrO_3 would be expected to be two-dimensional based on its layered structure. However, we are visualising agglomerates. Based on the SEM images these do tend to have a slight preferential 2D stacking within agglomerates however, this is (i) not necessarily true for all agglomerates and (ii) cannot be determined from our optical imaging. Furthermore, what we image is a 2D projection of what at the agglomerate level is an overall 3D process. In light of this complexity fitting any pre-supposed equation to the motion we actually observe is likely artificial. Most importantly, examining a plot of the phase-front boundary position *versus* time we observe a linear response. Therefore, using a velocity term is appropriate for examining the potassium re-intercalation both under an applied potential and after the potential has been released. We note that this velocity approach is also used in the case of characterising transport in 3D single crystal layered systems where pure 2D motion is being imaged (see: ref[51]). We only extract velocities from the anodic scan where there is ion deintercalation. In this case our 2D imaging cannot fully capture the flux towards the core of the agglomerates and hence our velocity values although are likely within the correct order of magnitude will not be fully exact.

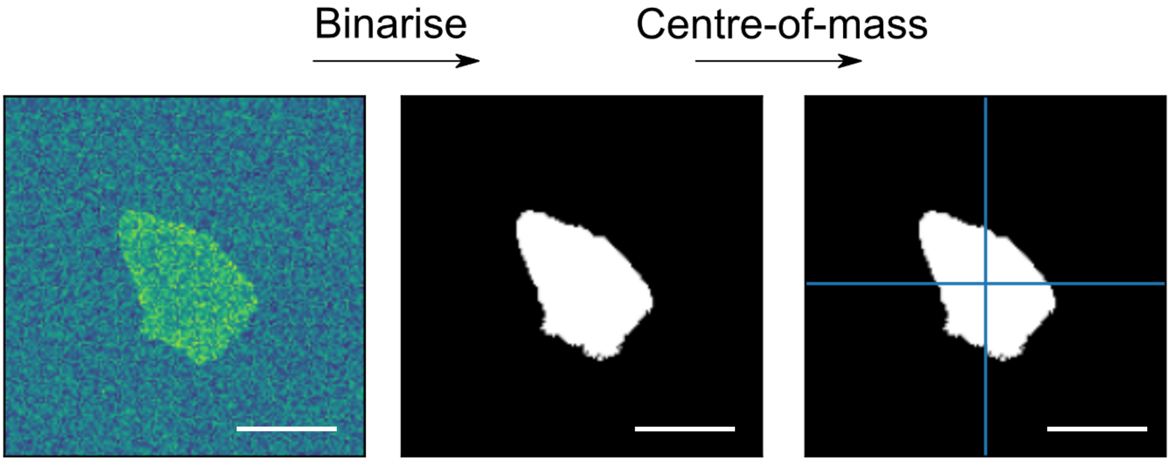

Supplementary Figure 46: Extracting particle centre-of-mass from Raman imaging of α-Li_2IrO_3. Raman image of α-Li_2IrO_3 agglomerate (left) at 640 cm^{-1} which is then binarised as described above (centre) and it's centre-of-mass located. Scale bar is 5 μm.

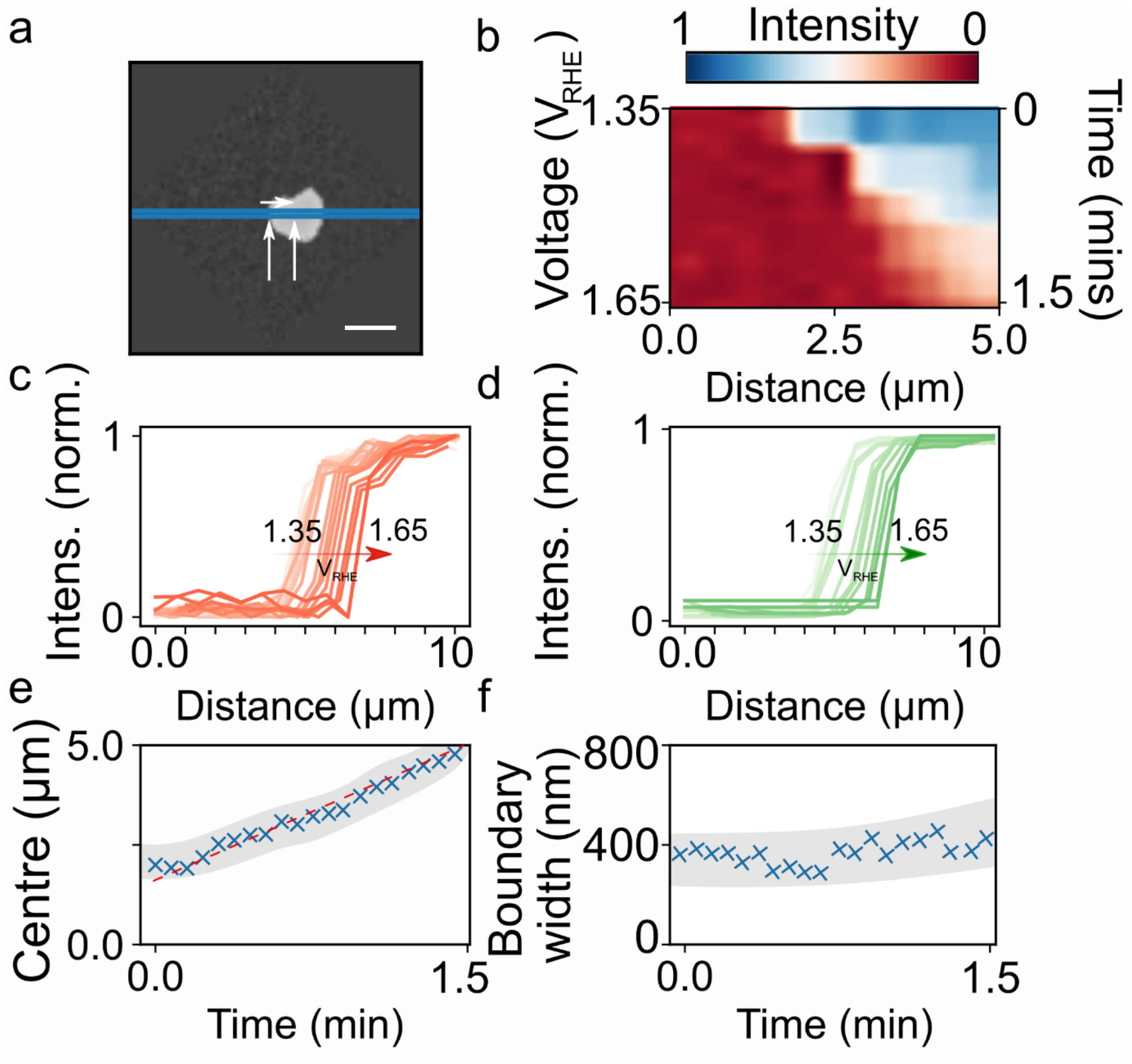

Supplementary Figure 47: Determining the mode front velocity during the first anodic scan of α-Li_2IrO_3 in KOH. Re-orientated Raman image of α-Li_2IrO_3 integrating across 640 cm^{-1} mode with Gaussian smoothing applied. Horizontal white arrow shows direction of moving front, which is measured to move between the agglomerate boundary and centre-of-mass (central white arrow). Blue lines show the region of pixels integrated across to improve the signal-to-noise ratio of the line cuts. Scale bar is 5 μm. **b.** Normalised stacked line-cuts of Raman image (integrating across 640 cm^{-1}) as a function of time/potential for the first anodic scan of the material in KOH. **c.** Line-cuts across from map in **b** shown as line-plot. Not all line-cuts are shown. Arrow/shading indicates increasing potential/time. **d.** Fit of line-cuts shown in c using sigmoid function (see above for equation). Not all line-cuts are shown. Arrow/shading indicates increasing potential/time**. e.** Position of fitted sigmoid as a function of time. Dotted red line shows straight-line fit from whose gradient the non-voltage normalised 'phase-front' velocity can be estimated. Grey shading indicates cumulative measurement (on Raman pixel intensities and front position) and fitting uncertainty. Not all points could be fitted due to the noise and are subsequently excluded. **f.** Phase-front boundary width, as estimated from the region in which the normalised sigmoid line cut lies between 0.2 and 0.8, as a function of time. Grey shading indicates cumulative measurement uncertainty (on Raman pixel intensities and front position) and fitting uncertainty. Not all points could be fitted due to noise and are subsequently excluded. The voltage is not iR corrected.

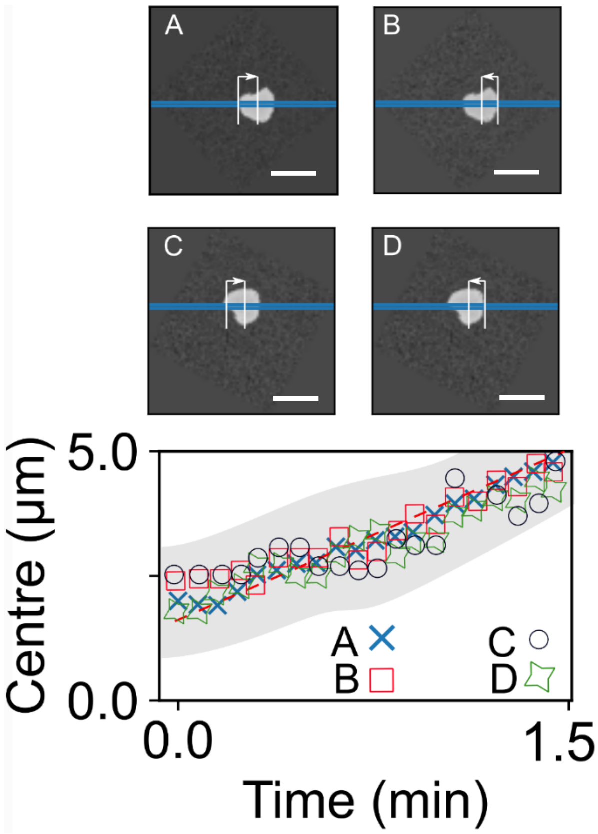

Supplementary Figure 48: Influence of particle boundary on the extracted front velocity from the first anodic scan of α-Li_2IrO_3 in KOH. (Top) Raman image of agglomerate particle integrating across 640 cm^{-1} mode in four different orientations (A – D). In each case the horizontal white arrow shows direction of moving front, which is measured to move between the agglomerate boundary and centre-of-mass (central white line). Blue lines show the region of pixels integrated across to improve the signal-to-noise ratio of the line-cuts. This is the same

width in all four orientations. (Bottom) After fitting a sigmoid to the line-cuts of the phase-boundary moving in the first anodic scan the centre of the phase-front can be estimated. The movement of this phase-front centre as a function of time is shown for all four edges examined in the top panels. The grey shading shows the overall measurement and fit uncertainty for all four orientations examined. A line-cut could not be fitted reliably for all time points in the scan due to noise and subsequently some points are excluded. The dashed red-line shows the mean change in the sigmoid centre over time. Scale bar is 5 μm.

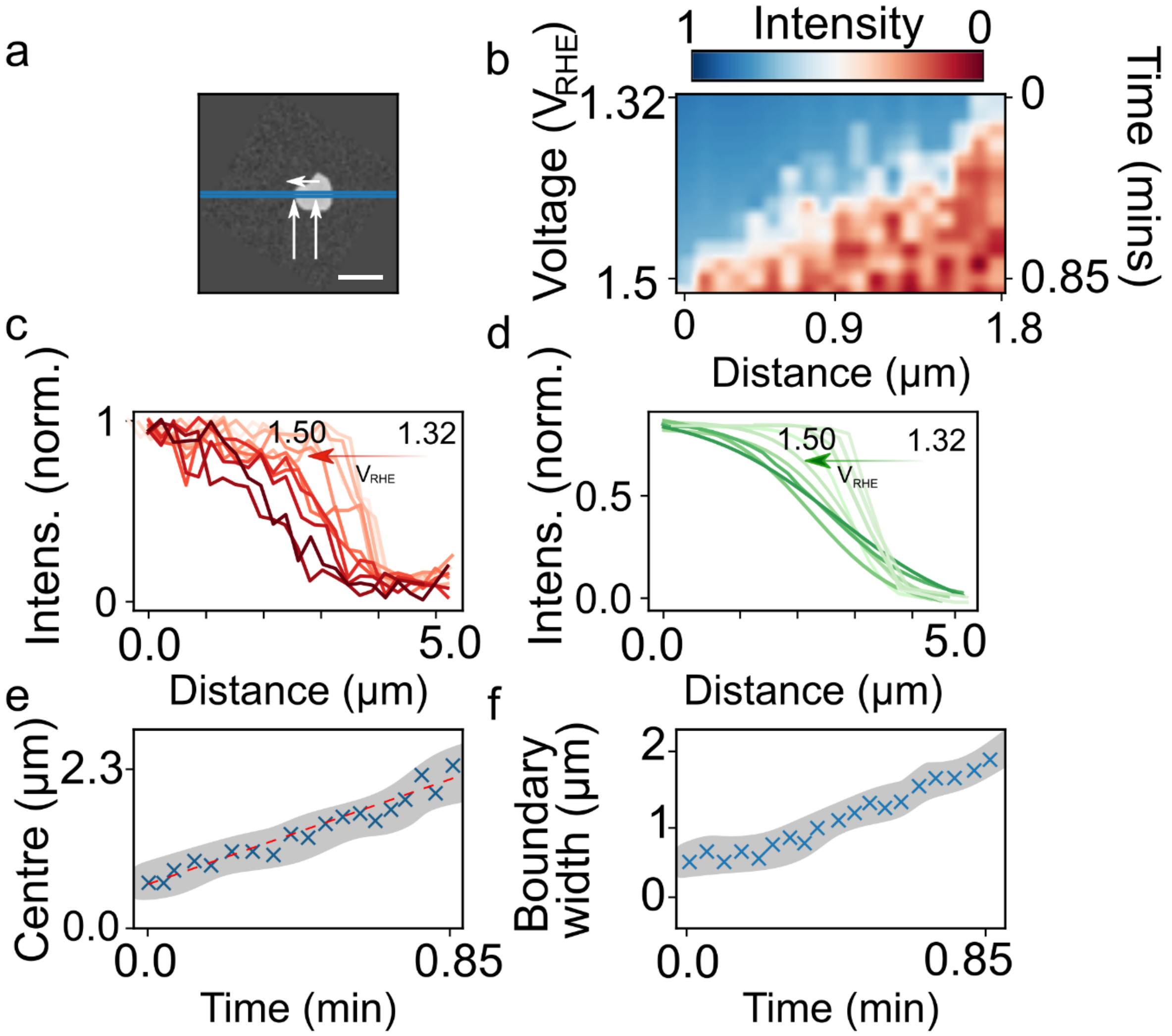

Supplementary Figure 49: Determining the mode front velocity during the second anodic scan of α-Li_2IrO_3 in KOH. **a.** Re-orientated Raman image of α-Li_2IrO_3 integrating across 640 cm^{-1} mode with Gaussian smoothing applied. Horizontal white arrow shows direction of moving front, which is measured to move between the agglomerate centre-of-mass (central white arrow) and boundary. Blue lines show the region of pixels integrated across to improve the signal-to-noise ratio of the line cuts. Scale bar is 5 μm. **b.** Normalised stacked line-cuts of Raman image (integrating across 640 cm^{-1}) as a function of time/potential for the second anodic scan of the material in KOH. **c.** Line-cuts across from map in **b** shown as line-plot. Not all line-cuts are shown. Arrow/shading indicates increasing potential/time. **d.** Fit of line-cuts shown in c using sigmoid function (see above for equation). Not all line-cuts are shown. Arrow/shading

indicates increasing potential/time**. e.** Position of fitted sigmoid as a function of time. Dotted red line shows straight-line fit from whose gradient the non-voltage normalised 'phase-front' velocity can be estimated. Grey shading indicates cumulative measurement uncertainty (on Raman pixel intensities and front position) and fitting uncertainty. Not all points could be fitted due to noise and are subsequently excluded. **f.** Phase-front boundary width, as estimated from the region in which the normalised sigmoid line cut lies between 0.2 and 0.8, as a function of time. Grey shading indicates cumulative measurement (on Raman pixel intensities and front position) and fitting uncertainty. Not all points could be fitted due to noise and are subsequently excluded. The voltage is not iR corrected.

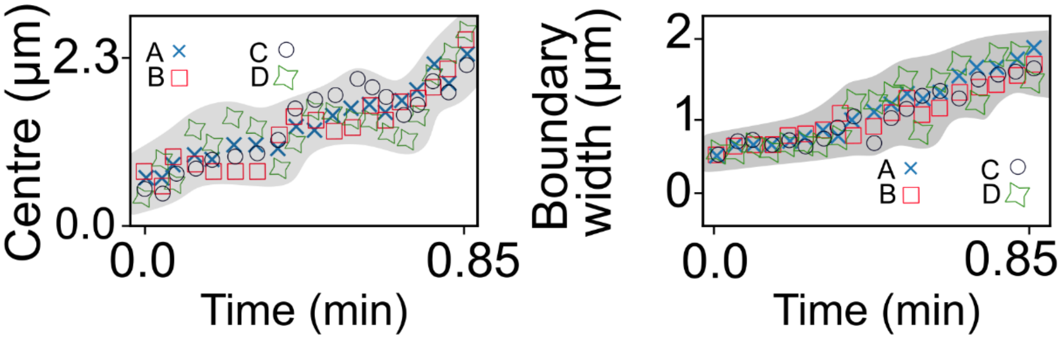

Supplementary Figure 50: Moving front velocity and width during the second anodic scan of α-Li_2IrO_3 in KOH. (Left) After fitting a sigmoid to the line-cuts of the phase-boundary moving in the second anodic scan, the centre of the phase-front can be estimated. The movement of this phase-front centre as a function of time is shown for all four directions (A – D) highlighted in the top panels of **Supplementary Figure 48**. The grey shading shows the overall measurement and fit uncertainty for all four orientations examined. A line-cut could not be fitted reliably for all time points in the scan due to noise and subsequently some points are excluded. (Right) The width of the phase-front boundary is also extracted as a function of time throughout scan by examining phase front motion in the four directions A – D shown in **Supplementary Figure 48**. The grey shading shows the overall measurement and fit uncertainty for all four orientations examined. A line-cut could not be fitted reliably for all time points in the scan due to noise and subsequently some points are excluded.

Finally, we note that whilst we fit a straight line to the phase front centre *vs* time graphs above to extract 'velocities', equations of the form t^n where n = 0.92 to 1.18, also generally fit the data well (with n depending on the exact data set). Practically, the exact fitting equation we use has little effect on the values we obtain for the phase front velocities, or any implication for our conclusions. This is because, the phase front values are only semi-quantitative and must be viewed in relation to one another due to the limitations of 2D imaging in an overall 3D system. It is for this reason we remain with a linear fit. Higher spatial and temporal resolution imaging in the future may be able to reveal the exact front propagation behaviour. We also emphasise the model in **Figure 4** of the main text is only qualitative and to capture the full behaviour in *e.g.*, a phase field model, additional factors would likely need to be considered (for example non-uniform diffusion rates throughout the particle volume *via* a point diffusion 'source').

Supplementary Note 16: Scan rate dependence of results

We note that in our previous work[25] *operando* XRD measurements were performed over a period of 5 to 6 hours to observe the change in structure of α-Li_2IrO_3 during the electrochemical cycling. This is in contrast to the high-speed imaging performed here where cycling and changes in the particles are observed over a period of minutes. However, it must be noted that the type of samples used between the XRD and Raman experiments are quite different. For the operando XRD measurements in ref[25], electrodes with a loading as large as 8 mg cm^{-2} were used to provide sufficient material to be detected by XRD (a technique sensitive only to bulk changes).

Furthermore, for the *operando* XRD measurements, the electrodes were prepared on carbon paper, which is known to be hydrophobic. Measurements were made with the electrode facing upwards with the electrolyte in contact only through the back surface and capillarity through the carbon paper. In order for the electrolyte to have sufficient time to reach the electrode, slow cycling followed by a potential hold of several hours, applied at the end of the operando XRD measurements, was needed. Indeed, to observe the K^+-intercalated phase by XRD, a significant amount of the powder used in the operando cell has to be intercalated with potassium.

Instead, the operando Raman imaging carried out in this study is made on single particles, with no carbon paper and with liquid electrolyte directly in contact with the particles. Raman experiments at a lower scan rate (0.4 mV s^{-1}) shown in **Supplementary Figure 51** highlight that similar results are observed to that in the main text confirming our conclusions on the two charge compensation pathways depending on the current density/applied potential and not on the timescale of the experiment. We note however that even at these slow scan rates conventional Raman spectroscopy methods are not usable due to the few hour acquisition times per image, high data loads and risks of sample damage (from the long exposure times) [53,54]. This highlights the power of the compressive imaging framework we use.

Finally, we note that a number of other groups have shown that at the individual object level cation (de)intercalation in oxides occurs several times faster than in bulk electrodes, due to mass transport and diffusion limitations in the latter. For example, in operando experiments of lithium (de)intercalation in lithium cobalt oxide Merryweather et al.[51] noted that whilst they

applied a current density of 6C, the single particle C-rates were 2 to 4 times higher at 23C and 13 C for delithiation and lithiation, respectively.

In summary, our initial results in Supplementary ref. 25 using *operando* XRD lacked (i) the time-resolution (due to XRD being sensitive to only bulk changes) to capture the competing OER mechanisms and (ii) the spatial-resolution (coupled with inherent mass transport limitations of bulk electrodes) to observe the mechanism progress through single particles as a function of applied potential.

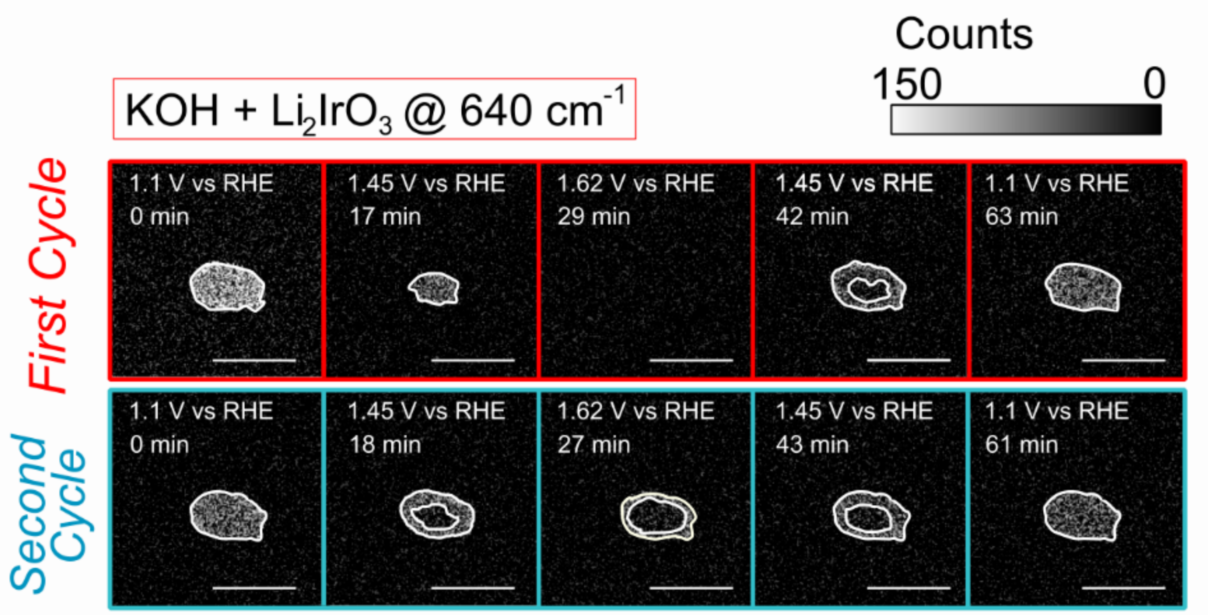

Supplementary Figure 51: Influence of scan rate on the Raman imaging patterns of α-Li_2IrO_3. Raman imaging during first two electrochemical cycles of α-Li_2IrO_3 in KOH at a scan rate of 0.4 mV s^{-1}, ten times lowers than that in the main text. The spatial propagation of intensity fronts is qualitatively similar to that observed at higher scan rates. The voltage is not iR corrected. Scale bar is 5 µm.

Although between 0.4 to 4 mV s^{-1} we observe no influence of scan rate on the mechanism from our Raman imaging (**Supplementary Figure 51**), at faster scan rates we might expect the spatial patterns and behaviour to be changed. However, this cannot be captured with our current Raman setup because the acquisition speed of our vibrational imaging (which is still well beyond the state-of-the-art, especially over fields of views in excess of 300 µm^2) is limited to imaging at scan-speeds of up to 10 mV s^{-1} (synchronised with the electrochemistry).

Nonetheless, to investigate this further we performed cyclic voltammetry of α-Li_2IrO_3 (after 10 activation cycles) at scan rates up to 500 mV s^{-1} in 1.0 M KOH electrolyte (**Supplementary**

Figure 52) without imaging. At high scan rates, one can first observe the growth of a reduction peak at ~1.43 V vs RHE, which is likely only due to the reduction of high oxidation state Ir species formed on the surface of the catalyst during the OER. Indeed, when increasing the scan rate, these oxidized Ir species are not fully consumed, due to an insufficient reaction rate or due to the slow kinetics for cation exchange and intercalation into the lattice of α-Li_xIrO_3. The OER current is lower at 0.4 mV s^{-1} compared to other scan rates, which is due to bubble accumulation at the surface of the catalyst.

Hence, electrochemistry alone also cannot reveal information regarding the kinetics of the charge compensation pathways during reaction of high oxidation Ir species with water.

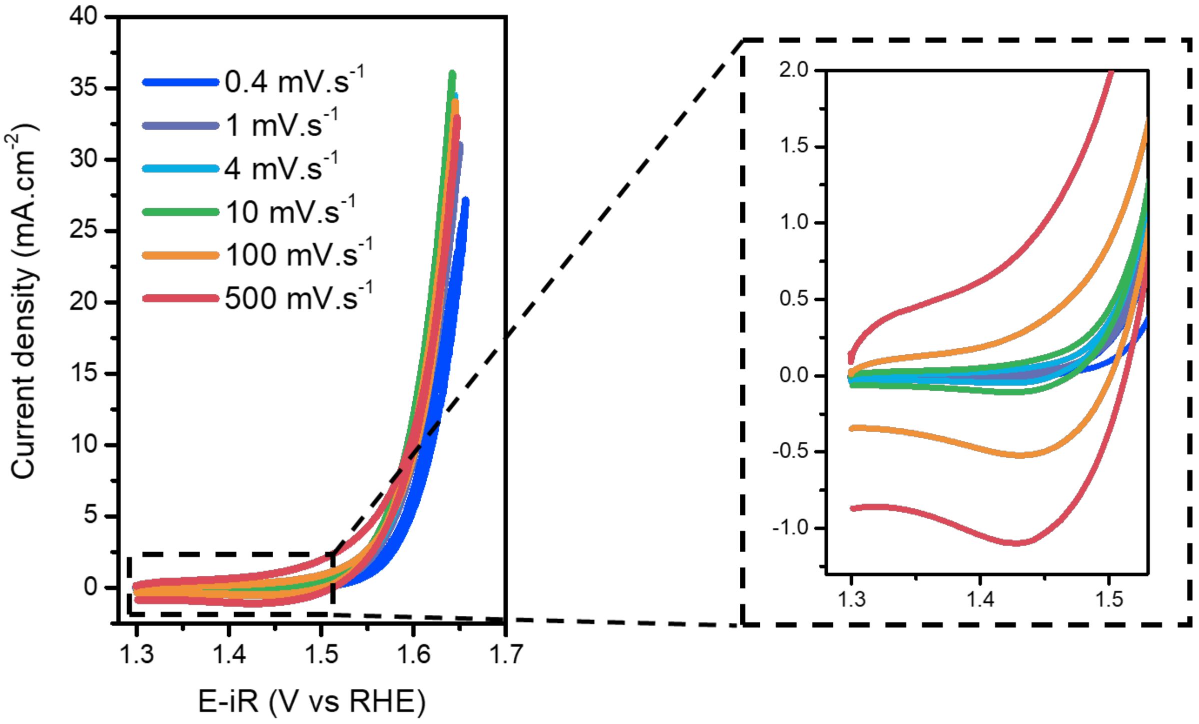

Supplementary Figure 52: Influence of scan rate on the cyclic voltammetry of α-Li_2IrO_3. Cyclic voltammetry of α-Li_2IrO_3 (after 10 activation cycles at 10 mV s^{-1} between 1.3 and 1.6 V vs RHE) at 0.4 mV s^{-1} (deep blue), 1 mV s^{-1} (blue), 4 mV s^{-1} (light blue), 10 mV s^{-1} (green), 100 mV s^{-1} (orange) and 500 mV s^{-1} (red) in a 1.0 M KOH electrolyte at a RDE (1600 rpm). The voltage is iR corrected.

Supplementary Note 17: Cost comparison between single pixel Raman spectrometers, EMCCD based Raman spectrometers and bright-field microscopes

The advantages/disadvantages of single pixel spectrometers in terms of sensitivity and cost specifically for Raman imaging have already been highlighted in previous studies[55]. Here, we compare Raman and bright-field (BF) optical microscopes. The latter has gained popularity for non-chemically specific characterisation of electrochemical materials[56]. Both Raman and bright-field microscopes can be broken down into: (i) an excitation source, (ii) a microscope body and (iii) a detection system. Both BF and Raman require near identical (scanning) microscope bodies with appropriate stages, high-resolution objectives, etc and hence on this front have comparable costs. The excitation source in Raman is a laser whereas in BF an LED, lamp or laser can be used depending on the exact experiment[51,57–59]. LED/lamp options typically represent approximately half the cost of those using a laser ($100 to $10,000 versus $5000 to $20,000 based on the equipment used from the referenced studies above). We note however, that a number of the BF studies do also use lasers[60]. Hence, we can also consider on excitation the two methods to be cost comparable. In BF a camera retailing between $500 and $30,000 is used for detection depending on the sensitivity needed[61]. In conventional Raman measurements an EMCCD and spectrograph is required costing between $30,000 and $50,000. However, our single pixel spectrometers replaces the EMCCD camera by a DMD ($500), an additional grating ($500) together with avalanche photodiode ($2000)[62]. This makes the compressive spectrometer an order-of magnitude cheaper than conventional spectrometers and at a comparable and competitive price point with the detectors used in BF microscopy. However, with the added benefits of quantitative chemically sensitive information across both solid and liquid phases, which can be readily interpreted and linked to physical parameters, in contrast to that from BF experiments. Hence, although the above analysis is simple we believe it highlights that Raman imaging particularly with our single-pixel computational framework can be a competitive low-cost option for label-free characterisation of electrochemical systems.

Finally, it is important to note that compressive sensing has been applied to Raman imaging before (see refs[1,55,63,64]). In this work we push the method in three specific ways:

1. The imaging speeds and associated signal-to-noise ratios we achieve (pixel dwell times in the microsecond regime) are significantly higher than previous implementations. Spontaneous Raman methods are key for exploring the wavenumber regime (<1000 cm^{-1}) of electrochemical systems, a regime that is not generally accessible using the more developed (and significantly more costly) stimulated Raman imaging.

2. Previous applications of compressive sensing have been proof-of-concept on static systems e.g. plastic beads, bio-minerals, etc. We demonstrate that the compressive Raman imaging can be practically used to visualise high-speed dynamics.
3. Instrument development in-terms of substrates, synchronization with electrochemistry, imaging geometry, etc has been performed to allow for such measurements.

Supplementary Note 18: Additional details on experimental setup

Supplementary Figure 53 shows some examples of our experimental setup. An Ag wire (0.5 mm diameter; Sigma) was attached to the conductive coverslide using epoxy glue and conductive copper tape to allow connection to the working electrode. The reference (Ag/AgCl porous frit electrode; redox.me) and counter (Pt wire; 0.5 mm thickness; Sigma) were well separated and the reference was held just above the surface of the coverslides with micrometre screw gauges used to achieving exact positioning. Silicone rectangular or circular shaped wells (Mcmaster Carr) with a predefined area were bonded to the sample such that the active area with electrolyte could be determined. Wells were filled with the given electrolyte (200 μL to ~1500 μL depending on the experiment) to ensure all electrodes were wet. Potentials were then applied as detailed in the main text. For experiments requiring long-term (> 30 mins) focus stability, a line based[65] or phase imaging based[66] autofocus at 405 nm was used.

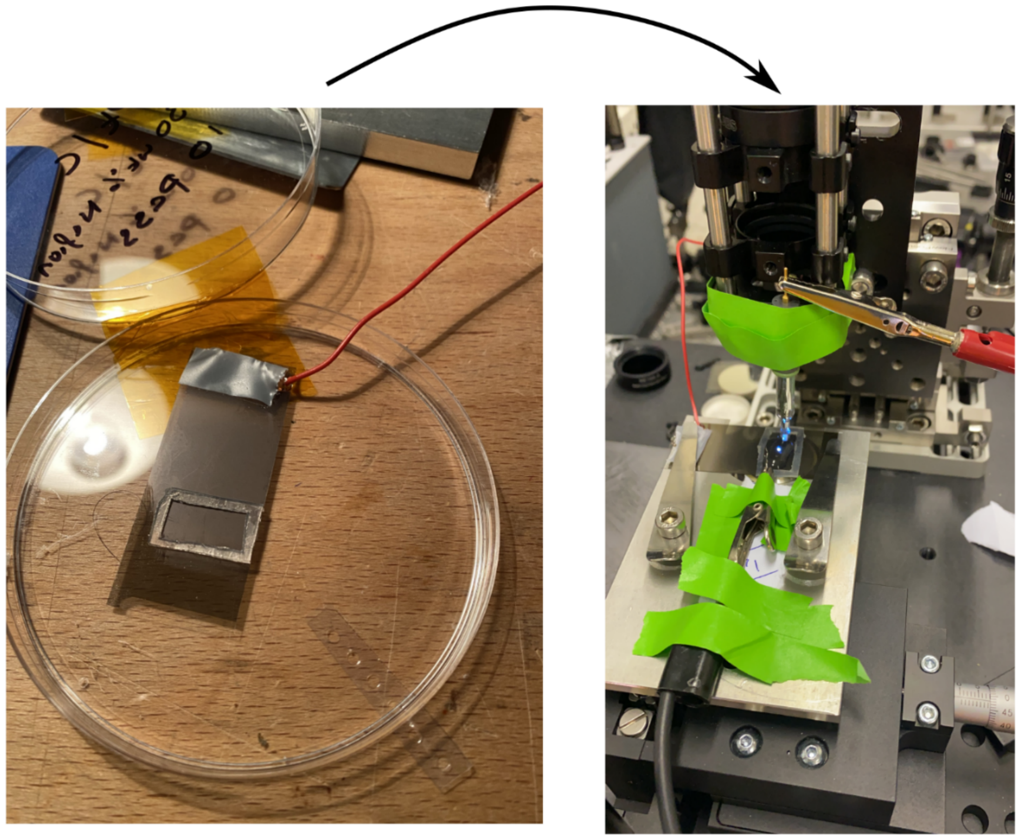

Supplementary Figure 53: Digital photographs of samples and experimental setup. Example of conductive coverslide (in this case with electrodeposited Nafion only), with a wire bonded to allow electrical connection to working electrode (left). The coverslide is then mounted onto the microscope with the Pt counter (connected to black crocodile clip) and

reference (connected to red crocodile clip) dipped into the well with electrolyte. Imaging can then be performed with epi-illumination.

Supplementary References

1. Sturm, B. *et al.* High-Sensitivity High-Speed Compressive Spectrometer for Raman Imaging. *ACS Photonics* **6**, 25 (2019).
2. Soldevila, F., Dong, J., Tajahuerce, E., Gigan, S. & De Aguiar, H. B. Fast compressive Raman bio-imaging via matrix completion. *Optica* **6**, 341–346 (2019).
3. Mankani, B. R. *et al.* Binary Complementary Filters for Compressive Raman Spectroscopy. *Appl. Spectrosc. Vol. 72, Issue 1, pp. 69-78* **72**, 69–78 (2018).
4. Lin, H. & B. de Aguiar, H. Compressive Raman microspectroscopy . in *Stimulated Raman Scattering Microscopy* (2022).
5. Senthilkumar, M., Mathiyarasu, J., Joseph, J., Phani, K. L. N. & Yegnaraman, V. Electrochemical instability of indium tin oxide (ITO) glass in acidic pH range during cathodic polarization. *Mater. Chem. Phys.* **108**, 403–407 (2008).
6. Diel, E. E., Lichtman, J. W. & Richardson, D. S. Tutorial: avoiding and correcting sample-induced spherical aberration artifacts in 3D fluorescence microscopy. *Nat. Protoc. 2020 159* **15**, 2773–2784 (2020).
7. Ghosh, B., Mandal, M., Mitra, P. & Chatterjee, J. Attenuation corrected-optical coherence tomography for quantitative assessment of skin wound healing and scar morphology. *J. Biomed. Opt* **25**, 40901 (2020).
8. Sachse, R. *et al.* Assessing Optical and Electrical Properties of Highly Active IrO xCatalysts for the Electrochemical Oxygen Evolution Reaction via Spectroscopic Ellipsometry. *ACS Catal.* **10**, 14210–14223 (2020).
9. Backholm, J. & Niklasson, G. A. Optical properties of electrochromic iridium oxide and iridium–tantalum oxide thin films in different colouration states. *Sol. Energy Mater. Sol. Cells* **92**, 1388–1392 (2008).
10. Gong, P. *et al.* Parametric imaging of attenuation by optical coherence tomography: review of models, methods, and clinical translation. *J. Biomed. Opt* **25**, 40901 (2020).
11. Pandya, R. *et al.* Three-dimensional operando optical imaging of particle and electrolyte heterogeneities inside Li-ion batteries. *Nat. Nanotechnol. 2023* **18**, 1185–1194 (2023).
12. Wang, J., Chen-Wiegart, Y. C. K., Eng, C., Shen, Q. & Wang, J. Visualization of anisotropic-isotropic phase transformation dynamics in battery electrode particles. *Nat. Commun. 2016 71* **7**, 1–7 (2016).
13. Brunetti, G. *et al.* Confirmation of the domino-cascade model by lifepo4/fepo 4

precession electron diffraction. *Chem. Mater.* **23**, 4515–4524 (2011).

14. Hyun, H. *et al.* Suppressing High-Current-Induced Phase Separation in Ni-Rich Layered Oxides by Electrochemically Manipulating Dynamic Lithium Distribution. *Adv. Mater.* **33**, 2105337 (2021).
15. Ge, M. *et al.* Kinetic Limitations in Single-Crystal High-Nickel Cathodes. *Angew. Chemie Int. Ed.* **60**, 17350–17355 (2021).
16. Park, J. *et al.* Fictitious phase separation in Li layered oxides driven by electro-autocatalysis. *Nat. Mater.* **20**, 991–999 (2021).
17. Delmas, C., Maccario, M., Croguennec, L., Le Cras, F. & Weill, F. Lithium deintercalation in LiFePO4 nanoparticles via a domino-cascade model. *Nat. Mater. 2008 78* **7**, 665–671 (2008).
18. Guo, M. & White, R. E. An approximate solution for solid-phase diffusion in a spherical particle in physics-based Li-ion cell models. *J. Power Sources* **198**, 322–328 (2012).
19. Radha, S. K. *et al.* Optical response and band structure of LiCoO2 including electron-hole interaction effects. *Phys. Rev. B* **104**, 115120 (2021).
20. Liu, H. L. *et al.* Electronic structure and lattice dynamics of LixCoO2 single crystals. *New J. Phys.* **17**, 103004 (2015).
21. Dovesi, R. *et al.* CRYSTAL17 User's Manual. (2018).
22. Dovesi, R. *et al.* Quantum-mechanical condensed matter simulations with CRYSTAL. *Wiley Interdiscip. Rev. Comput. Mol. Sci.* **8**, e1360 (2018).
23. Ping, Y., Galli, G. & Goddard, W. A. Electronic structure of IrO2: The role of the metal d orbitals. *J. Phys. Chem. C* **119**, 11570–11577 (2015).
24. Laun, J. & Bredow, T. BSSE-corrected consistent Gaussian basis sets of triple-zeta valence with polarization quality of the fifth period for solid-state calculations. *J. Comput. Chem.* **43**, 839–846 (2022).
25. Yang, C. *et al.* Cation insertion to break the activity/stability relationship for highly active oxygen evolution reaction catalyst. *Nat. Commun. 2020 111* **11**, 1–10 (2020).
26. Giannozzi, P. *et al.* QUANTUM ESPRESSO: a modular and open-source software project for quantum simulations of materials. *J. Phys. Condens. Matter* **21**, 395502 (2009).
27. Giannozzi, P. *et al.* Advanced capabilities for materials modelling with Quantum ESPRESSO. *J. Phys. Condens. Matter* **29**, 465901 (2017).

28. Lejaeghere, K. *et al*. Reproducibility in density functional theory calculations of solids. *Science* **351**, (2016).
29. Prandini, G., Marrazzo, A., Castelli, I. E., Mounet, N. & Marzari, N. Precision and efficiency in solid-state pseudopotential calculations. *npj Comput. Mater. 2018 41* **4**, 1–13 (2018).
30. Jacob, C. R. & Reiher, M. Spin in density-functional theory. *Int. J. Quantum Chem.* **112**, 3661–3684 (2012).
31. Harvey, J. N. On the accuracy of density functional theory in transition metal chemistry. *Annu. Reports Sect. 'C' (Physical Chem.* **102**, 203–226 (2006).
32. Cramer, C. J. & Truhlar, D. G. Density functional theory for transition metals and transition metal chemistry. *Phys. Chem. Chem. Phys.* **11**, 10757–10816 (2009).
33. Lazzeri, M. & Mauri, F. First-Principles Calculation of Vibrational Raman Spectra in Large Systems: Signature of Small Rings in Crystalline [Formula presented]. *Phys. Rev. Lett.* **90**, 4 (2003).
34. Bartlett, R. J. & Musiał, M. Coupled-cluster theory in quantum chemistry. *Rev. Mod. Phys.* **79**, 291–352 (2007).
35. Zhang, J., Zhang, H., Wu, T., Wang, Q. & Van Der Spoel, D. Comparison of Implicit and Explicit Solvent Models for the Calculation of Solvation Free Energy in Organic Solvents. *J. Chem. Theory Comput.* **13**, 1034–1043 (2017).
36. Flores, E., Novák, P. & Berg, E. J. In situ and Operando Raman spectroscopy of layered transition metal oxides for Li-ion battery cathodes. *Front. Energy Res.* **6**, 82 (2018).
37. Flores, E., Vonrüti, N., Novák, P., Aschauer, U. & Berg, E. J. Elucidation of LixNi0.8Co0.15Al0.05O2 Redox Chemistry by Operando Raman Spectroscopy. *Chem. Mater.* **30**, 4694–4703 (2018).
38. Pavlovic, Z., Ranjan, C., Gao, Q., Van Gastel, M. & Schlögl, R. Probing the structure of a water-Oxidizing anodic iridium oxide catalyst using raman spectroscopy. *ACS Catal.* **6**, 8098–8105 (2016).
39. Huang, Y. S. *et al*. Raman spectrum of IrO2. *Solid State Commun.* **70**, 517–522 (1989).
40. Gao, J. *et al*. Breaking Long-Range Order in Iridium Oxide by Alkali Ion for Efficient Water Oxidation. *J. Am. Chem. Soc.* **141**, 3014-–3023 (2019).
41. Cheng, Q., Yang, T., Li, Y., Li, M. & Chan, C. K. Oxidation–reduction assisted

exfoliation of LiCoO2 into nanosheets and reassembly into functional Li-ion battery cathodes. *J. Mater. Chem. A* **4**, 6902–6910 (2016).

42. Kim, Y. *et al.* Alteration of oxygen evolution mechanisms in layered LiCoO2 structures by intercalation of alkali metal ions. *J. Mater. Chem. A* **10**, 10967–10978 (2022).
43. Jiang, S. *et al.* Lightest Metal Leads to Big Change: Lithium-Mediated Metal Oxides for Oxygen Evolution Reaction. *Adv. Energy Mater.* **12**, 2201934 (2022).
44. Gross, T. & Hess, C. Raman diagnostics of LiCoO2 electrodes for lithium-ion batteries. *J. Power Sources* **256**, 220–225 (2014).
45. Matsuda, Y. *et al.* In situ Raman spectroscopy of LixCoO2 cathode in Li/Li3PO4/LiCoO2 all-solid-state thin-film lithium battery. *Solid State Ionics* **335**, 7–14 (2019).
46. Lemineur, J. F. *et al.* Imaging and Quantifying the Formation of Single Nanobubbles at Single Platinum Nanoparticles during the Hydrogen Evolution Reaction. *ACS Nano* **15**, 2643–2653 (2021).
47. Illingworth, J. & Kittler, J. The Adaptive Hough Transform. *IEEE Trans. Pattern Anal. Mach. Intell.* **PAMI-9**, 690–698 (1987).
48. Zhang, T. *et al.* Revealing the Activity Distribution of a Single Nanocatalyst by Locating Single Nanobubbles with Super-Resolution Microscopy. *J. Phys. Chem. Lett.* **9**, 5630–5635 (2018).
49. German, S. R., Edwards, M. A., Chen, Q. & White, H. S. Laplace Pressure of Individual H2 Nanobubbles from Pressure-Addition Electrochemistry. *Nano Lett.* **16**, 6691–6694 (2016).
50. Rivnay, J. *et al.* Structural control of mixed ionic and electronic transport in conducting polymers. *Nat. Commun.* **7**, 1–9 (2016).
51. Merryweather, A. J., Schnedermann, C., Jacquet, Q., Grey, C. P. & Rao, A. Operando optical tracking of single-particle ion dynamics in batteries. *Nature* **594**, 522–528 (2021).
52. Wang, B. & Fan, S. An improved CANNY edge detection algorithm. *2nd Int. Work. Comput. Sci. Eng. WCSE 2009* **1**, 497–500 (2009).
53. Krafft, C. *et al.* Label-Free Molecular Imaging of Biological Cells and Tissues by Linear and Nonlinear Raman Spectroscopic Approaches. *Angew. Chemie Int. Ed.* **56**, 4392–4430 (2017).

54. Stavitski, E. & Weckhuysen, B. M. Infrared and Raman imaging of heterogeneous catalystsw. *Chem. Soc. Rev* **39**, 4615 (2010).
55. Scotté, C. *et al.* Assessment of Compressive Raman versus Hyperspectral Raman for Microcalcification Chemical Imaging. *Anal. Chem.* **90**, 7197–7203 (2018).
56. Meyer, L., Saqib, N. & Porter, J. Review—Operando Optical Spectroscopy Studies of Batteries. *J. Electrochem. Soc.* **168**, 090561 (2021).
57. Sanchez, A. J., Kazyak, E., Chen, Y., Lasso, J. & Dasgupta, N. P. Lithium stripping: anisotropic evolution and faceting of pits revealed by operando 3-D microscopy. *J. Mater. Chem. A* **9**, 21013–21023 (2021).
58. Godeffroy, L. *et al.* Bridging the Gap between Single Nanoparticle Imaging and Global Electrochemical Response by Correlative Microscopy Assisted By Machine Vision. *Small Methods* **2200659**, 1–12 (2022).
59. Ye, X., Saqib, M., Mao, J., Li, G. & Hao, R. Spatiotemporally super-resolved dendrites nucleation and early-stage growth dynamics in Zinc-ion batteries. *Cell Reports Phys. Sci.* **2**, 100420 (2021).
60. Merryweather, A. J. *et al.* Operando monitoring of single-particle kinetic state-of-charge heterogeneities and cracking in high-rate Li-ion anodes. *Nat. Mater.* **21**, 1306–1313 (2022).
61. Salido, J., Bueno, G., Ruiz-Santaquiteria, J. & Cristobal, G. A review on low-cost microscopes for Open Science. *Microsc. Res. Tech.* **85**, 3270–3283 (2022).
62. Musiienko, A., Žídek, K. & Denk, O. Differential single-pixel camera enabling low-cost microscopy in near-infrared spectral region. *Opt. Express, Vol. 27, Issue 4, pp. 4562-4571* **27**, 4562–4571 (2019).
63. Wilcox, D. S. *et al.* Digital compressive chemical quantitation and hyperspectral imaging. *Analyst* **138**, 4982–4990 (2013).
64. Cebeci, D., Mankani, B. R. & Ben-Amotz, D. Recent Trends in Compressive Raman Spectroscopy Using DMD-Based Binary Detection. *J. Imaging* **5**, 1 (2018).
65. Bian, Z. *et al.* Autofocusing technologies for whole slide imaging and automated microscopy. *J. Biophotonics* **13**, e202000227 (2020).
66. Silvestri, L. *et al.* Universal autofocus for quantitative volumetric microscopy of whole mouse brains. *Nat. Methods 2021 188* **18**, 953–958 (2021).
