# Supplementary material for: Concurrent oxygen evolution reaction pathways revealed by high-speed compressive Raman imaging
Source: Nat Commun. 2024 Sep 27;15:8362. doi: 10.1038/s41467-024-52536-7 (PMC11437135; doi:10.1038/s41467-024-52536-7)
Supplement: Supplementary file 3 — Description of Additional Supplementary Files [file 41467_2024_52536_MOESM3_ESM.docx]

**Supplementary Video 1:** Video showing spatial intensity of 640 cm^-1^ mode as a function of potential/current/time for 7 cycles of particle shown in main text. Mode intensity is arbitrary, and particle size is the same as that shown in main text Figure 3.

**Supplementary Video 2:** Video showing spatial intensity of 550 cm^-1^ mode as a function of potential/current/time for 7 cycles of particle shown in main text. Mode intensity is arbitrary and particle size is the same as that shown in main text Figure 3

**Supplementary Video 3:** Video showing spatial intensity of 640 cm^-1^ mode as a function of potential/current/time for 7 cycles of a second particle to that shown in main text. Mode intensity is arbitrary and particle size is same as that shown in Supplementary Figure 41.

**Supplementary Video 4:** Video showing spatial intensity of 550 cm^-1^ mode as a function of potential/current/time for 7 cycles of a second particle to that shown in main text. Mode intensity is arbitrary and particle size is same as that shown in Supplementary Figure 40.

**Supplementary Video 5:** Video showing spatial intensity of 640 cm^-1^ mode as a function of current/time for particle held at 1.7 vs RHE for 2 mins as depicted in main text. Mode intensity is arbitrary and particle size is the same as that shown in main text Figure 5.

**Supplementary Video 6:** Video showing spatial intensity of 640 cm^-1^ mode as a function of current/time for particle held at 1.6 vs RHE for 2 mins as depicted in main text. Mode intensity is arbitrary and particle size is the same as that shown in main text Figure 5.

**Supplementary Video 7:** Video showing spatial intensity of 640 cm^-1^ mode as a function of current/time for particle held at 1.5 vs RHE for 2 mins as depicted in main text. Mode intensity is arbitrary and particle size is the same as that shown in main text Figure 5.

**Supplementary Video 8:** Video showing spatial intensity of 640 cm^-1^ mode as a function of current/time for particle held at 1.4 vs RHE for 2 mins as depicted in main text. Mode intensity is arbitrary and particle size is the same as that shown in main text Figure 5.
